# Molecular Hybrids of Quinoline and Sulfonamide: Design, Synthesis and *in Vitro* Anticancer Studies

**DOI:** 10.1002/open.202400334

**Published:** 2024-11-26

**Authors:** Padyala Panduranga, Parameshwar Makam, Naresh Kumar Katari, Rambabu Gundla, Sreekantha Babu Jonnalagadda, Bharat Kumar Tripuramallu

**Affiliations:** ^1^ Department of Chemistry VFSTR (Deemed to be University) Vadlamudi, Guntur, Andhra Pradesh 522213 India; ^2^ Division of Research and Innovation Department of Chemistry Uttaranchal University,Arcadia Grant, P.O. Chandanwari Premnagar, Dehradun Uttarakhand 248007 India; ^3^ Department of Chemistry GITAM School of Science GITAM Deemed to be University Hyderabad, Telangana 502329 India; ^4^ School of Chemistry & Physics College of Agriculture Engineering & Science Westville Campus University of KwaZulu-Natal P Bag X 54001 Durban 4000 South Africa

**Keywords:** Quinoline, Sulfonamide, Molecular hybrids, Synthesis and in vitro anticancer study

## Abstract

Molecular hybrids of diversely functionalized quinoline and sulfonamide have been designed. Multistep synthetic strategies have been used for the synthesis. The anti‐cancer properties have been evaluated against various cancer cell lines including HCT116, A549, U2OS, CCRF‐CEM, Jurkat, MOLT‐4, RAMOS, and K562. Non‐cancer cell lines MRC‐5 and BJ were also included for comparison. When examining the effects on A549, HCT116, and U2OS cells, all tested compounds exhibited limited potency with IC_50_ values exceeding 50 μM, indicating weak activity against these cell lines. Against the ITK high cells *Viz*. are Jurkat, CCRF‐CEM and MOLT‐4, **9 e, 9 p** and **9 j** found to the maximum potent compounds with IC_50_ values of 7.43±7.40 μM, 13.19±1.25 μM and 5.57±7.56 μM respectively. Similarly, in the BTK high cells screenings, **9 n** and **9 e** molecules with an IC_50_ value of 2.76±0.79 μM and 5.47±1.71 μM against RAMOS and K562 respectively are highly potent. Interestingly, all the molecules have exhibited IC_50_ value >50 μM against the non‐cancer cells (MRC‐5 and BJ), which indicates the promising non‐cytotoxic nature of the molecules.

## Graphical abstract

Diversely functionalized molecular hybrids of quinoline and sulfonamide are designed. Interestingly, all the molecules have exhibited IC_50_ value >50 against the non‐cancer cells (MRC‐5 and BJ), which indicates the promising non‐cytotoxic nature of the molecules.

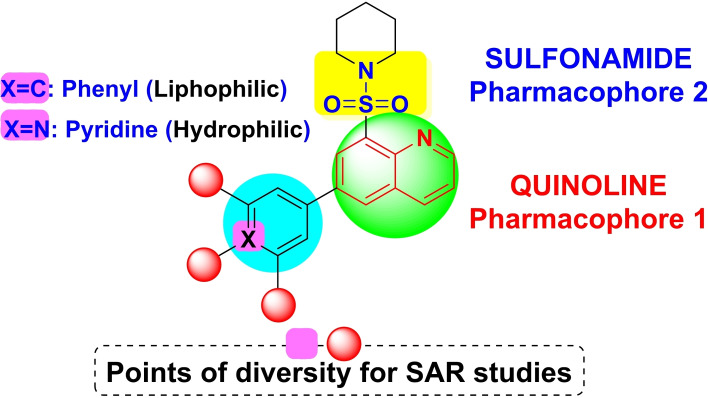

## 1 Introduction

Cancer constitutes a wide spectrum of diseases that emerge in diverse bodily organs or tissues. These ailments are characterized by the uncontrolled proliferation of abnormal cells, which breach their usual constraints, infiltrate neighboring body structures, and sometimes spread to distant organs—a phenomenon referred to as metastasis, often leading to fatal consequences.^[1]^ In terms of worldwide mortality, cancer holds a significant position, being accountable for approximately 10 million fatalities in the year 2020. This translates to nearly one out of every six reported deaths. Prominent variations of cancer encompass breast, lung, colorectal, and prostate cancers.^[2]^ At present, advent of drug resistance and occurrence of unintended effects associated with anti‐cancer drugs represent significant hurdles in the realm of cancer treatment. This dynamic compels medicinal chemists to persistently craft novel anti‐cancer agents that exhibit heightened efficacy while maintaining minimal toxicity.

**Figure 1:**
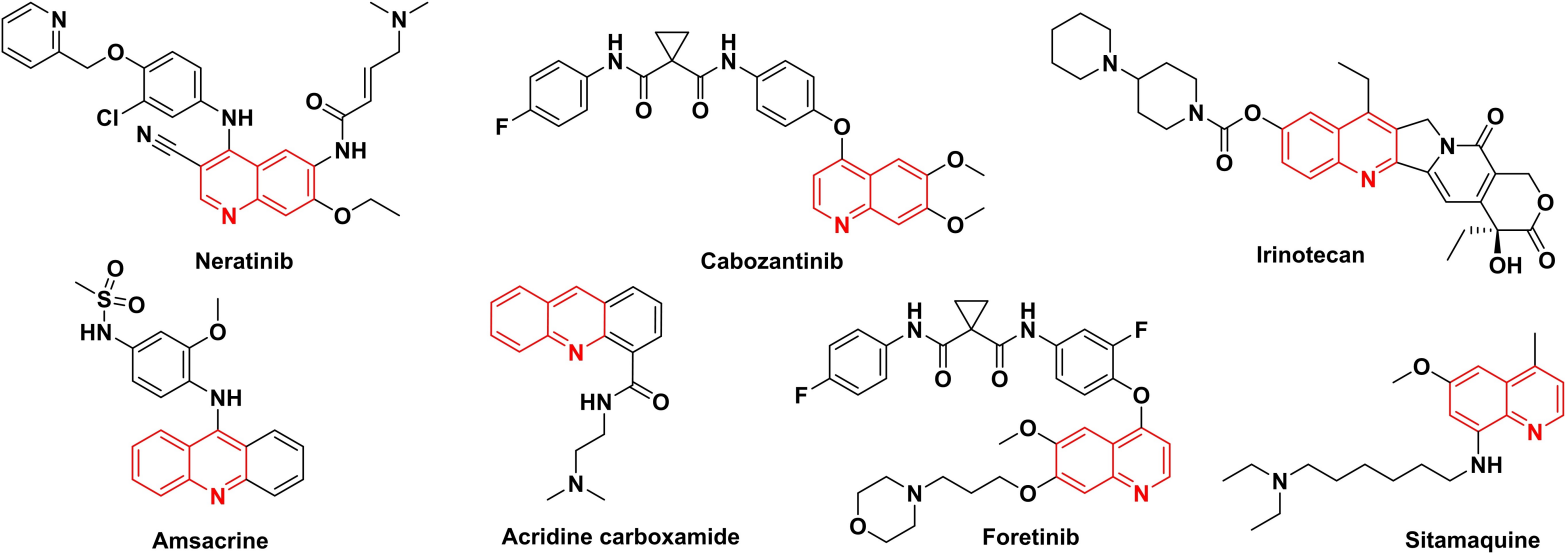
Anticancer agents of quinoline derivatives.

The quinoline forms with fusion of a pyridine and a benzene ring at two adjacent carbons.^[3]^ Quinoline, a privileged scaffold for medicinal and pharmaceutical researchers,^[4]^ exhibit a diverse array of pharmacological activities,^[3b]^ including analgesic,^[5]^ anti‐alzheimer, antibacterial,^[6]^ anti‐cancer,^[7]^ anti‐convulsant,^[3a]^ antifungal,^[8]^ anti‐inflammatory,^[9]^ antimalarial,^[10]^ antimicrobial,^[8]^ anti‐oxidant, antitubercular^[11]^ activities, among others. Numerous anticancer drugs based on quinoline derivatives are readily available as shown in Figure 1, functioning through diverse mechanisms that target various molecular entities. These mechanisms encompass the topoisomerase inhibition, tyrosine kinases suppression, interference with heat shock protein 90, restraint of histone deacetylases, cell cycle progression hindrance and induction of apoptosis, as well as the inhibition of tubulin polymerization.[^7^, ^10^, ^12^]

**Figure 2:**
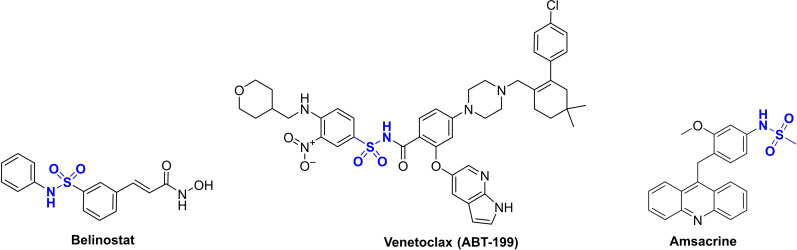
FDA approved Anticancer agents of sulfonamide derivatives.

The sulfonamide moiety has garnered substantial favor within medicinal chemistry, prompting the development of an array of sulfonamide derivatives exhibiting diverse biological activities.^[13]^ These activities encompass inhibition against bacteria,^[14]^ fungi,^[15]^ oxidants,^[16]^ inflammation,^[17]^ diabetic,^[18]^ and cancer properties.[^13^, ^19^] Notably, several sulfonamide derivatives have obtained FDA cancer therapy approvals. For instance, Belinostat, an inhibitor of histone deacetylase, has been sanctioned as the third drug for treating T‐cell lymphoma, following Romidepsin and Vorinostat.^[20]^ Bcl‐2 inhibitor, ABT‐199, is now authorized for treating patients with chronic lymphocytic leukemia.^[21]^ Amsacrine, has gained approval for combatting acute leukemias and malignant lymphomas by a proved topoisomerase‐II inhibition and by tumor DNA cell intercalation.^[22]^ The anticancer agents of sulfonamide derivatives are presented in Figure 2.

## 2 Results and Discussions

### 2.1 Design and Physico‐Chemical Properties Evaluation

Molecular hybridization stands as a groundbreaking paradigm within the domain of drug design and development. This pioneering strategy entails amalgamating pharmacophoric constituents derived from disparate bioactive compounds, culminating in novel hybrid molecules that showcase heightened affinity and efficacy in comparison to their original precursors. Furthermore, this approach bears the promise of generating compounds enriched with a revised selectivity spectrum, divergent or dual mechanisms of action, and a reduced incidence of undesired side effects.^[23]^ The global scientific community has observed the triumphant implementation of molecular hybridization by varied research collectives, resulting in promising advancements, particularly in the context of conditions marked by multifaceted disease profiles such as Alzheimer's, Parkinson's, inflammation, cancer and hypertension.^[24]^

**Figure 3:**
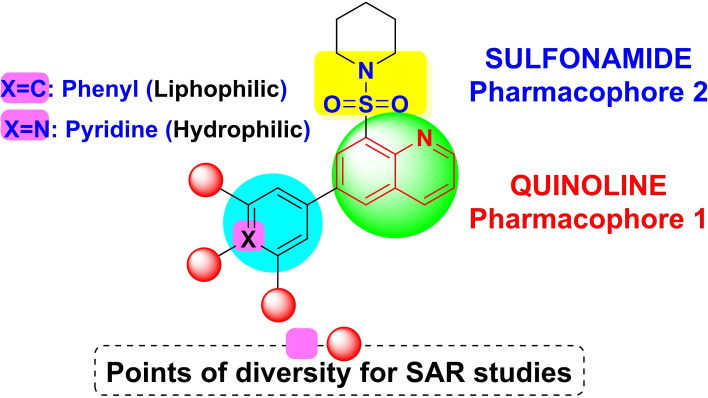
Molecular hybridization approach of quinoline and sulfonamide.

In the realm of medicinal chemistry, the sulfonamide moiety serves as a valuable bioisostere for carboxylic group. Specifically, the sulfonamide core can establish a hydrogen bond network analogous to that of the carboxylic group. Moreover, the spatial arrangement between the two atoms of oxygen in two functional groups is comparably similar.^[25]^ Serving as a bioisostere, the sulfonamide group can circumvent certain limitations associated with carboxylic group, including toxicity, metabolic instability and constrained passive diffusion across biological membranes^[25b]^. Given this backdrop, our curiosity was piqued to orchestrate the conception, synthesis, and comprehensive characterization of both the physicochemical attributes and the anti‐cancer properties inherent in molecular hybrids originating from quinoline‐based sulfonamide pharmacophore derivatives as shown in Figure 3. By using this method of molecular hybridization, the molecular hybrids of sulfonamide and quinoline have been created (**9 a** to **9 p**), with the sites of diversity located on the quinoline moiety's sixth position, containing hydrophophilic pyridyl and liphophilic phenyl systems. Phenyl and pyridyl groups can be further substituted with halogens, hydroxy and methoxy, amine, and cyano groups to create compounds with a wider variety of physico‐chemical properties. The molecules structures of 9a to 9p are illustrated in Table 2.

Compounds **9 a** and **9 g** exhibit molecular weights within the range of 352.45 to 437.34. Notably, **9 e** has the lowest iLogP value, while **9 l** possesses the highest, resulting in iLogP values spanning from 2.6 to 3.42. The hydrogen bond donor (HD) count varies from 0 to 1, and the hydrogen bond acceptor (HA) count ranges from 4 to 8 across the compounds. The presence of rotatable bonds falls between 3 to 5 for all compounds. Moreover, the PTSA values for these compounds lie within the range of 58.65 to 88.11. The compounds exhibit diverse physic‐chemical characteristics, aligning well with the criteria proposed by Lipinski, Veber, and Leeson. These findings are consolidated in Table 1 and Table 2.

**Table 1:** Physicochemical characteristics of quinoline and sulfonamide molecular hybrids.

Molecule	Physico‐chemical properties									Violation	Pharmacokinetic Properties		
	MW	HA	AHA	RBs	HA	HB	MR	TPSA	iLOGP		GI absorption	BBB permeant	PGP substrate
9a	352.45	25	16	3	4	0	104.2	58.65	2.73	0	High	Yes	No
9b	386.9	26	16	3	4	0	109.21	58.65	3.27	0	High	Yes	No
9c	370.44	26	16	3	5	0	104.16	58.65	2.88	0	High	Yes	No
9d	431.35	26	16	3	4	0	111.9	58.65	3.15	0	High	No	No
9e	368.45	26	16	3	5	1	106.22	78.88	2.6	0	High	No	Yes
9f	386.44	27	16	3	6	1	106.18	78.88	2.88	0	High	No	Yes
9g	437.34	28	16	3	5	1	116.24	78.88	3.12	0	High	No	No
9h	432.92	29	16	4	6	1	117.72	88.11	3.26	0	High	No	No
9i	382.48	27	16	4	5	0	110.69	67.88	3.24	0	High	Yes	No
9j	403.93	27	16	3	5	1	114.73	86.29	0	0	High	No	Yes
9k	377.46	27	16	3	5	0	108.91	82.44	2.95	0	High	No	No
9l	436.45	30	16	5	8	0	110.88	67.88	3.42	0	Low	No	No
9m	420.45	29	16	4	7	0	109.2	58.65	2.99	0	Low	No	No
9n	371.43	26	16	3	6	0	101.95	71.54	2.72	0	High	No	No
9o	383.46	27	16	4	6	0	108.48	80.77	2.87	0	High	No	No
9p	428.94	29	16	3	6	1	128.87	87.62	0	0	High	No	Yes

**Table 2:**
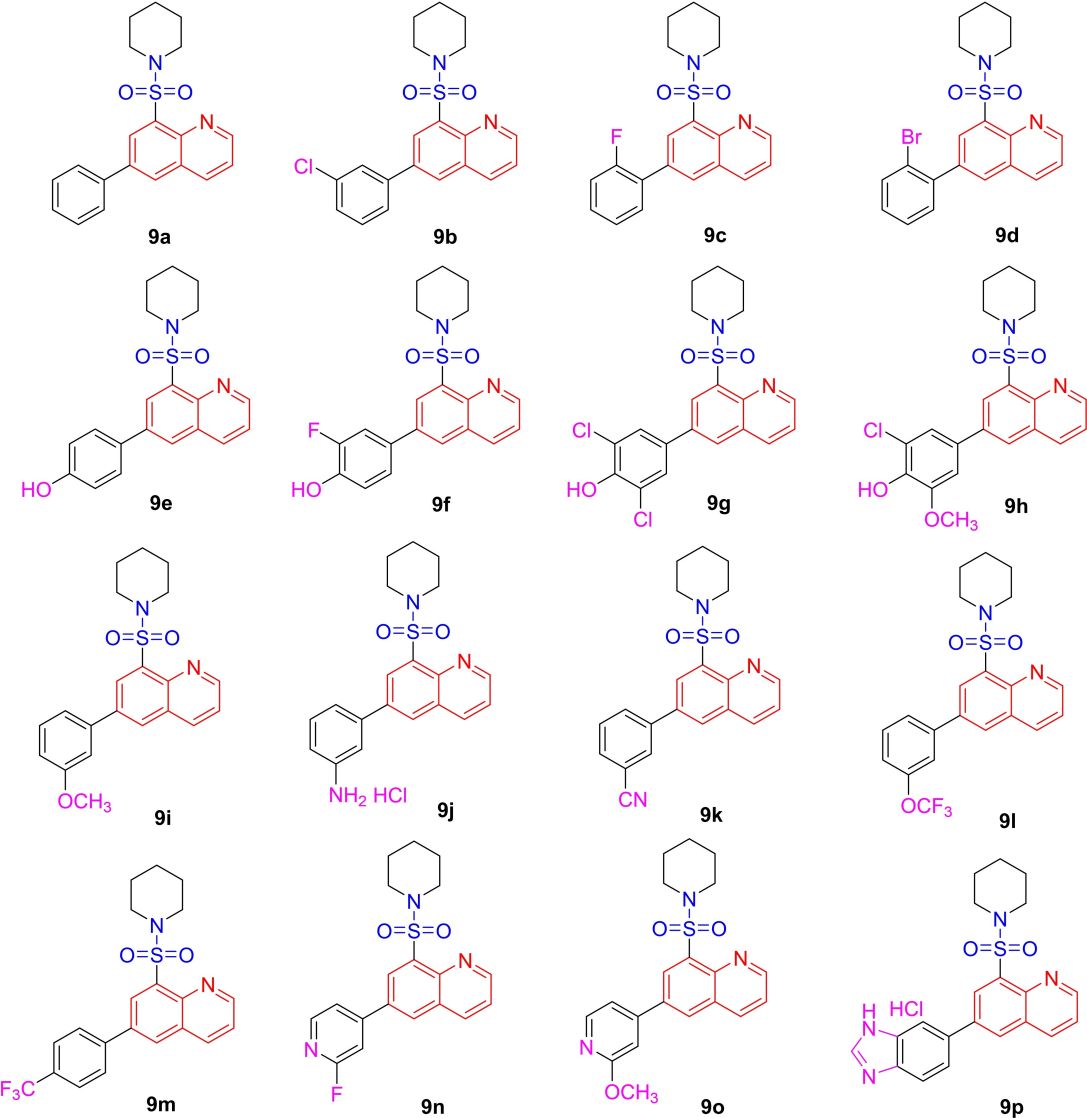
Library of synthesized quinoline and sulfonamide molecular hybrids.

**Figure 4:**
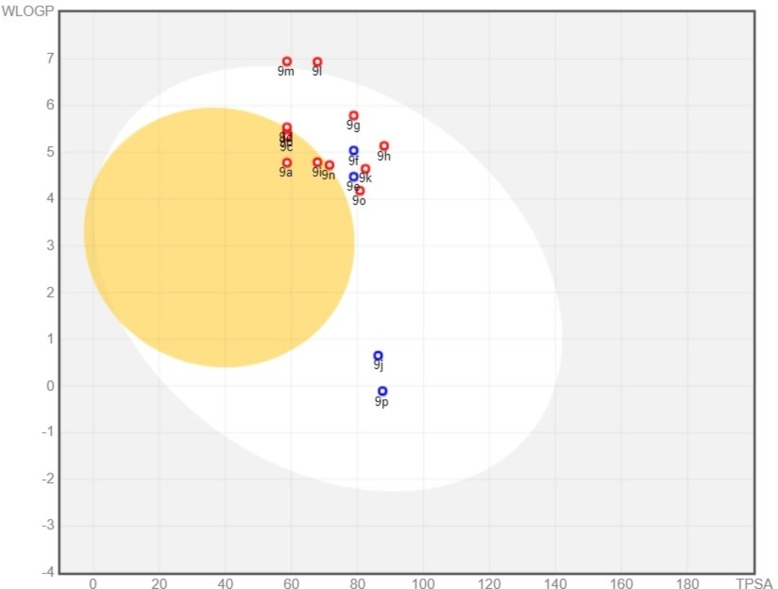
BOILEDEgg model for molecular hybrids of quinoline and sulfonamide.

Anticipated pharmacokinetic behaviors, including gastrointestinal absorption (GIA) and blood‐brain barrier (BBB) permeation, were investigated. Employing the Brain Or Intestinal EstimateD Permeation method (BOILEDEgg), a graphical representation of lipophilicity (WLOGP) against topological polar surface area (TPSA) has been provided. This analysis suggests that compounds **9 a**, **9 b**, **9 c**, **9 d**, **9 i**, **9 n**, falling within the yellow ellipse, are likely to exhibit superior BBB permeation properties. Conversely, compounds **9 l** and **9 m**, positioned in the gray region, are expected to have a reduced likelihood of passing through the BBB or the gastrointestinal barrier (GIA). All other compounds fall within the white ellipse region, indicative of potential favorable GIA. Central Nervous System (CNS) efflux activity, predictions using P‐glycoprotein (PGP) indicate that compounds **9 e**, and **9 f**, show positive results, implying potential interactions with PGP. In contrast, the remaining compounds exhibit negative PGP results. These outcomes are concisely presented in Table 1 and Figure 4.

### 2.2 Chemistry

**Scheme 1:**
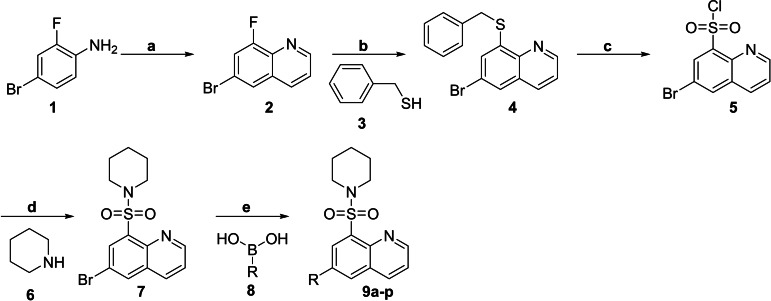
Reaction conditions: (a) glycerol, H_2_SO_4_, H_2_O, 200 °C, 0.5 h, 65 %; (b) NaH, THF, 25 °C, 3 h, 79 %; (c) NCS, AcOH/H_2_O, 25 °C, 5 h, 87 %; (d) DIPEA, THF, 25 °C, 2 h, 90 %; (e) Pd(dppf)Cl_2_, Cs_2_CO_3_, 1,4‐dioxane, MW, 125 °C, 0.5 h, 60 %.

The synthesis pathway for the molecular hybrids of quinoline and sulfonamide framework was successfully executed following a sequence of reactions outlined in Scheme 1. Employing the established procedure, the Skraup synthesis of commercially available 4‐Bromo‐2‐fluoroaniline, **1** with glycerol under acidic conditions yielded the quinoline scaffold **2**.^[26]^ Subsequent nucleophilic substitution of the latter with benzyl mercaptan **3** led to the formation of the 8‐thioquinoline intermediate, **4**. Employing NCS in an AcOH/H_2_O mixture, the intermediate compound **4** underwent oxyhalogenation to produce the quinoline sulfonyl chloride compound **5**. This compound was then subjected to nucleophilic substitution with piperidine, resulting in the formation of the quinoline sulfonamide intermediate **7**. The final compounds, denoted as **9 a‐p**, were successfully synthesized in satisfactory yields *via* Suzuki coupling of intermediate **7** with diverse aryl boronic acids. Notably, these reactions were conducted using microwave conditions in dioxane. The recently synthesized compounds **9 a‐p** underwent thorough characterization through the utilization of NMR (^1^H, ^13^C) and HRMS data. A detailed account of these findings is included in the experimental section.

### 2.3 In vitro Anti‐cancer Studies

The anti‐cancer activities of novel hybrid molecules combining quinoline and sulfonamide (designated as **9 a** to **9 p**) were investigated in vitro. These hybrids were tested against various cancer cell lines including HCT116, A549, U2OS, CCRF‐CEM, Jurkat, MOLT‐4, K562 and RAMOS. Non‐cancer cell lines, BJ and MRC‐5 were also included for comparison. The outcomes are summarized in Table 3. When examining the effects on A549, HCT116, and U2OS cells, all tested compounds exhibited limited potency with IC_50_ values exceeding 50 μM, indicating weak activity against these cell lines. Against the ITK high cells *Viz*. are Jurkat, CCRF‐CEM and MOLT‐4, **9 e, 9 p** and **9 j** found to the most active molecules with IC_50_ values of 7.43±7.40 μM, 13.19±1.25 μM and 5.57±7.56 μM respectively. In addition to these, other molecules which are found potent against the Jurkat cells includes, **9 i**, **9 m, 9 o, 9 l**, **9 c**, **9 f**, **9 d**, and **9 p** with IC_50_ values of 8.43±6.43 μM, 11.32±1.13 μM, 11.41±3.49 μM, 12.13±6.43 μM, 12.65±5.45 μM, 13.04±6.43 μM, 13.45±6.25 μM, and 21.21±3.49 μM respectively. Against CCRF‐CEM cells, other potent molecules include **9 o**, **9 f**, **9 n**, **9 e** and **9 i** with IC_50_ values of 15.29±2.25 μM, 17.09±4.64 μM, 19.39±0.78 μM, 28.59±7.64 μM and 38.59±4.64 μM respectively. Similarly, in the BTK high cells screenings, **9 n** and **9 e** molecules found to be most active by IC_50_ values 2.76±0.79 μM and 5.47±1.71 μM against RAMOS and K562 respectively. The other potent molecules against RAMOS are **9 e**, **9 o**, **9 m**, **9 f** and **9 d** with IC_50_ values of 5.47±1.71 μM, 6.76±0.79 μM, 8.11±1.29 μM, 9.69±2.41 μM and 12.7±3.71 μM respectively. The molecule **9 f** is the other potent molecule with IC_50_ value of 9.69±2.41 μM against K562 cells. Interestingly, all the molecules have exhibited IC_50_ value >50 μM against the non‐cancer cells (MRC‐5 and BJ), which indicates the promising non‐cytotoxic nature of the molecules.

**Table 3:** *in vitro* anti‐cancer studies of quinoline and sulfonamide molecular hybrids.

Code Name	ITK/BTK null (μM)			ITK high cells (μM)			BTK high cells (μM)		Non‐cancer cells (μM)	
	A549	HCT116	U2OS	Jurkat	CCRF‐CEM	MOLT‐4	RAMOS	K562	MRC‐5	BJ
9a	>50	>50	>50	>50	>50	>50	>50	>50	>50	>50
9b	>50	>50	>50	>50	>50	>50	>50	>50	>50	>50
9c	>50	>50	>50	12.65±5.45	>50	>50	>50	>50	>50	>50
9d	>50	>50	>50	13.45±6.25	>50	>50	12.7±3.71	>50	>50	>50
9e	>50	>50	>50	7.43±7.40	28.59±7.64	>50	5.47±1.71	5.47±1.71	>50	>50
9f	>50	>50	>50	13.04±6.43	17.09±4.64	>50	9.69±2.41	9.69±2.41	>50	>50
9g	>50	>50	>50	>50	>50	>50	>50	>50	>50	>50
9h	>50	>50	>50	>50	>50	>50	>50	>50	>50	>50
9i	>50	>50	>50	8.43±6.43	38.59±4.64	>50	>50	>50	>50	>50
9j	>50	>50	>50	>50	>50	5.57±7.56	>50	>50	>50	>50
9k	>50	>50	>50	>50	>50	>50	>50	>50	>50	>50
9l	>50	>50	>50	12.13±6.43	>50	>50	>50	>50	>50	>50
9m	>50	>50	>50	11.32±1.13	>50	>50	8.11±1.29	>50	>50	>50
9n	>50	>50	>50	>50	19.39±0.78	>50	2.76±0.79	>50	>50	>50
9o	>50	>50	>50	11.41±3.49	15.29±2.25	>50	6.76±0.79	>50	>50	>50
9p	>50	>50	>50	21.21±3.49	13.19±1.25	>50	>50	>50	>50	>50

## Experimental Section

### Chemistry

Thin‐layer chromatography (TLC) was employed to track the advancement of the reactions, all of which were conducted in glassware that had been dried in an oven. The melting points were determined using a VEEGO VMP‐DS melting point instrument, and measured in open‐end capillaries without any adjustments made. The Nicolet‐6700 spectrometer with KBr was used to record the infrared (IR) spectra. The Bruker Advance 400 spectrometer was used to acquire proton (1H) and carbon‐13 (13 C) NMR spectra. The solvents used were DMSO‐d6 or CDCl_3_. Chemical shift values (δ) are expressed in parts per million (ppm) with respect to tetramethylsilane (SiMe_4_, δ=0.00), which serves as the internal reference. The values of coupling constants (J) are expressed in units of hertz (Hz). The description of multiplicities involves the use of specific terminology such as singlet (s), doublet (d), triplet (t), quartet (q), multiplet (m), and wide (br).

Synthesis of 6‐bromo‐8‐fluoroquinoline (**2**): Aniline derivative 1 (5.29 mmol), glycerol (15.8 mmol, 3.0 equivalents), and H_2_SO_4_ (15.8 mmol, 3 equivalents) were added to 20 mL of water in a 50 mL sealed vessel. The mixture was heated to 100 °C for 10 minutes at a rate of 36 °C per minute under sufficient radiation to reach the desired temperature. NaOH was used to alter the pH to 8–9 after cooling to room temperature. Three times twenty milliliters of ethyl acetate were added to the reaction mixture for extraction. After being dried over MgSO_4_, filtered, and concentrated under decreased pressure, the mixed organic layers were thoroughly processed. The resulting crude residue was purified by column chromatography on silica gel using a cyclohexane–ethyl acetate solvent system, yielding the desired 6‐bromo‐8‐fluoroquinoline.

*Synthesis of 8‐(benzylthio)‐6‐bromoquinoline (**4**)*: After placing 5 mmol of 6‐bromo‐8‐fluoroquinoline into a two‐neck round‐bottom flask, the flask was evacuated and backfilled with argon three times. Then, 15 mL of THF was injected using a syringe. 6 millimoles of phenylmethanethiol were added to the flask, which was thereafter placed in an ice bath and chilled to a temperature of 0 °C. Sodium hydride (7.5 mmol) was added gradually over a period of 5 minutes with stirring at a temperature of 0 °C. The reaction was halted by introducing a concentrated solution of NH_4_Cl in water at 0 °C, following agitation at either 0 °C or 25 °C until the completion of the reaction was confirmed by TLC monitoring. The mixture was passed to a separatory funnel, and the organic extracts were pooled, rinsed with brine, dehydrated using Na_2_SO_4_, filtered, and concentrated under reduced pressure. The unrefined residue underwent purification using column chromatography using silica gel, yielding the desired product.

*Synthesis of 6‐bromoquinoline‐8‐sulfonyl chloride (**5**)*: In an ice bath, add 10 mmol of NCS immediately to a solution of 8‐(benzylthio)‐6‐bromoquinoline (3 mmol) in an acetic acid‐water mixture (3 : 1). Agitate the compound at a temperature range of 5–10 °C for a duration of 50 minutes, followed by allowing it to gradually increase in temperature to 25–30 °C in order to finalize the reaction. Thoroughly dilute the combination by adding 60 mL of CCl_4_, cleanse it with cold water, remove any moisture by using MgSO_4_, and decrease the volume by applying lower pressure. Recrystallize the crude product from hexane to obtain the pure product 5.

*Synthesis of 6‐bromo‐8‐(piperidin‐1‐ylsulfonyl)quinoline (**7**)*: At room temperature, amine (2.24 mmol) and of DIPEA (2.24 mmol) were added dropwise to a stirred solution of 6‐bromo‐8‐(piperidin‐1‐ylsulfonyl)quinoline (2.2 mmol) in THF. The reaction was allowed to continue until it reached completion, as measured by thin‐layer chromatography (TLC). Subsequently, the reaction mixture was subjected to extraction using ethyl acetate. The pure substance was acquired through the process of refining the mixture resulting from the reaction. This was achieved by employing column chromatography, specifically utilizing a combination of ethyl acetate and hexane as the solvent, and silica gel with a particle size of 60–120 mesh as the stationary phase. The obtained substance was subsequently dehydrated using anhydrous sodium sulfate and concentrated by reducing the pressure.

*General procedure for the synthesis of 6‐phenyl‐8‐(piperidin‐1‐ylsulfonyl)quinolone derivatives (**9 a‐9 p**)*: 6‐Bromo‐8‐(piperidin‐1‐ylsulfonyl)quinoline (2.0 mmol) was dissolved in 10 mL of 1,4‐dioxane, and the solution was purged with argon to remove air. Cs_2_CO_3_ (6.0 mmol), phenylboronic acid (2.0 mmol), and 0.5 mol % Pd(dppf)Cl_2_ (0.02 mmol) were then added, and the mixture was stirred under an argon atmosphere. The combination underwent heating to a temperature of 125 °C using a microwave reactor for a duration of 30 minutes. Once finished, the mixture was cooled to the temperature of the surrounding environment and then moved to a separatory funnel. Additional dichloromethane was used to extract the aqueous layer. The organic extracts were mixed and dehydrated using MgSO_4_. The solvent was then evaporated under reduced pressure, resulting in the crude product. The raw substance underwent purification using flash chromatography using a silica gel column.

*6‐phenyl‐8‐(piperidin‐1‐ylsulfonyl)quinoline (**9 a**)*: ^1^H NMR (400 MHz, DMSO): *δ*=9.08 (dd, 1H), 8.76 (d, 1H), 8.28 (dd, 1H), 8.18 (d, 1H), 7.76–7.72 (m, 2H), 7.56–7.50 (m, 3H), 7.47–7.35 (m, 1H), 3.42 (t, 4H), 1.66–1.58 (m, 4H), 1.50–1.45 (m, 2H); ESI‐MS (M+H)^+^: 353.33.

*6‐(3‐chlorophenyl)‐8‐(piperidin‐1‐ylsulfonyl)quinoline (**9 b**)*: ^1^H NMR (400 MHz, DMSO): *δ*=9.07 (dd, 1H), 8.67 (d, 1H), 8.60–8.55 (m, 2H), 7.96 (t, 1H), 7.85–7.81 (m, 1H), 7.75–7.71 (m, 1H), 7.63–7.53 (m, 1H), 3.35–3.28 (m, 4H), 1.52–1.46 (m, 4H), 1.46–1.40 (m, 2H). ^13^C NMR (100 MHz, DMSO): δ=151.6, 142.8, 140.2, 137.1, 137.0, 135.4, 134.0, 131.4, 131.13, 131.0, 129.1, 128.3, 126.9, 125.9, 122.8, 46.6, 25.4, 23.2; ESI‐MS (M+H)^+^: 387.65.

*6‐(2‐fluorophenyl)‐8‐(piperidin‐1‐ylsulfonyl)quinoline (**9 c**)*: ^1^H NMR (400 MHz, DMSO): *δ*=9.10 (d, 1H), 8.66 (s, 1H), 8.27 (d, 1H), 8.19 (s, 1H), 7.60–7.52 (m, 2H), 7.48–7.38 (m, 1H), 7.31–7.27 (m, 1H), 7.2.0–7.22 (m, 1H), 3.42 (t, 4H), 1.66–1.56 (m, 4H), 1.51–1.44 (m, 2H). ESI‐MS (M+H)^+^: 371.52.

*6‐(2‐bromophenyl)‐8‐(piperidin‐1‐ylsulfonyl)quinoline (**9 d**)*: ^1^H NMR (400 MHz, DMSO): *δ*=9.10 (dd, 1H), 8.58 (dd, 1H), 8.38–8.32 (m, 2H), 7.84 (dd, 1H), 7.76–7.71 (m, 1H), 7.64–7.60 (m, 1H), 7.59–7.53 (m, 1H), 7.46–7.41 (m, 1H), 3.36–3.28 (m, 4H), 1.52–1.46 (m, 4H), 1.43–1.38 (m, 2H).13 C NMR (100 MHz, DMSO): δ=151.7, 137.0, 135.8, 133.6, 133.1, 132.6, 131.7, 130.3, 128.6, 128.3, 122.7, 121.7, 46.6, 25.4, 23.1; ESI‐MS (M+H)^+^: 433.29.

*4‐(8‐(piperidin‐1‐ylsulfonyl)quinolin‐6‐yl)phenol (**9 e**)*: ^1^H NMR (400 MHz, DMSO): *δ*=9.02 (s, 1H), 8.71 (d, 1H), 8.23 (d, 1H), 8.10 (d, 1H), 7.62 (d, 2H), 7.50 (dd, 1H), 6.99 (d, 2H), 5.38 (s, 1H), 3.42 (t, 4H), 1.63–1.58 (m, 4H), 1.50–1.46 (m, 2H). ESI‐MS (M+H)^+^: 369.43.

*2‐fluoro‐4‐(8‐(piperidin‐1‐ylsulfonyl)quinolin‐6‐yl)phenol (**9 f**)*: ^1^H NMR (400 MHz, DMSO): *δ*=10.24 (s, 1H), 9.02 (dd, 1H), 8.55–8.50 (m, 3H), 7.75–7.65 (m, 2H), 7.51 (dd, 1H), 7.12 (t, 1H), 3.28 (t, 4H), 1.55–1.48 (m, 4H), 1.41–1.39 (m, 2H); ESI‐MS (M+H)^+^: 387.45

*2,6‐dichloro‐4‐(8‐(piperidin‐1‐ylsulfonyl)quinolin‐6‐yl)phenol (**9 g**)*: ^1^H NMR (400 MHz, DMSO): *δ*=10.53 (s, 1H), 9.04 (dd, 1H), 8.61 (d, 1H), 8.53–8.51 (m, 2H), 7.90 (s, 2H), 7.71–7.68 (m, 1H), 3.29 (t, 4H), 1.54–1.46 (m, 4H), 1.42–1.38 (m, 2H).^13^C NMR (100 MHz, DMSO): δ=151.3, 149.3, 142.6, 136.99, 136.91, 134.4, 130.9, 130.8, 130.6, 129.0, 127.1, 123.0, 122.8, 46.6, 25.4, 23.2; ESI‐MS (M+H)^+^: 436.04.

*2‐chloro‐6‐methoxy‐4‐(8‐(piperidin‐1‐ylsulfonyl)quinolin‐6‐yl)phenol (**9 h**)*: ^1^H NMR (400 MHz, DMSO): *δ*=9.79 (s, 1H), 9.03 (dd, 1H), 8.57 (d, 1H), 8.54–8.50 (m, 2H), 7.72–7.68 (m, 1H), 7.20 (ABq, 2H), 3.97(s, 3H), 3.29 (t, 4H), 1.55–1.48 (m, 4H), 1.43–1.39 (m, 2H); ESI‐MS (M+H)^+^: 433.51.

*6‐(3‐methoxyphenyl)‐8‐(piperidin‐1‐ylsulfonyl)quinoline (**9 i**)*: ^1^H NMR (400 MHz, DMSO): *δ*=9.06 (dd, 1H), 8.60 (d, 1H), 8.57–8.55 (m, 2H), 7.73–7.69(m, 1H), 7.50–7.46 (m, 1H), 7.41–7.38 (m, 2H), 7.05(dd, 1H), 3.87 (s, 3H), 3.30 (t, 4H), 1.55–1.49 (m, 4H), 1.41–1.39 (m, 2H); ESI‐MS (M+H)^+^: 383.41.

*3‐(8‐(piperidin‐1‐ylsulfonyl)quinolin‐6‐yl)aniline hydrochloride (**9 j**)*: ^1^H NMR (400 MHz, DMSO): *δ*=9.09 (dd, 1H), 8.64–8.59 (m, 2H), 8.55 (d, 1H), 7.88 (d, 1H), 7.79 (s, 1H), 7.77–7.72 (m, 1H), 7.67 (t, 1H), 7.45 (d, 1H), 3.32–3.27 (m, 4H), 1.51–1.41 (m, 6H). 21^13^C NMR (100 MHz, DMSO): δ=151.6, 139.4, 137.2, 135.7, 131.1, 130.8, 130.7, 129.1, 122.9, 122.6 121.2, 46.6, 25.4, 23.2; ESI‐MS (M+H)^+^: 403.11.

*3‐(8‐(piperidin‐1‐ylsulfonyl)quinolin‐6‐yl)benzonitrile (**9 k**)*: ^1^H NMR (400 MHz, DMSO): *δ*=9.09 (dd, 1H), 8.71 (d, 1H), 8.60 (d, 1H), 8.59–8.55 (m, 1H), 8.42 (s, 1H), 8.22 (d, 1H), 7.94 (d, 1H), 7.79–7.73 (m, 2H), 3.30 (t, 4H), 1.51–1.41 (m, 6H). ^13^C NMR (100 MHz, DMSO): δ=151.7, 142.9, 139.2, 137.2, 134.9, 132.0, 131.9, 131.7, 131.1, 130.9, 130.4, 129.0, 127.1, 122.9, 118.5, 112.3, 46.6, 25.4, 23.2; ESI‐MS (M+H)^+^: 377.12.

*8‐(piperidin‐1‐ylsulfonyl)‐6‐(3‐(trifluoromethoxy)phenyl)quinolone (**9 l**)*: ^1^H NMR (400 MHz, DMSO): *δ*=9.08 (dd, 1H), 8.68 (d, 1H), 8.60–8.56 (m, 2H), 7.94–7.88 (m, 2H), 7.76–7.68 (m, 2H), 7.50 (d, 1H), 3.34–3.28 (t, 4H), 1.54–1.47 (m, 4H), 1.44–1.38 (m, 2H). ^13^C NMR (100 MHz, DMSO): δ=151.6, 149.1, 142.9, 140.5, 137.2, 137.1, 135.3, 131.5, 131.2, 131.0, 129.0, 126.4, 122.9, 121.4, 120.7, 119.9, 118.8, 46.6, 25.4, 23.2; ESI‐MS (M+H)^+^: 436.45.

*8‐(piperidin‐1‐ylsulfonyl)‐6‐(4‐(trifluoromethyl)phenyl)quinoline (**9 m**)*: ^1^H NMR (400 MHz, DMSO): *δ*=9.01 (dd, 1H), 8.71 (d, 1H), 8.63–8.58 (m, 2H), 8.10 (d, 2H), 7.93 (d, 2H), 7.77–7.72 (m, 1H), 3.33–3.28)m, 4H), 1.51–1.48 (m, 4H), 1.44–1.39 (m, 2H); ESI‐MS (M+H)^+^: 421.39.

*6‐(2‐fluoropyridin‐4‐yl)‐8‐(piperidin‐1‐ylsulfonyl)quinoline (**9 n**)*: ^1^H NMR (400 MHz, DMSO): *δ*=9.08 (dd, 1H), 8.75 (d, 1H), 8.66 (d, 1H), 8.57–8.55 (m, 2H), 8.52–8.47 (m, 1H), 7.75–7.72 (m, 1H), 7.38 (dd, 1H), 3.32–3.29 (m, 4H), 1.49–1.41 (m, 6H). ^13^C NMR (100 MHz, DMSO): δ=164.1, 161.8, 151.6, 146.2, 146.1, 142.8, 141.1, 141.0, 137.2, 137.0, 133.0, 132.5, 132.4, 131.5, 131.0, 129.0, 122.9, 110.1, 109.7, 46.6, 25.4, 23.2; ESI‐MS (M+H)^+^: 371.11.

*6‐(2‐methoxypyridin‐4‐yl)‐8‐(piperidin‐1‐ylsulfonyl)quinoline (**9 o**)*: ^1^H NMR (400 MHz, DMSO): *δ*=9.10 (dd, 1H), 8.75 (d, 1H), 8.60–8.57 (m, 2H), 8.33 (d, 1H), 7.76–7.73(m, 1H), 7.48 (d, 1H), 7.30 (s, 1H), 3.93 (s, 3H), 3.30 (t, 4H), 1.55–1.49 (m, 4H), 1.41–1.39 (m, 2H); ESI‐MS (M+H)^+^: 384.32.

*6‐(1H‐benzo[d]imidazol‐6‐yl)‐8‐(piperidin‐1‐ylsulfonyl)quinoline hydrochloride (**9 p**)*: ^1^H NMR (400 MHz, DMSO): *δ*=9.10 (dd, 1H), 8.71 (d, 1H), 8.66–8.62 (m, 2H), 8.27 (s, 23H), 8.10–8.02 (m, 2H), 7.77–7.73 (m, 1H), 3.33–3.30 (m, 4H), 1.55–1.49 (m, 4H), 1.47–1.40 (m, 2H). ^13^C NMR (100 MHz, DMSO): δ=151.6, 142.7, 141.5, 137.2, 137.1, 136.3, 136.2, 131.7, 131.49, 131.41, 130.6, 129.2, 125.7, 122.9, 115.3, 113.0; ESI‐MS (M+H)^+^: 436.04.

### In Vitro Assay for Evaluation of Anti‐Cancer Activity

Cell lines: The cells were sourced from either the DSMZ (Braunschweig, Germany) or the ATCC (Middlesex, UK). They were maintained in the recommended growth medium, supplemented with 10 % fetal calf serum, antibiotics (100 U/mL penicillin and 100 mg/mL streptomycin), 2 mM glutamine, 1 mM NaHCO_3_, 1 mM sodium pyruvate, and 20 mM HEPES. The cells were incubated at 37 °C in a humidified atmosphere with 5 % CO_2_. Cell lines were routinely verified and checked for mycoplasma contamination every two weeks to once a month.

MTS cytotoxicity testing: An MTS assay method was developed in our laboratory for routine drug screening. This technique was used to assess the cytotoxicity of compounds, and the IC_50_ values were determined as previously described.^[27]^

## 3 Conclusions

In summary, diversely functionalized molecular hybrids of quinoline and sulfonamide were designed. multistep synthetic strategies used for the synthesis. The analyses of the anti‐cancer properties of the synthesised molecules evaluated against various cancer cell lines including HCT116, U2OS, A549, CCRF‐CEM, Jurkat, MOLT‐4, K562 and RAMOS. Non‐cancer cell lines BJ and MRC‐5 were also included for comparison. When examining the effects on A549, HCT116, and U2OS cells, all tested compounds exhibited limited potency with IC_50_ values exceeding 50 μM, indicating weak activity against these cell lines. Against the ITK high cells *Viz*. are Jurkat, CCRF‐CEM and MOLT‐4, **9 e, 9 p** and **9 j** molecules identified as most active with an IC_50_ value of 7.43±7.40 μM, 13.19±1.25 μM and 5.57±7.56 μM respectively. Similarly, in the BTK high cells screenings, **9 n** and **9 e** molecules identified as most active with an IC_50_ value of 2.76±0.79 μM and 5.47±1.71 μM against RAMOS and K562 respectively. Interestingly, all the molecules have exhibited IC_50_ value >50 against the non‐cancer cells (MRC‐5 and BJ), which indicates the promising non‐cytotoxic nature of the molecules.

## Ethics Approval

This article does not contain any studies with animals performed by any of the authors.

## Author Contributions

P.P: Investigation, Formal Analysis, Methodology, and Writing ‐ Original Draft. P.M: Software, and Writing ‐ Original Draft. N.K.K: Data Curation, and Writing – review & editing. R.G: Conceptualisation, and Project Administration. S.B.J: Resources, and Writing – review & editing. B.K.T: Visualization, Validation and Supervision.

## Conflict of Interests

The authors declare that they have no competing interests/competing interests.

4

## Supporting information

As a service to our authors and readers, this journal provides supporting information supplied by the authors. Such materials are peer reviewed and may be re‐organized for online delivery, but are not copy‐edited or typeset. Technical support issues arising from supporting information (other than missing files) should be addressed to the authors.

Supporting Information

## Data Availability

All data generated or analyzed during this study are included in this published article and its supplementary information files. (Confirmed that the correct version appears)
