# Supplementary material for: Molecular Hybrids of Quinoline and Sulfonamide: Design, Synthesis and in Vitro Anticancer Studies
Source: ChemistryOpen. 2024 Nov 26;14(3):e202400334. doi: 10.1002/open.202400334 (PMC12128149; doi:10.1002/open.202400334)
Supplement: Supplementary file 1 — Supporting Information [file OPEN-14-e202400334-s001.pdf]

ChemistryOpen

Supporting Information

Molecular Hybrids of Quinoline and Sulfonamide: Design, Synthesis and *in Vitro* Anticancer Studies

Padyala Panduranga, Parameshwar Makam, Naresh Kumar Katari,* Rambabu Gundla, Sreekantha Babu Jonnalagadda, and Bharat Kumar Tripuramallu*

Supporting Information

Molecular hybrids of quinoline and sulfonamide: Design, synthesis and *in vitro* anticancer studies

Padyala Panduranga[1], Parameshwar Makam[2], Naresh Kumar Katari*[4], Rambabu Gundla[3], Sreekantha Babu Jonnalagadda[4], Bharat Kumar Tripuramallu*[1]

[1]Department of Chemistry, VFSTR (Deemed to be University), Vadlamudi, 522213 Guntur, Andhra Pradesh, India.

[2]Division of Research and Innovation, Department of Chemistry, Uttaranchal University, Arcadia Grant, P.O. Chandanwari, Premnagar, Dehradun, Uttarakhand-248007, India.

[3]Department of Chemistry, GITAM School of Science, GITAM Deemed to be University, Hyderabad, Telangana, India 502329.

[4]School of Chemistry & Physics, College of Agriculture, Engineering & Science, Westville Campus, University of KwaZulu-Natal, P Bag X 54001, Durban-4000, South Africa.

*Corresponding author: aryanbharat@gmail.com; KatariN@ukzn.ac.za

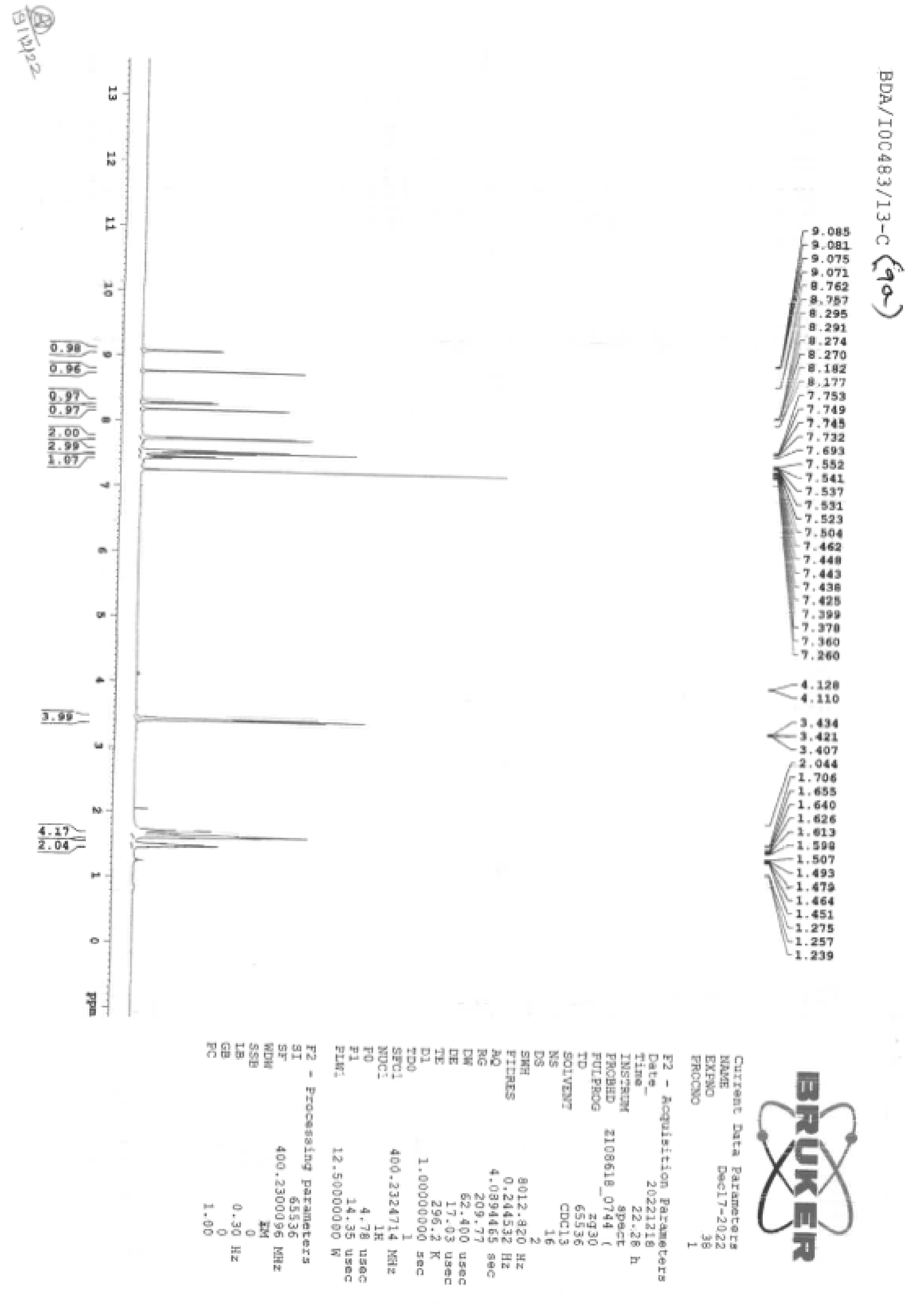

^{1}H-NMR spectrum of compound 9a

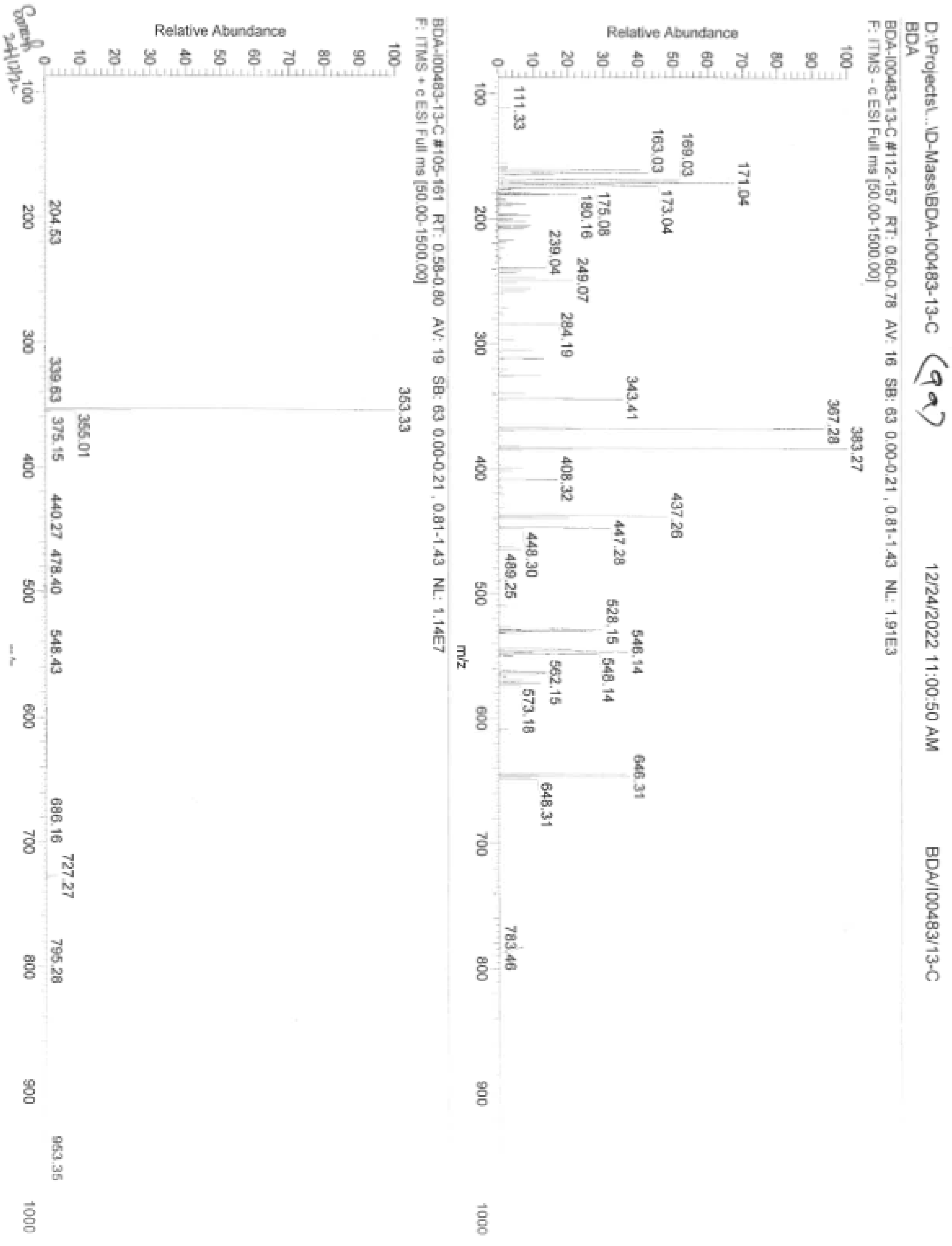

Mass spectrum of compound 9a

MICRO LABS LIMITED
API, R & D CENTRE, BOMMASANDRA (ML-27)
ANALYTICAL RESEARCH DIVISION

Sample Name : BDA-4
Sample ID : BDA/I00483/13C (9a)
Instrument ID : ARD_HLC_213
Test Parameter : RS (M1)
Column ID : ARD/LCC/21/032
Sample Set Name : 21322L012
Run Time : 75.0 Minutes
Column Details : Bakerbond Q2 100 C18 (150 mm * 4.6 mm , 3.0µm)
Project Name : DEC_2022\ARD_HLC_213

Channel Description UV Detector: Channel 1, 225 nm
Aqu Method Set : BDA 4 RS M1
Processing Method : BDA_4_RS_M1_SPL
Vial No. : 5
Injection Volume : 5 uL
Analyst : Sivanand.M

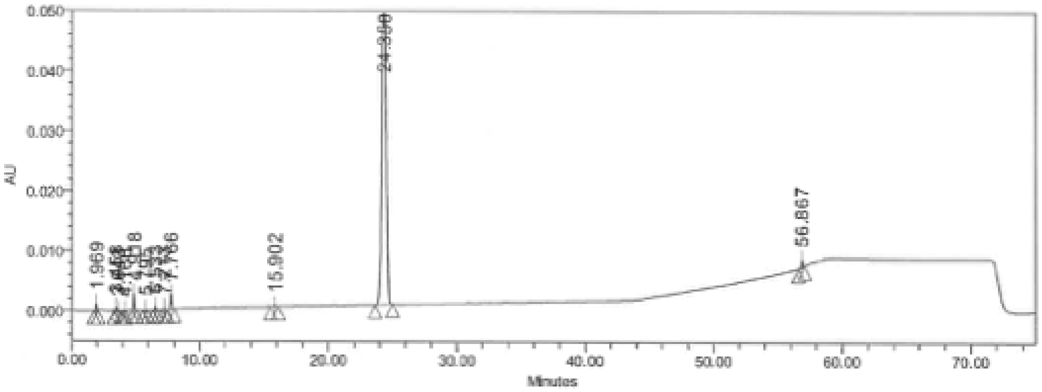

Sample Name BDA-4; Injection 1; Date Acquired 23-12-2022 17:11:46 IST; Date Processed 24-12-2022 10:41:08 IST; Result #

	Peak Name	RT	Height (µV)	Area	Int Type	RT Ratio	% Area	RRF	Corrected %Area
1	Peak1	1.97	1215	6322	BB	0.08	0.454	1.00	0.45
2	Peak2	3.46	640	3753	BV	0.14	0.270	1.00	0.27
3	Peak3	3.64	155	1142	VB	0.15	0.082	1.00	0.08
4	Peak4	4.17	79	576	BB	0.17	0.041	1.00	0.04
5	Peak5	4.92	2742	17848	BB	0.20	1.283	1.00	1.28
6	Peak6	5.80	166	1188	BB	0.24	0.085	1.00	0.09
7	Peak7	6.53	555	4491	BB	0.27	0.323	1.00	0.32
8	Peak8	7.21	304	2436	BB	0.30	0.175	1.00	0.18
9	Peak9	7.77	2564	22249	BB	0.32	1.599	1.00	1.60

Processed By sivanandm/Analyst

HPLC of compound 9a

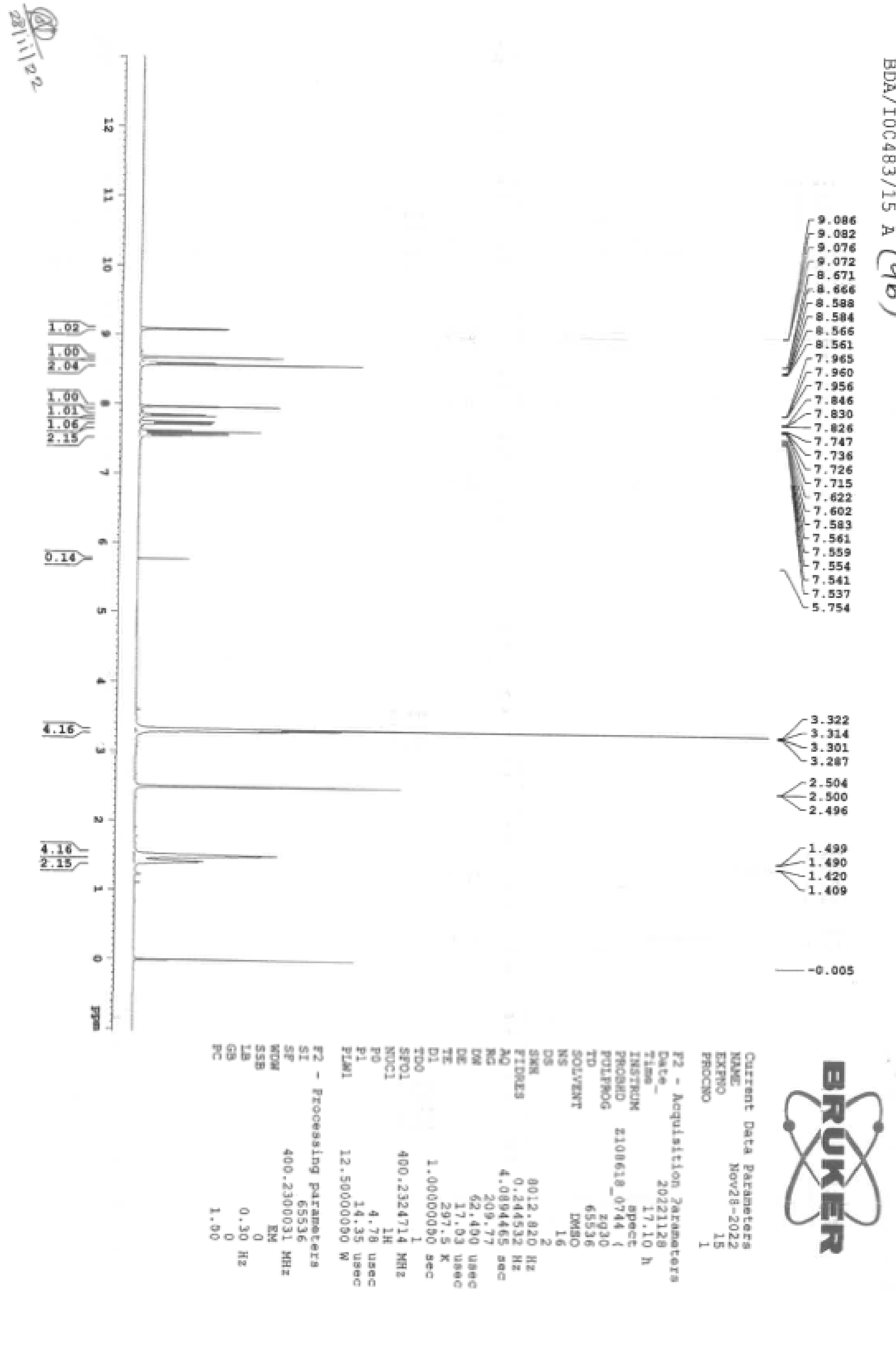

^{1}H-NMR spectrum of compound 9b

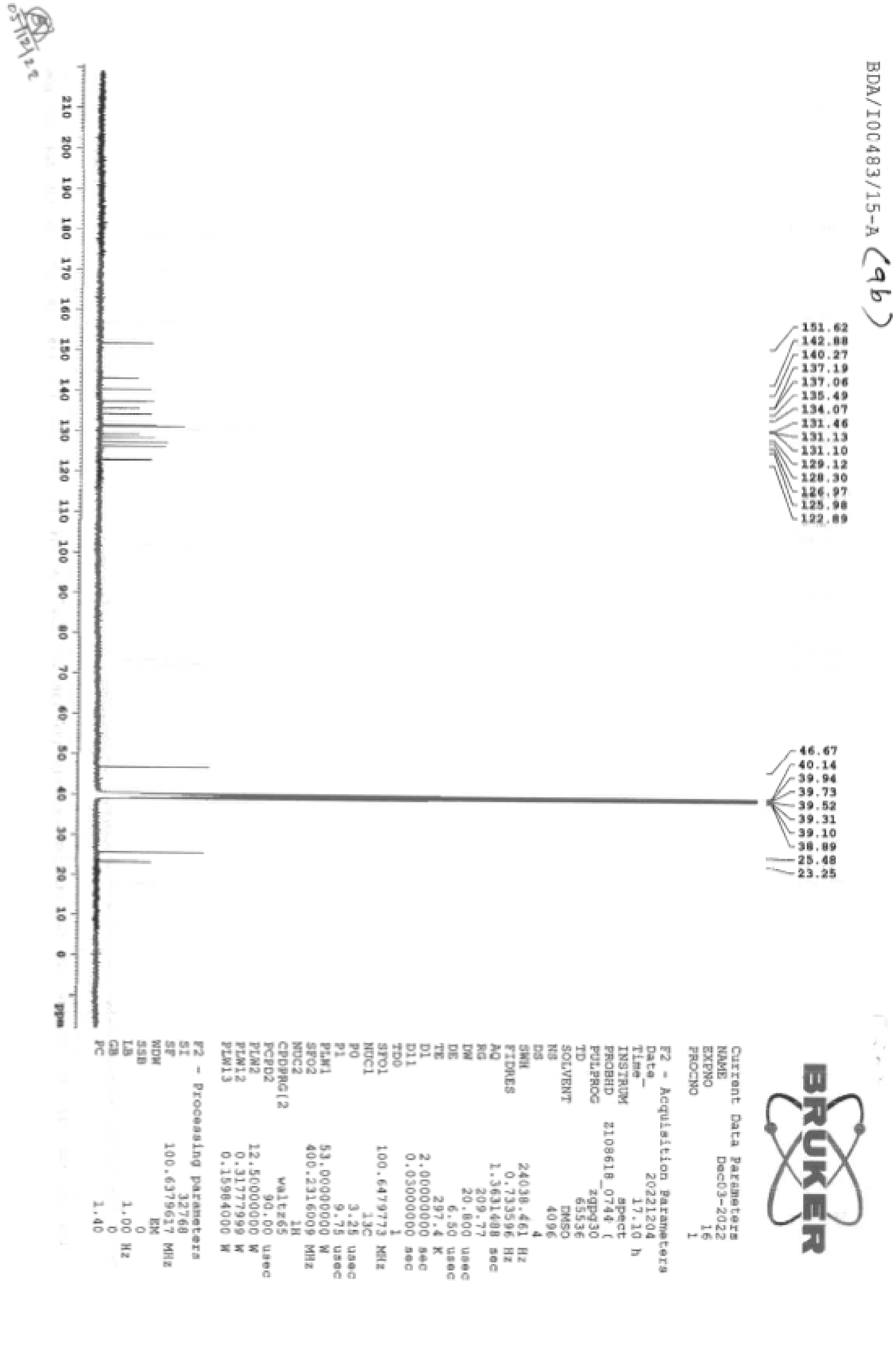

^{13}C NMR spectrum of compound 9b

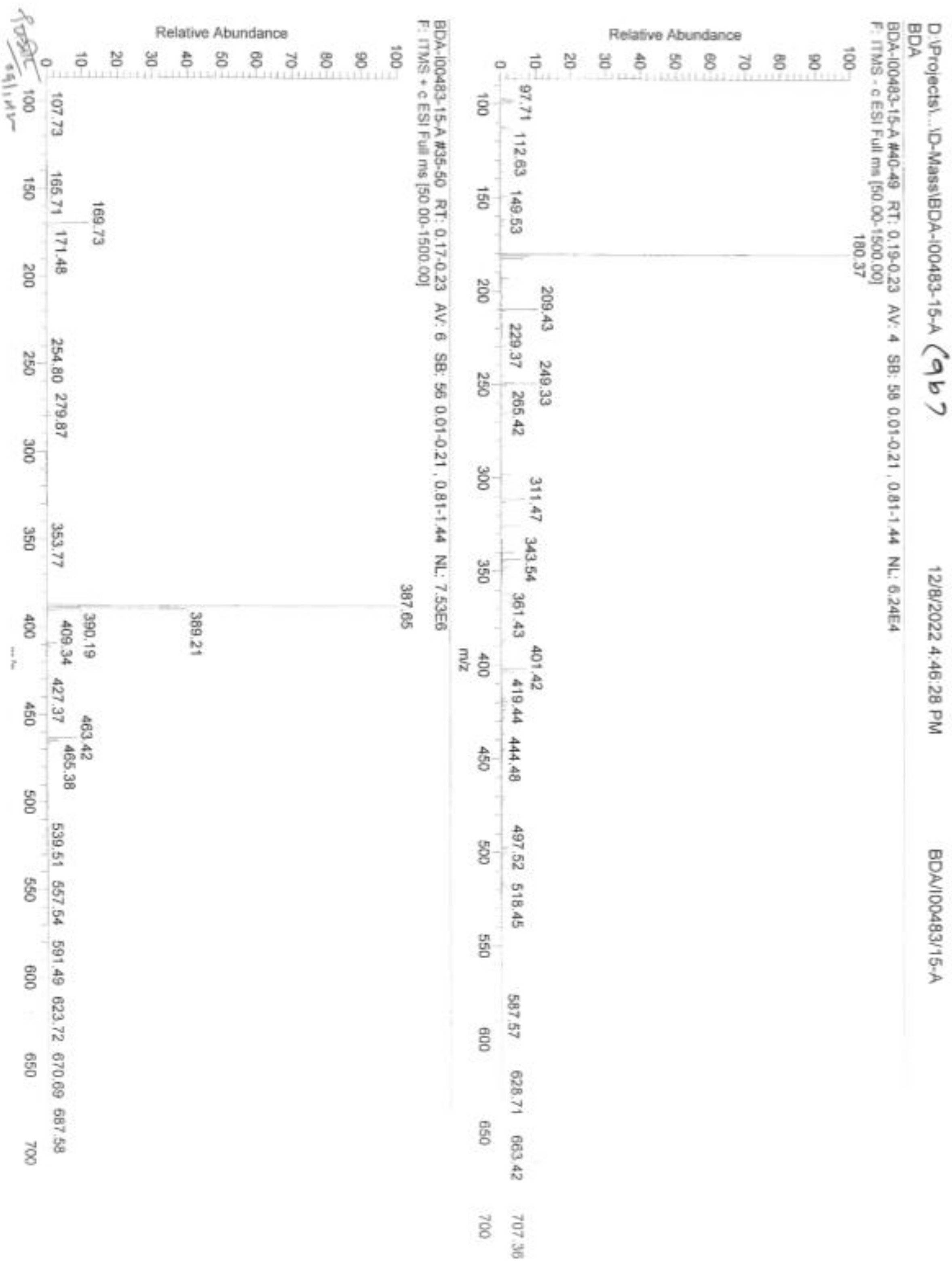

Mass spectrum of compound 9b

MICRO LABS LIMITED
API, R & D CENTRE, BOMMASANDRA (ML-27)
ANALYTICAL RESEARCH DIVISION

Sample Name	: BDA-4	Channel Description	2998 Ch1 225nm@4.8nm
Sample ID	: BDA/100483/15-A (9b)	Aqu Method Set	: BDA 4 RS
Instrument ID	: ARD_HLC_171	Processing Method	: BDA_4_RS_IMP
Test Parameter	: RS	Vial No.	: 7
Column ID	: ARD/LCC/22/019	Injection Volume	: 5 uL
Sample Set Name	: 17122L013	Analyst	: pavithra
Run Time	: 75.0 Minutes		
Column Details	: Bakerbond Q2100 C18 (150mm*4.6mm,3.0µm)		
Project Name	: DEC_2022\ARD_HLC_171		

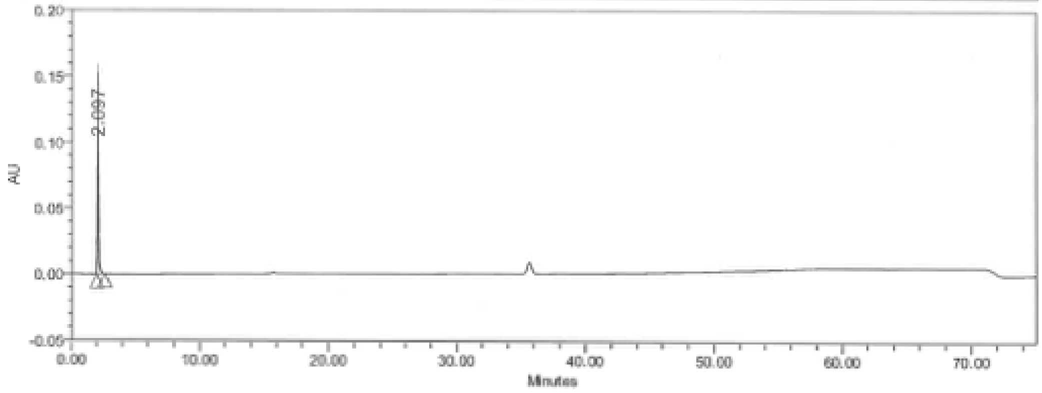

Sample Name BDA-4; Injection 1; Date Acquired 12-12-2022 20:34:20 IST; Date Processed 17-12-2022 14:27:25 IST; Result #

	Peak Name	RT	Height (µV)	Area	Int Type	RT Ratio	% Area	RRF	Corrected %Area
1	Peak1	2.10	152711	713515	BB	1.00	100.000	1.00	100.00
Sum				713515					

Result Sign Off

	SampleName	Result Id	Sign Off Full Name	Sign Off Date	Sign Off Reason
1	BDA-4	2728	Vidhi verma (Vidhi)	17-12-2022 14:29:30 IST	Sign Off Level 1, Reason: Submitted for Review
2	BDA-4	2728	Nakul Upadhyay (Nakul)	19-12-2022 15:50:27 IST	Sign Off Level 2, Reason: Reviewed and Approved

Processed By vidhi/Analyst

HPLC of compound 9b

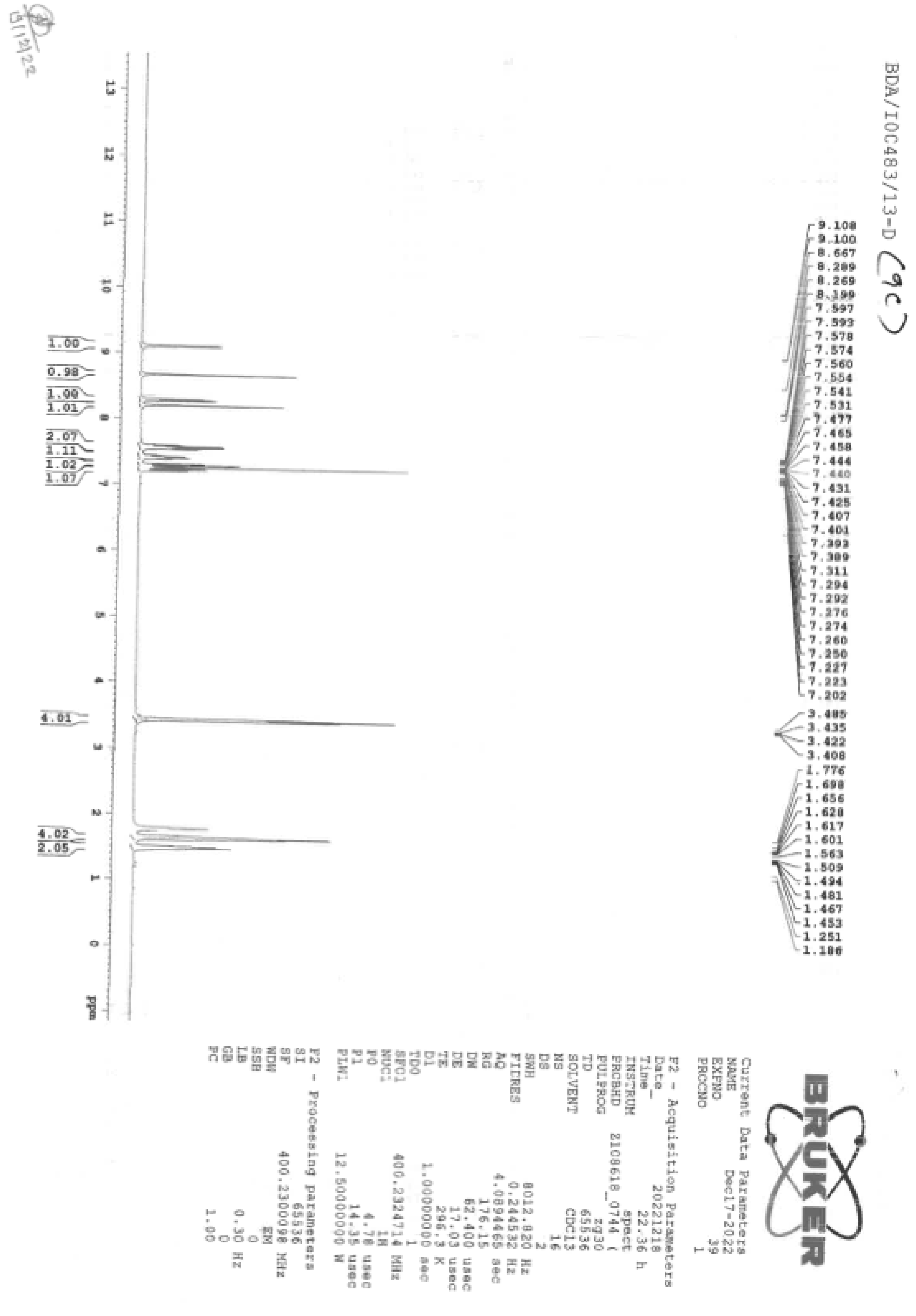

^{1}H-NMR spectrum of compound 9c

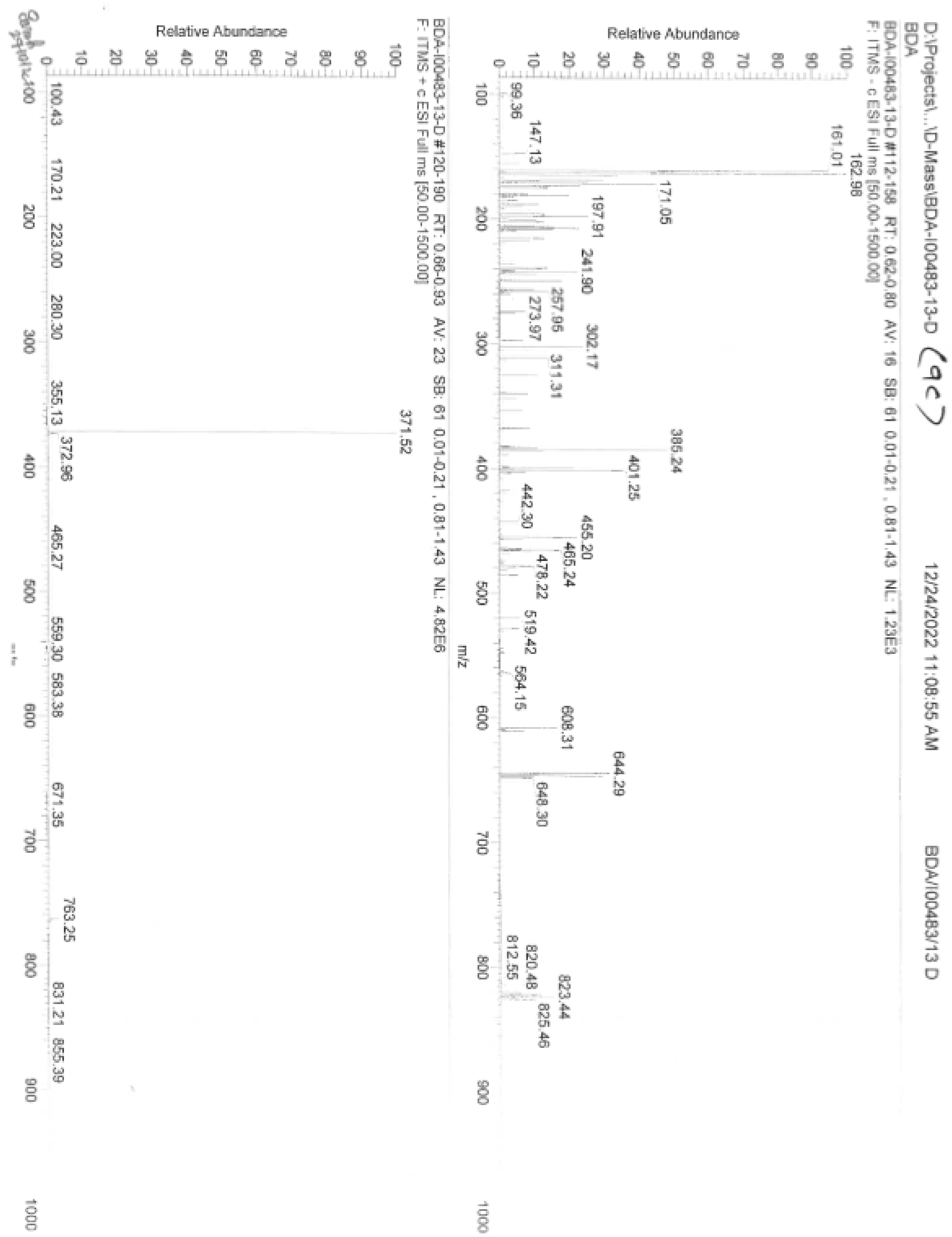

Mass spectrum of compound 9c

MICRO LABS LIMITED
API, R & D CENTRE, BOMMASANDRA (ML-27)
ANALYTICAL RESEARCH DIVISION

Sample Name	: BDA-4	Channel Description	UV Detector: Channel 1, 225 nm
Sample ID	: BDA/100483/13D (9c)	Aqu Method Set	: BDA 4 RS M1
Instrument ID	: ARD_HLC_213	Processing Method	: BDA_4_RS_M1_SPL
Test Parameter	: RS (M1)	Vial No.	: 8
Column ID	: ARD/LCC/21/032	Injection Volume	: 5 uL
Sample Set Name	: 21322L012	Analyst	: Sivanand.M
Run Time	: 75.0 Minutes		
Column Details	: Bakerbond Q2 100 C18 (150 mm * 4.6 mm , 3.0μm)		
Project Name	: DEC_2022\ARD_HLC_213		

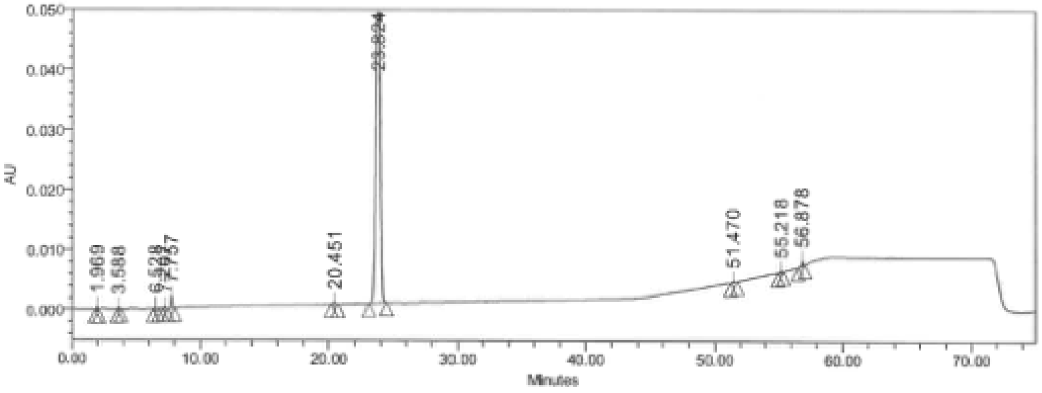

Sample Name BDA-4; Injection 1; Date Acquired 23-12-2022 20:58:25 IST; Date Processed 24-12-2022 10:54:09 IST; Result #

	Peak Name	RT	Height (μV)	Area	Int Type	RT Ratio	% Area	RRF	Corrected %Area
1	Peak1	1.97	542	3405	BB	0.08	0.233	1.00	0.23
2	Peak2	3.59	349	2363	BB	0.15	0.162	1.00	0.16
3	Peak3	6.53	395	3176	BB	0.27	0.217	1.00	0.22
4	Peak4	7.21	390	3191	BB	0.30	0.218	1.00	0.22
5	Peak5	7.76	2053	18173	BB	0.33	1.243	1.00	1.24
6	Peak6	20.45	210	3131	BB	0.86	0.214	1.00	0.21
7	Peak7	23.82	71296	1416656	BB	1.00	96.923	1.00	96.92
8	Peak8	51.47	110	1318	BB	2.16	0.090	1.00	0.09
9	Peak9	55.22	148	1448	BB	2.32	0.099	1.00	0.10

Processed By sivanandm/Analyst

HPLC of compound 9c

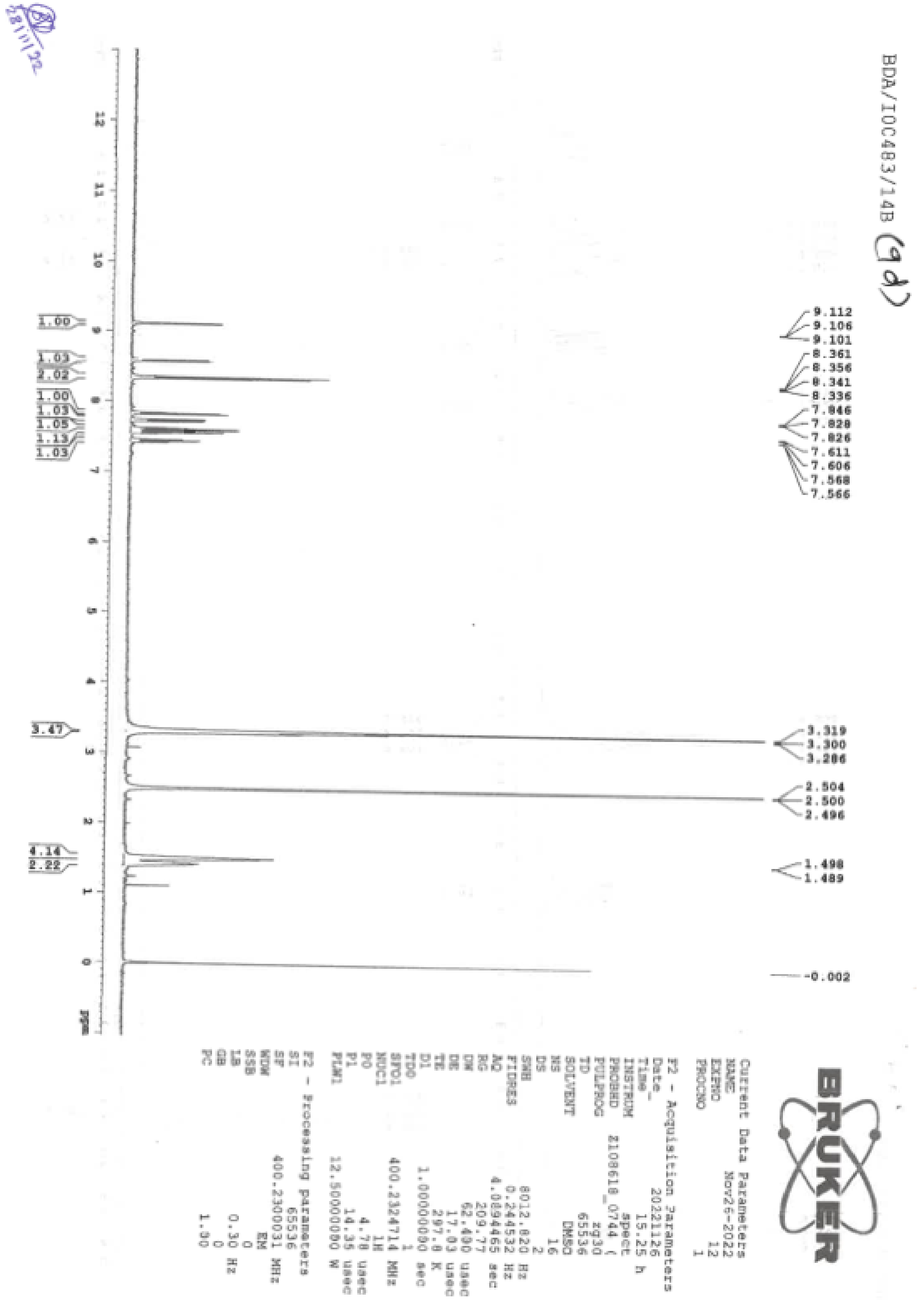

^{1}H-NMR spectrum of compound 9d

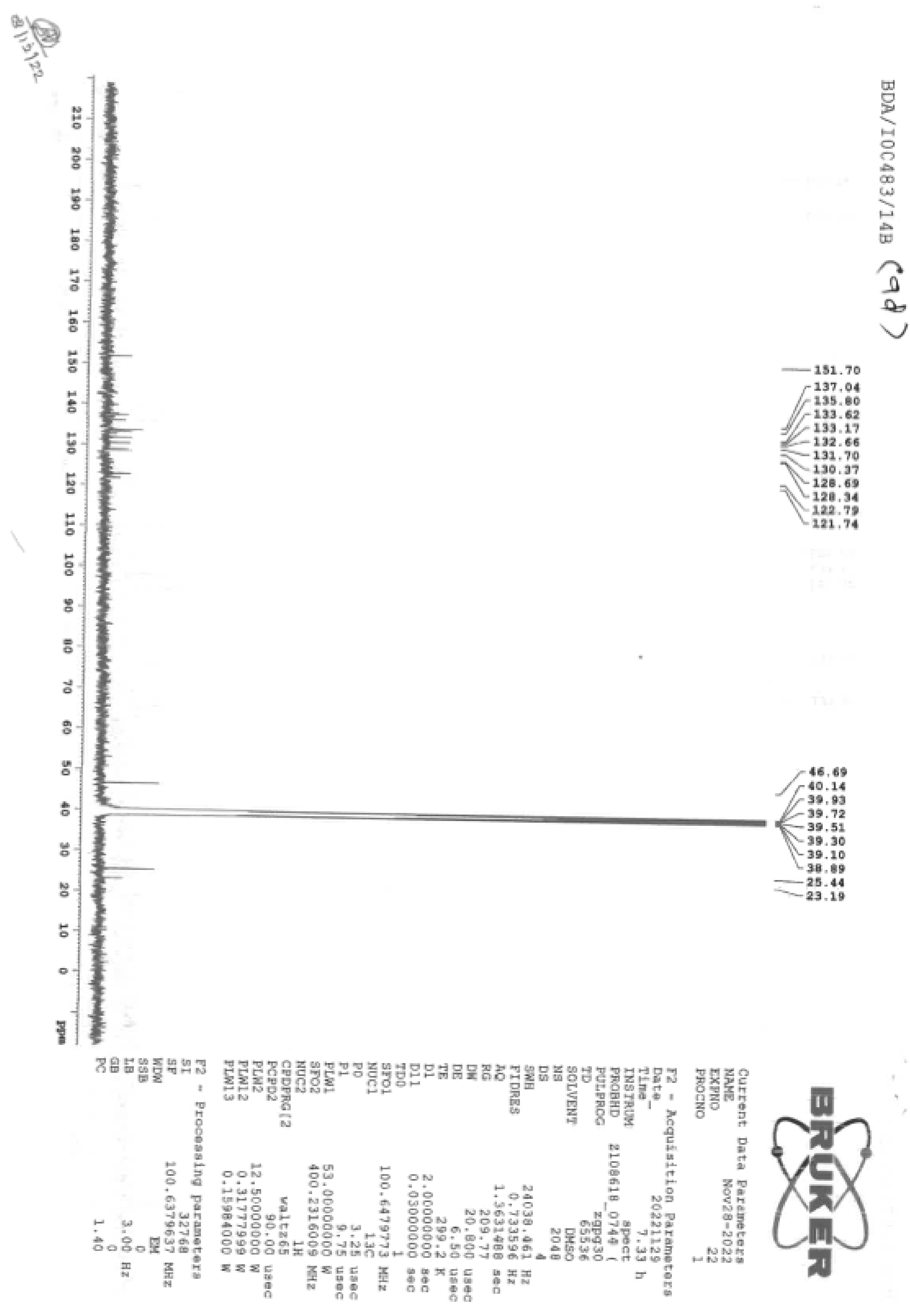

^{13}C NMR spectrum of compound 9d

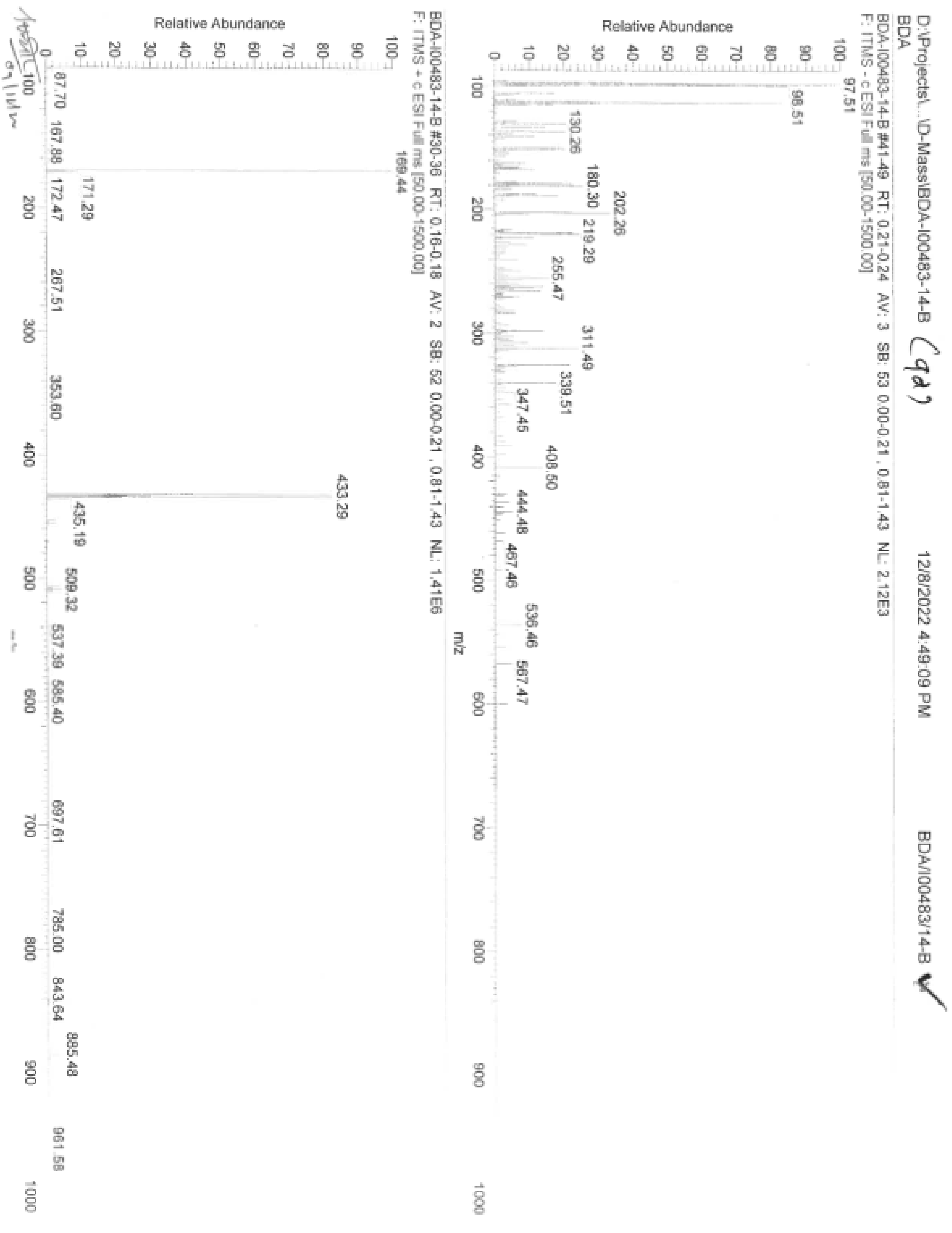

Mass spectrum of compound 9d

MICRO LABS LIMITED
API, R & D CENTRE, BOMMASANDRA (ML-27)
ANALYTICAL RESEARCH DIVISION

Sample Name	: BDA-4	Channel Description	2998 Ch1 225nm@4.8nm
Sample ID	: BDA/I00483/14-B (9d)	Aqu Method Set	: BDA 4 RS
Instrument ID	: ARD_HLC_171	Processing Method	: BDA_4_RS_IMP
Test Parameter	: RS	Vial No.	: 6
Column ID	: ARD/LCC/22/019	Injection Volume	: 5 uL
Sample Set Name	: 17122L013	Analyst	: pavithra
Run Time	: 75.0 Minutes		
Column Details	: Bakerbond Q2100 C18 (150mm*4.6mm,3.0µm)		
Project Name	: DEC_2022\ARD_HLC_171		

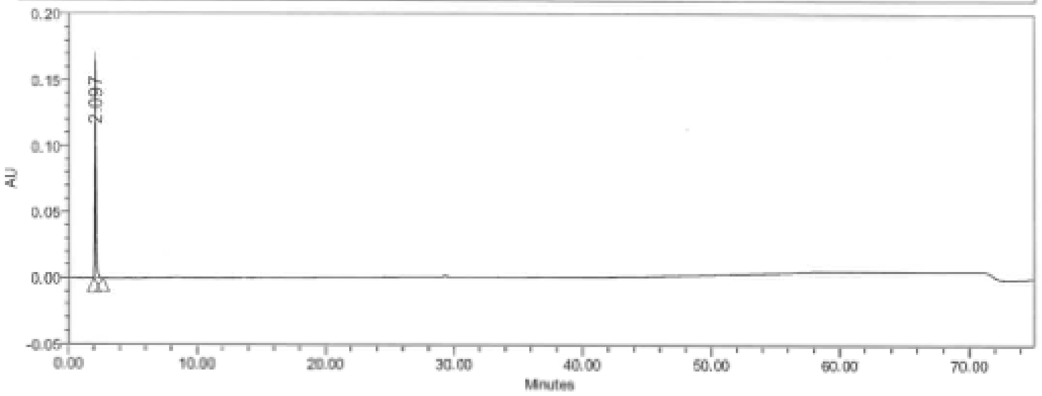

Sample Name BDA-4; Injection 1; Date Acquired 12-12-2022 19:18:32 IST; Date Processed 17-12-2022 14:27:06 IST; Result #

	Peak Name	RT	Height (µV)	Area	Int Type	RT Ratio	% Area	RRF	Corrected %Area
1	Peak1	2.10	164413	757917	BB	1.00	100.000	1.00	100.00
Sum				757917					

Result Sign Off

	SampleName	Result Id	Sign Off Full Name	Sign Off Date	Sign Off Reason
1	BDA-4	2727	Vidhi verma (Vidhi)	17-12-2022 14:29:30 IST	Sign Off Level 1, Reason: Submitted for Review
2	BDA-4	2727	Nakul Upadhyay (Nakul)	19-12-2022 15:50:27 IST	Sign Off Level 2, Reason: Reviewed and Approved

Processed By vidhi/Analyst

HPLC of compound 9d

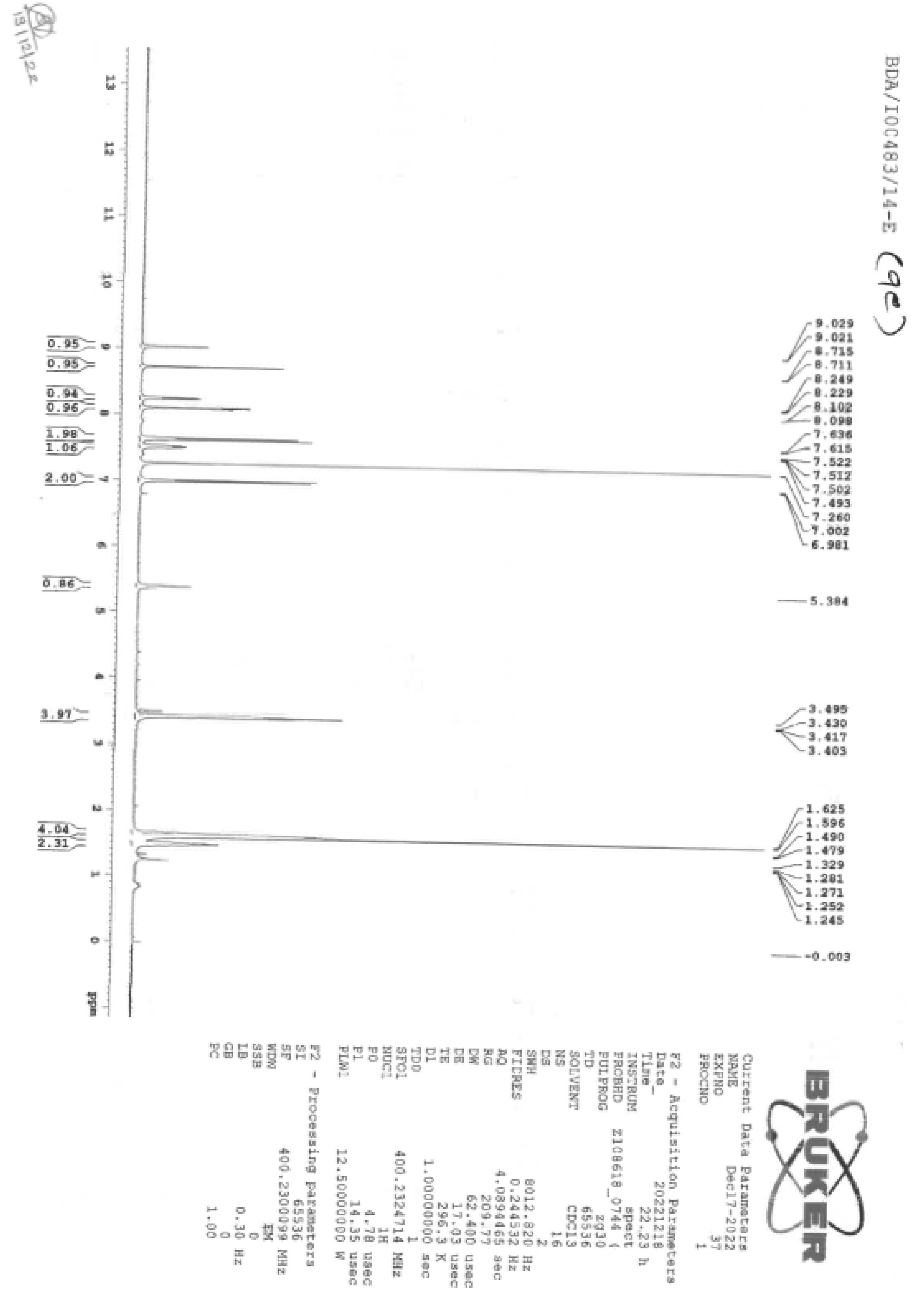

^{1}H-NMR spectrum of compound 9e

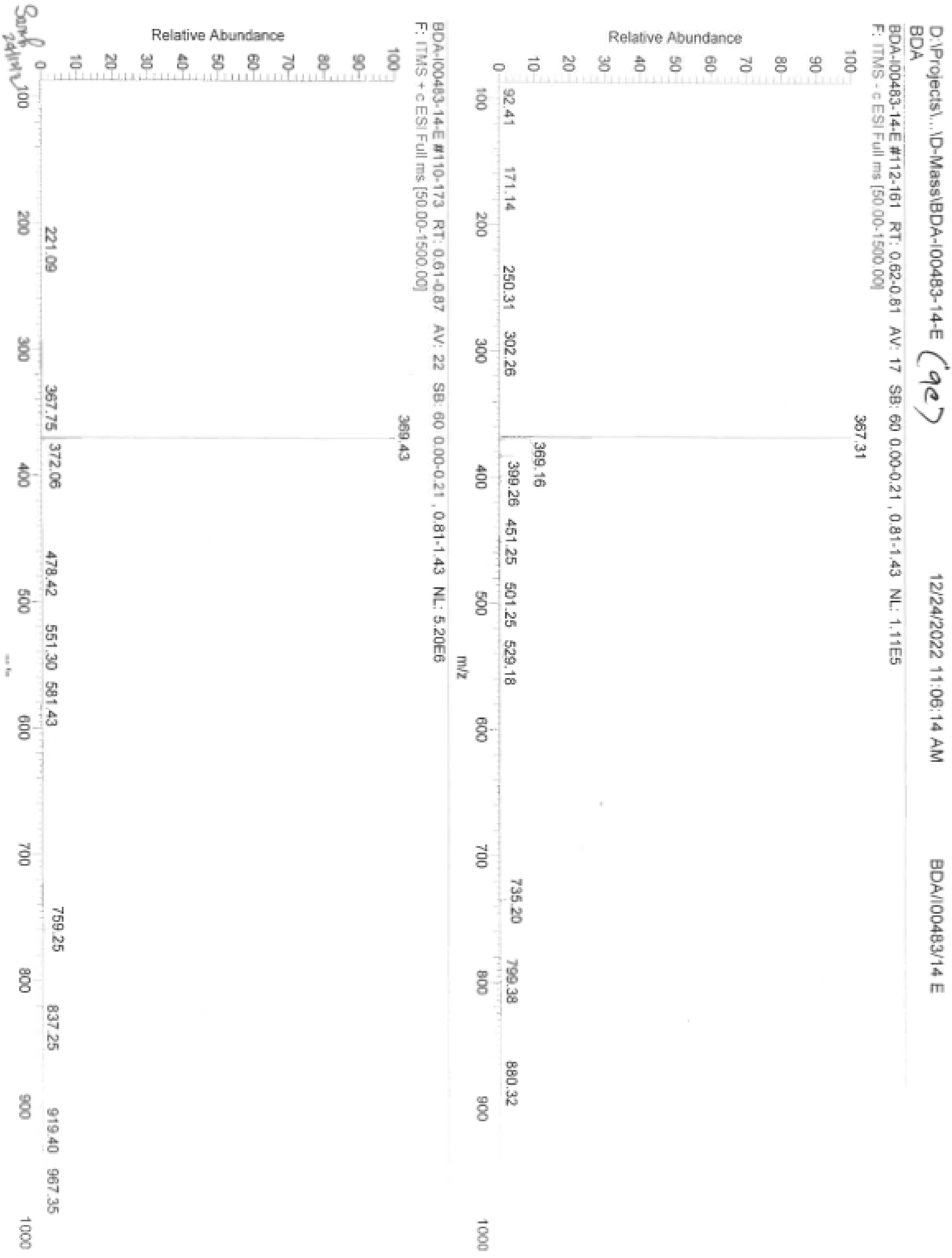

Mass spectrum of compound 9e

MICRO LABS LIMITED
API, R & D CENTRE, BOMMASANDRA (ML-27)
ANALYTICAL RESEARCH DIVISION

Sample Name : BDA-4
Sample ID : BDA/I00483/14E (9e)
Instrument ID : ARD_HLC_213
Test Parameter : RS (M1)
Column ID : ARD/LCC/21/032
Sample Set Name : 21322L012
Run Time : 75.0 Minutes
Column Details : Bakerbond Q2 100 C18 (150 mm * 4.6 mm , 3.0µm)
Project Name : DEC_2022\ARD_HLC_213

Channel Description UV Detector: Channel 1, 225 nm
Aqu Method Set : BDA 4 RS M1
Processing Method : BDA_4_RS_M1_SPL
Vial No. : 7
Injection Volume : 5 uL
Analyst : Sivanand.M

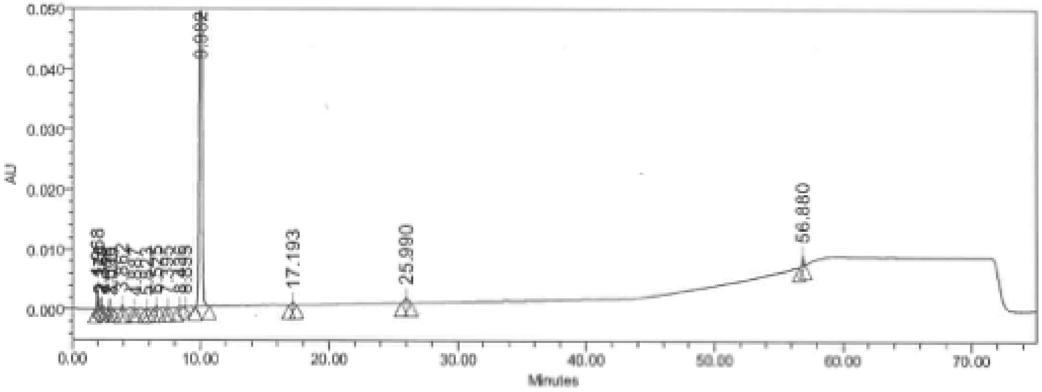

Sample Name BDA-4; Injection 1; Date Acquired 23-12-2022 19:42:54 IST; Date Processed 24-12-2022 10:52:18 IST; Result #

	Peak Name	RT	Height (µV)	Area	Int Type	RT Ratio	% Area	RRF	Corrected %Area
1	Peak1	1.97	3238	16467	BV	0.20	0.802	1.00	0.80
2	Peak2	2.18	400	2797	VV	0.22	0.136	1.00	0.14
3	Peak3	2.37	522	5071	VV	0.24	0.247	1.00	0.25
4	Peak4	2.81	201	1963	VV	0.28	0.096	1.00	0.10
5	Peak5	3.04	155	988	VB	0.30	0.048	1.00	0.05
6	Peak6	3.86	759	5058	BB	0.39	0.246	1.00	0.25
7	Peak7	4.89	103	652	BB	0.49	0.032	1.00	0.03
8	Peak8	5.82	109	854	BB	0.58	0.042	1.00	0.04
9	Peak9	6.53	418	3538	BB	0.65	0.172	1.00	0.17

Processed By sivanandm/Analyst

HPLC of compound 9e

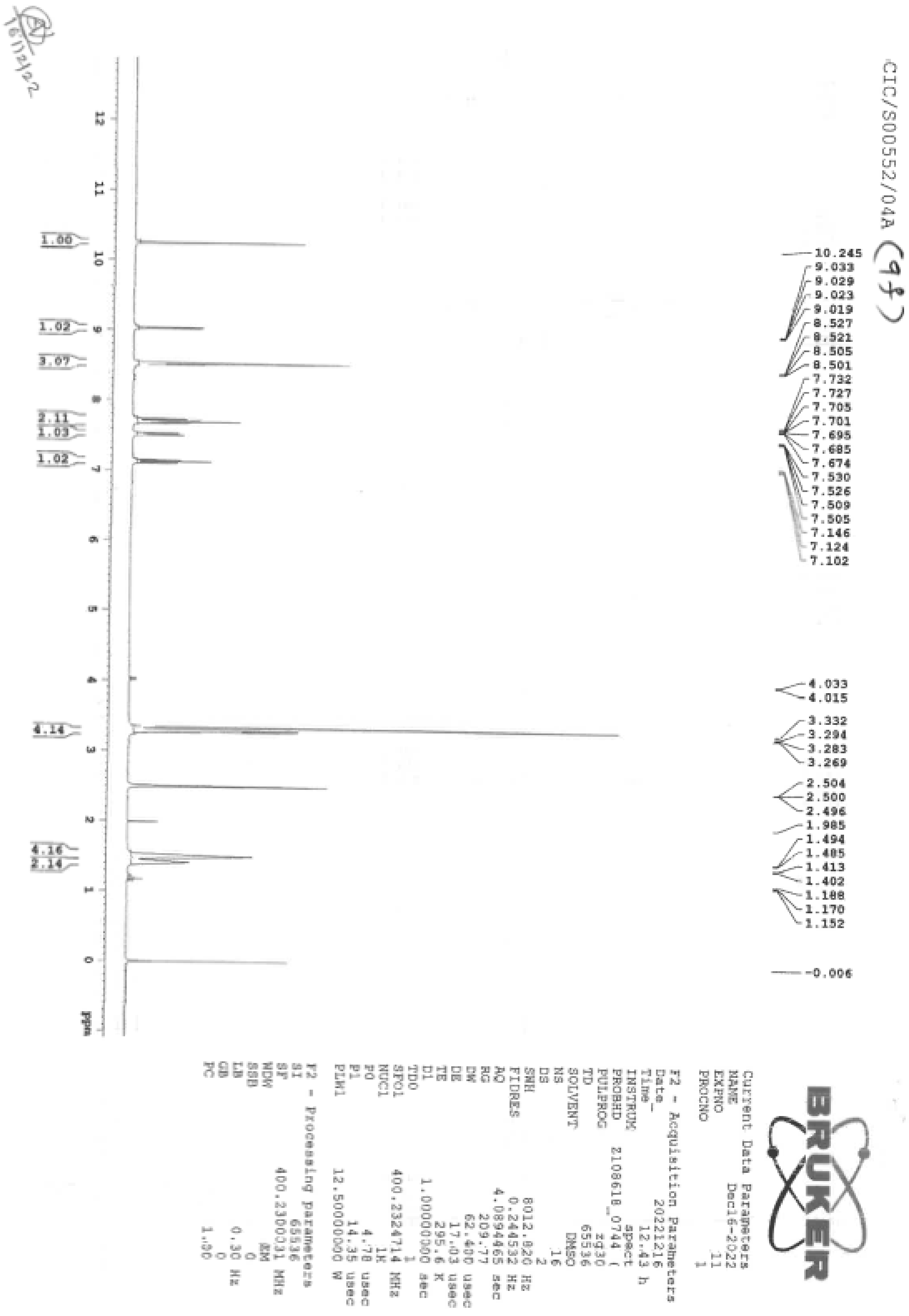

^{1}H-NMR spectrum of compound 9f

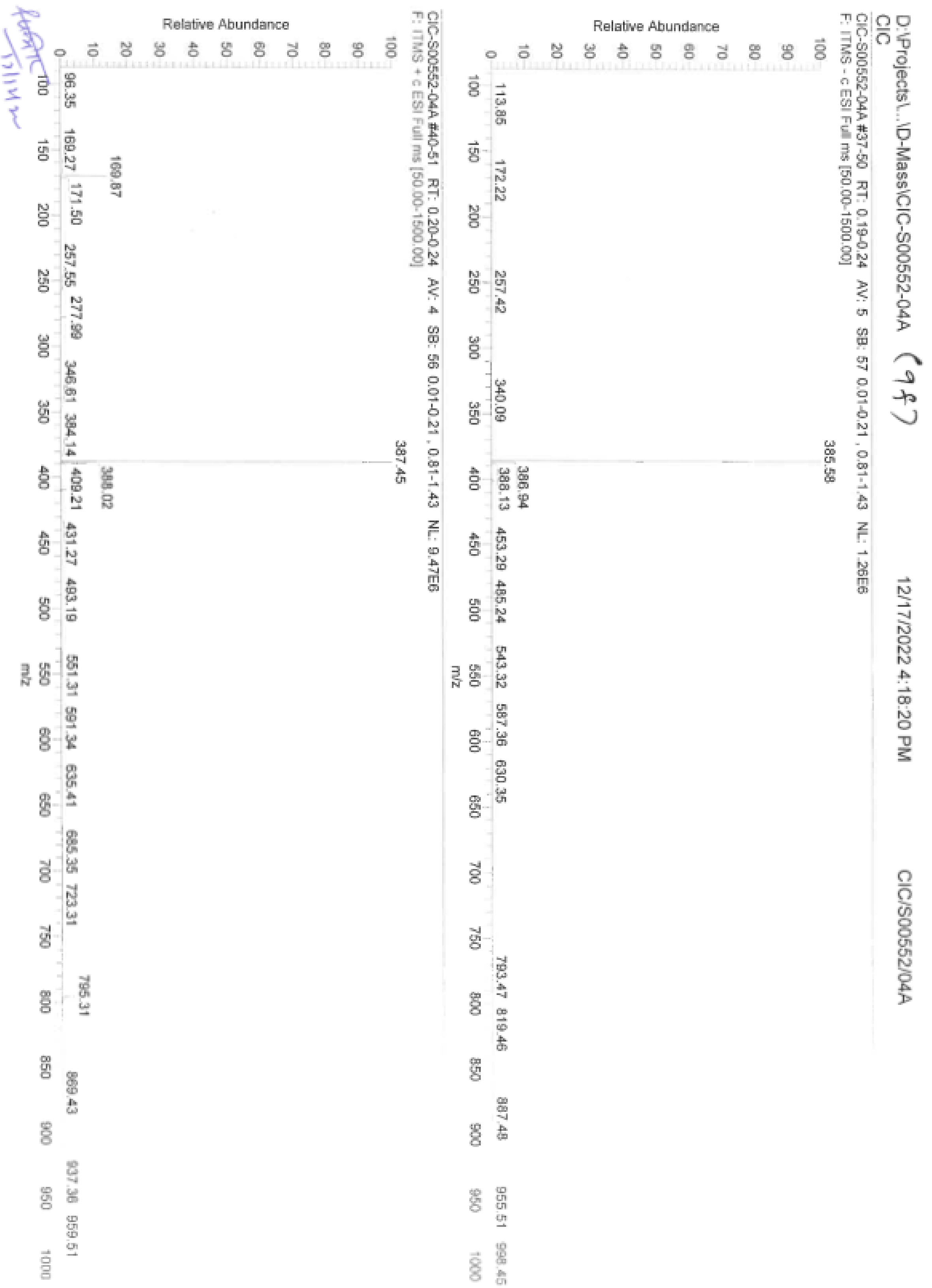

Mass spectrum of compound 9f

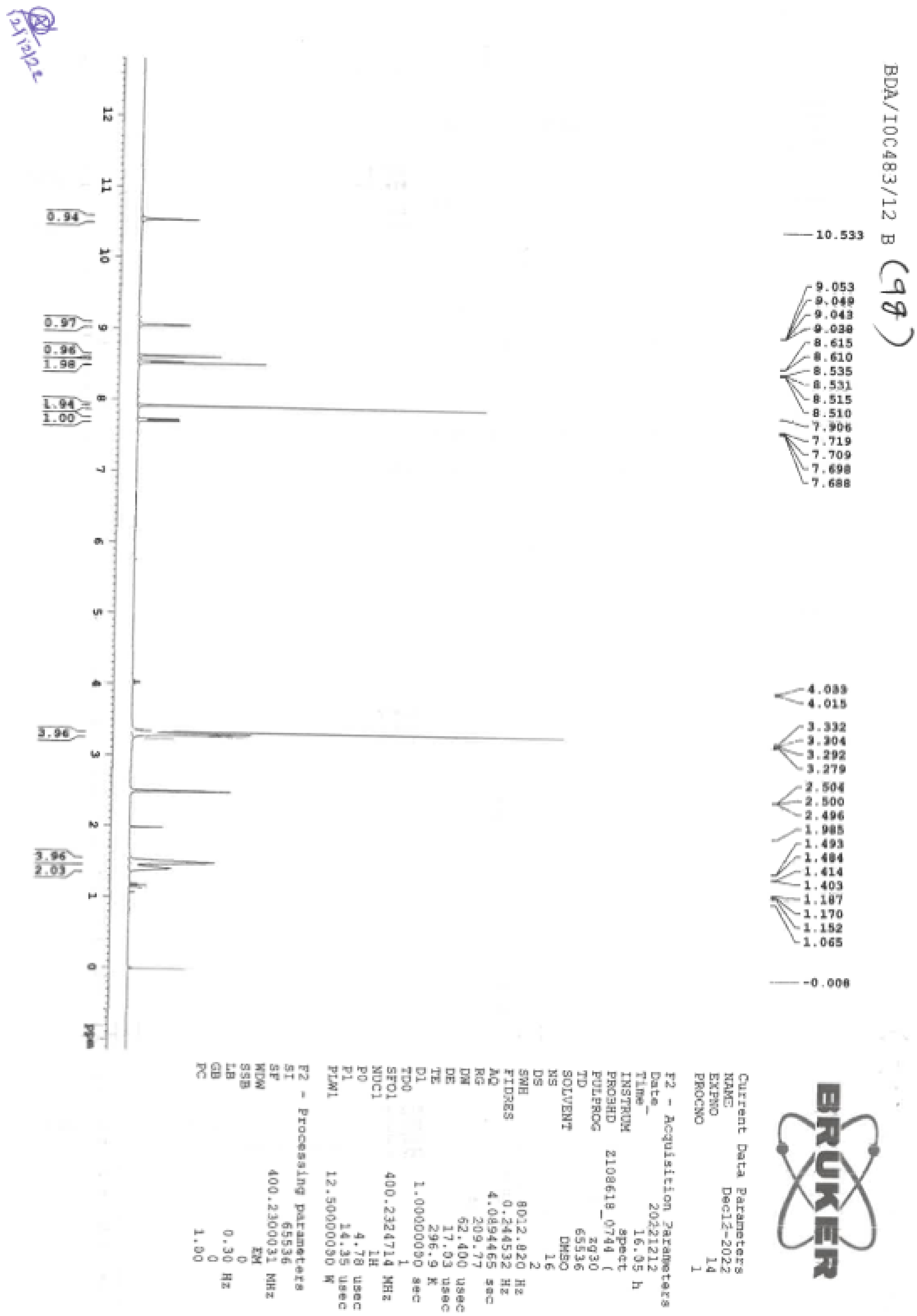

^{1}H-NMR spectrum of compound 9g

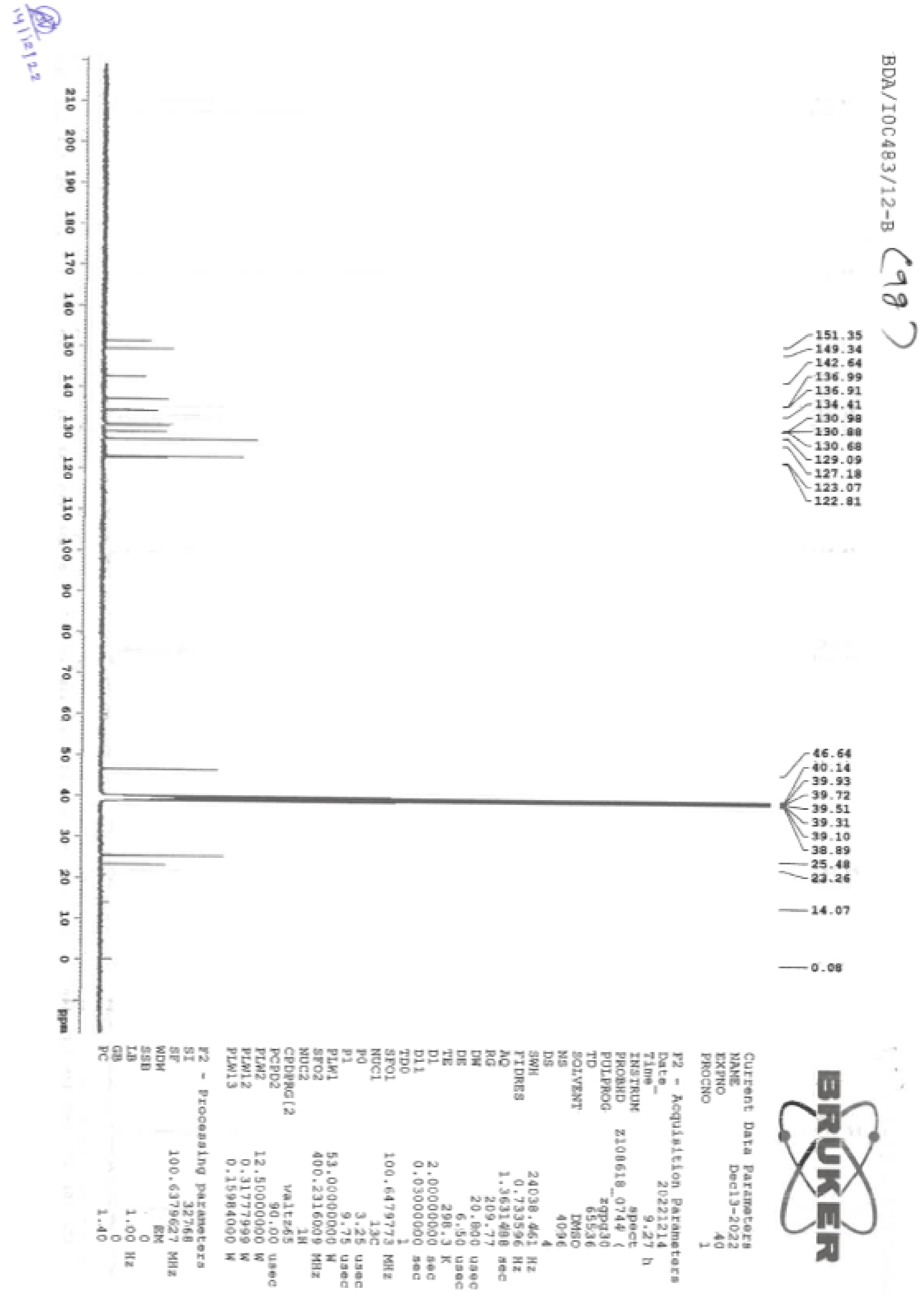

^{13}C NMR spectrum of compound 9g

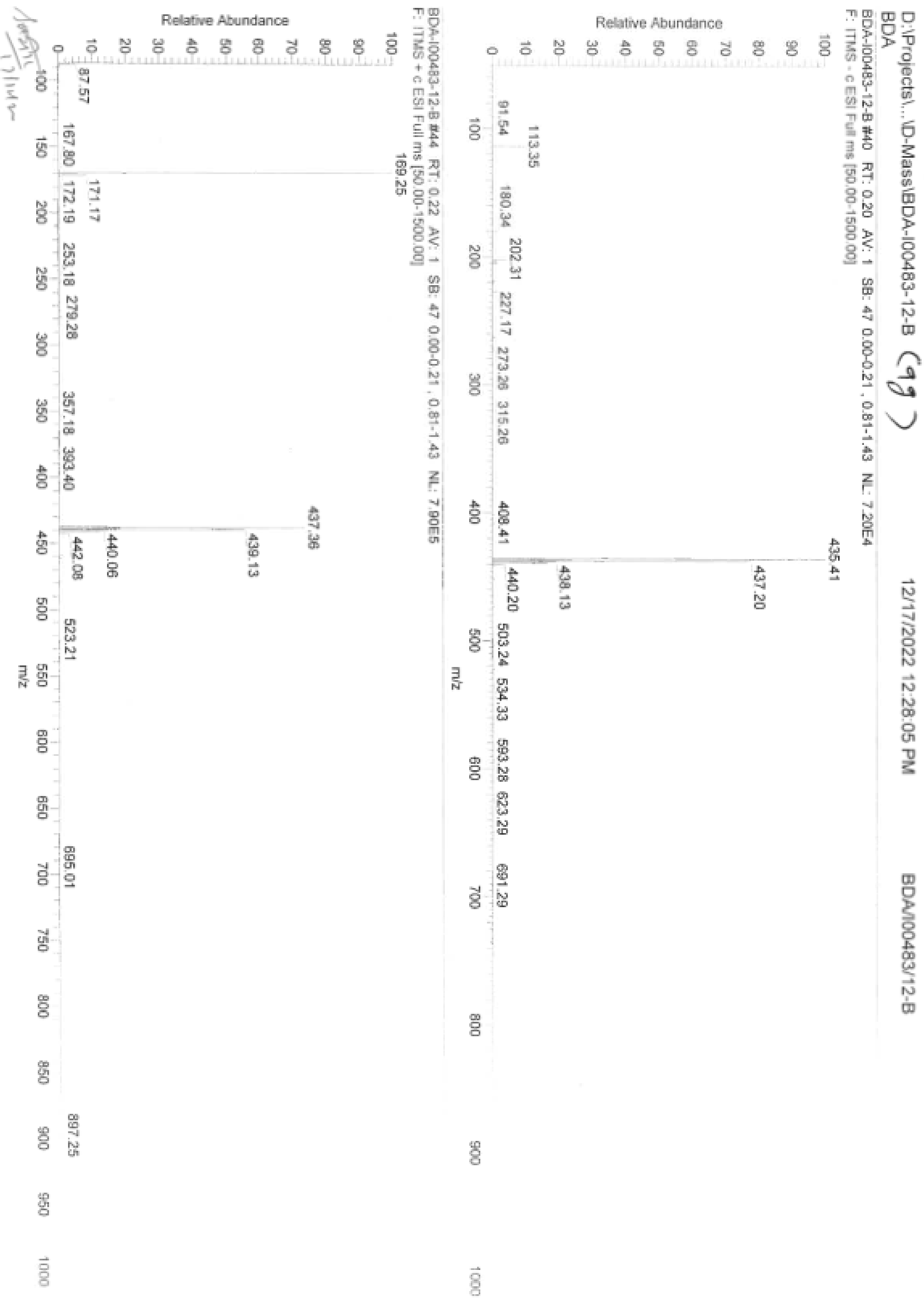

Mass spectrum of compound 9g

MICRO LABS LIMITED
API, R & D CENTRE, BOMMASANDRA (ML-27)
ANALYTICAL RESEARCH DIVISION

Sample Name	: BDA-4	Channel Description	UV Detector: Channel 1, 225 nm
Sample ID	: BDA/100483/12B (9g)	Aqu Method Set	: BDA 4 RS M1
Instrument ID	: ARD_HLC_213	Processing Method	: BDA_4_RS_M1_SPL
Test Parameter	: RS (M1)	Vial No.	: 9
Column ID	: ARD/LCC/21/032	Injection Volume	: 5 uL
Sample Set Name	: 21322L012	Analyst	: Sivanand.M
Run Time	: 75.0 Minutes		
Column Details	: Bakerbond Q2 100 C18 (150 mm * 4.6 mm , 3.0µm)		
Project Name	: DEC_2022\ARD_HLC_213		

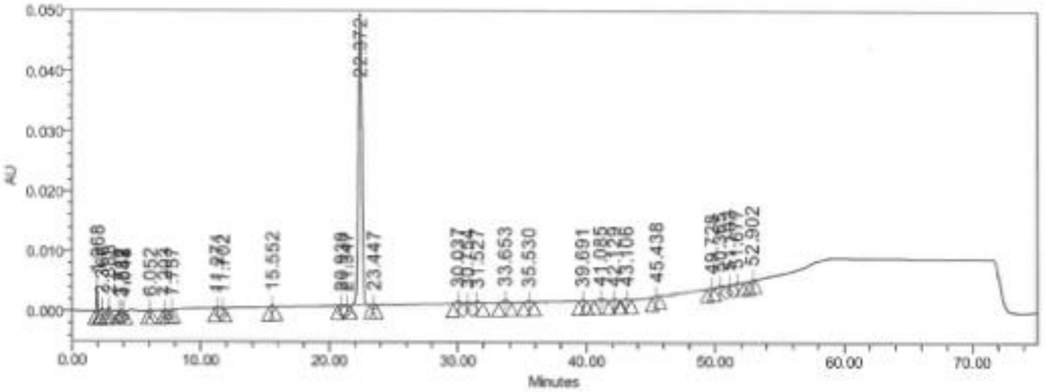

Sample Name BDA-4; Injection 1; Date Acquired 23-12-2022 22:13:59 IST; Date Processed 24-12-2022 11:00:34 IST; Result #

	Peak Name	RT	Height (µV)	Area	Int Type	RT Ratio	% Area	RRF	Corrected %Area
1	Peak1	1.97	3964	19959	BB	0.09	1.954	1.00	1.95
2	Peak2	2.37	346	2413	BB	0.11	0.236	1.00	0.24
3	Peak3	2.89	611	3559	BB	0.13	0.348	1.00	0.35
4	Peak4	3.71	111	654	BV	0.17	0.064	1.00	0.06
5	Peak5	3.88	71	423	VV	0.17	0.041	1.00	0.04
6	Peak6	4.05	101	596	VB	0.18	0.058	1.00	0.06
7	Peak7	6.05	107	831	BB	0.27	0.081	1.00	0.08
8	Peak8	7.20	59	453	BB	0.32	0.044	1.00	0.04
9	Peak9	7.76	89	685	BB	0.35	0.067	1.00	0.07

Processed By sivanandm/Analyst

HPLC of compound 9g

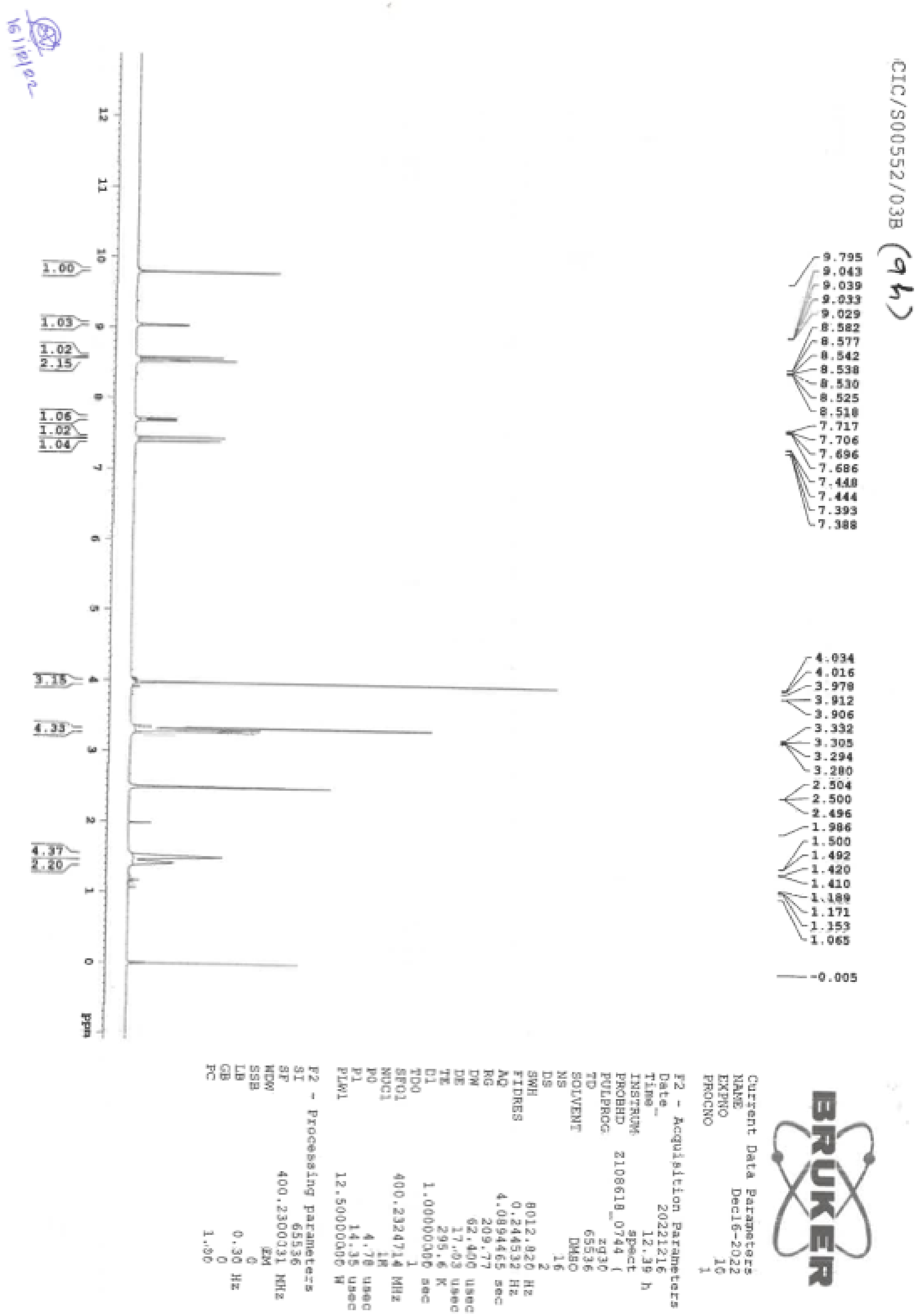

^{1}H-NMR spectrum of compound 9h

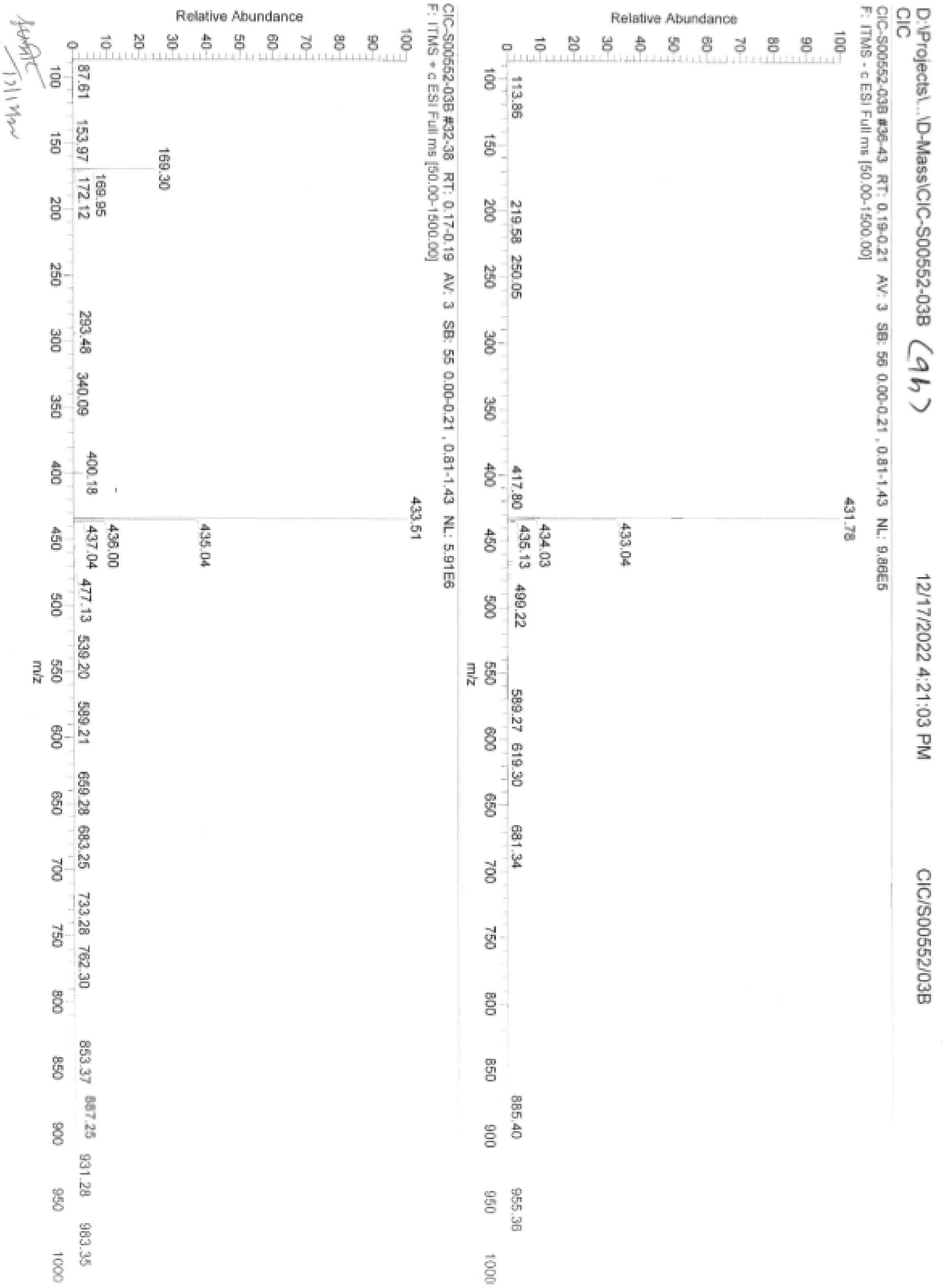

Mass spectrum of compound 9h

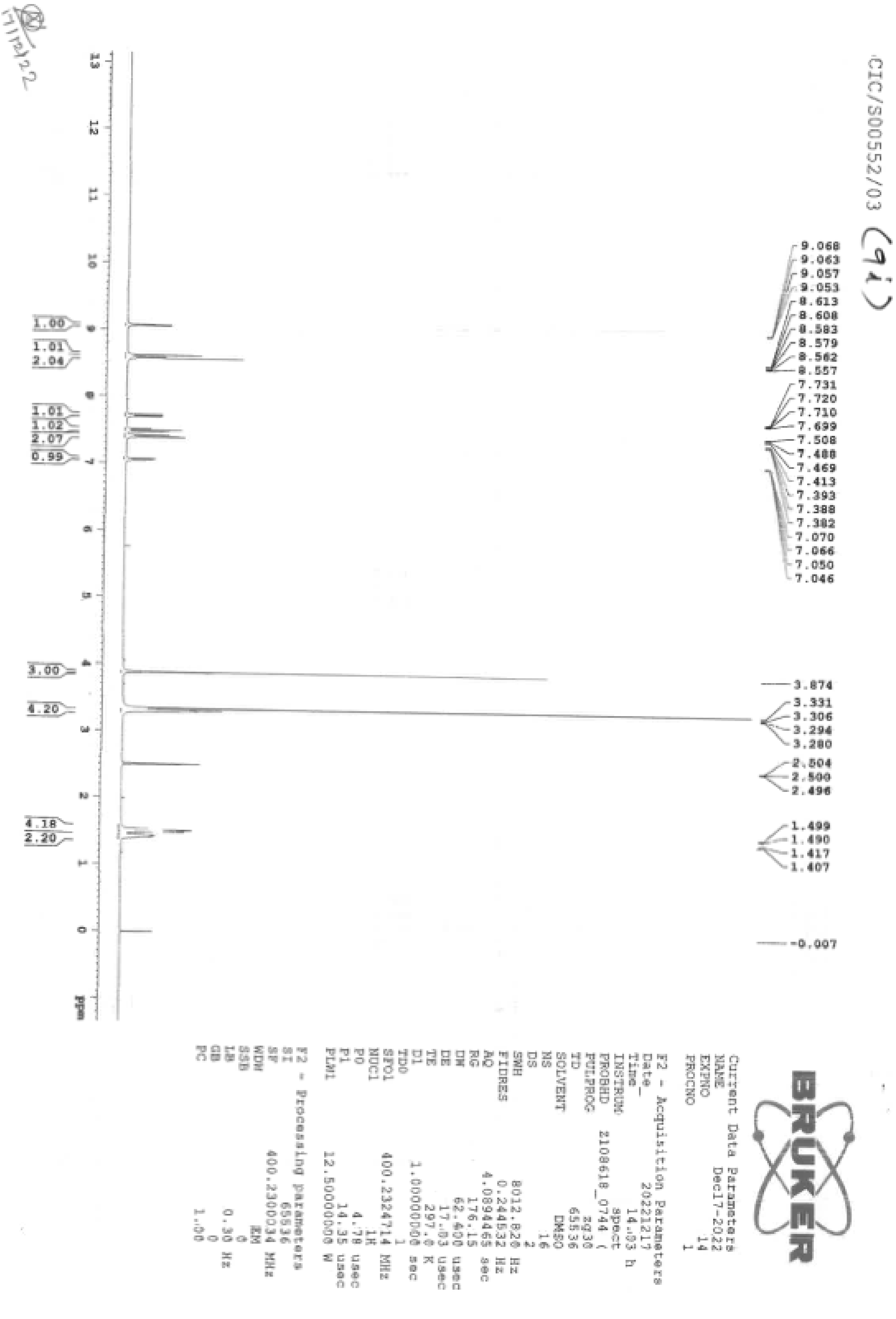

^{1}H-NMR spectrum of compound 9i

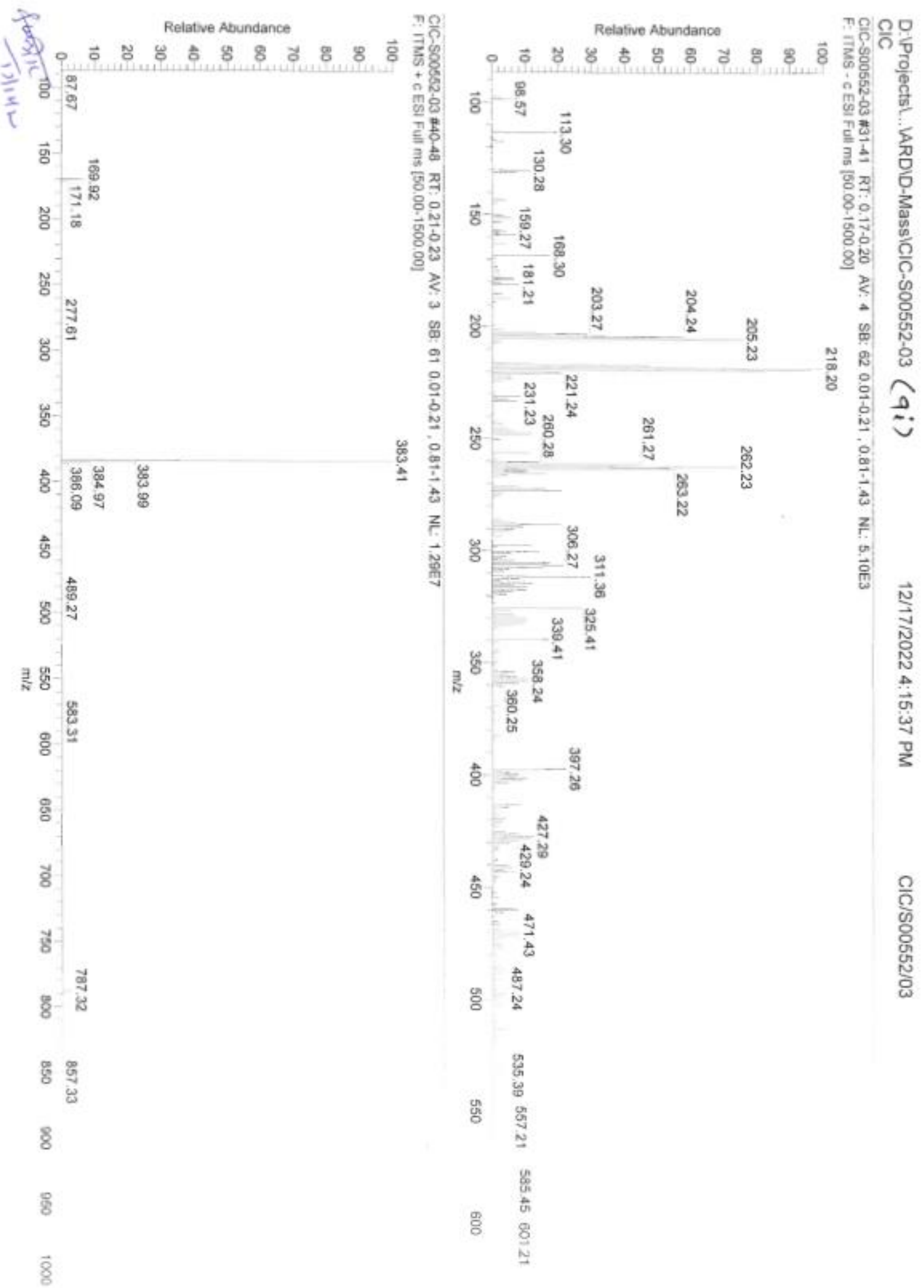

Mass spectrum of compound 9i

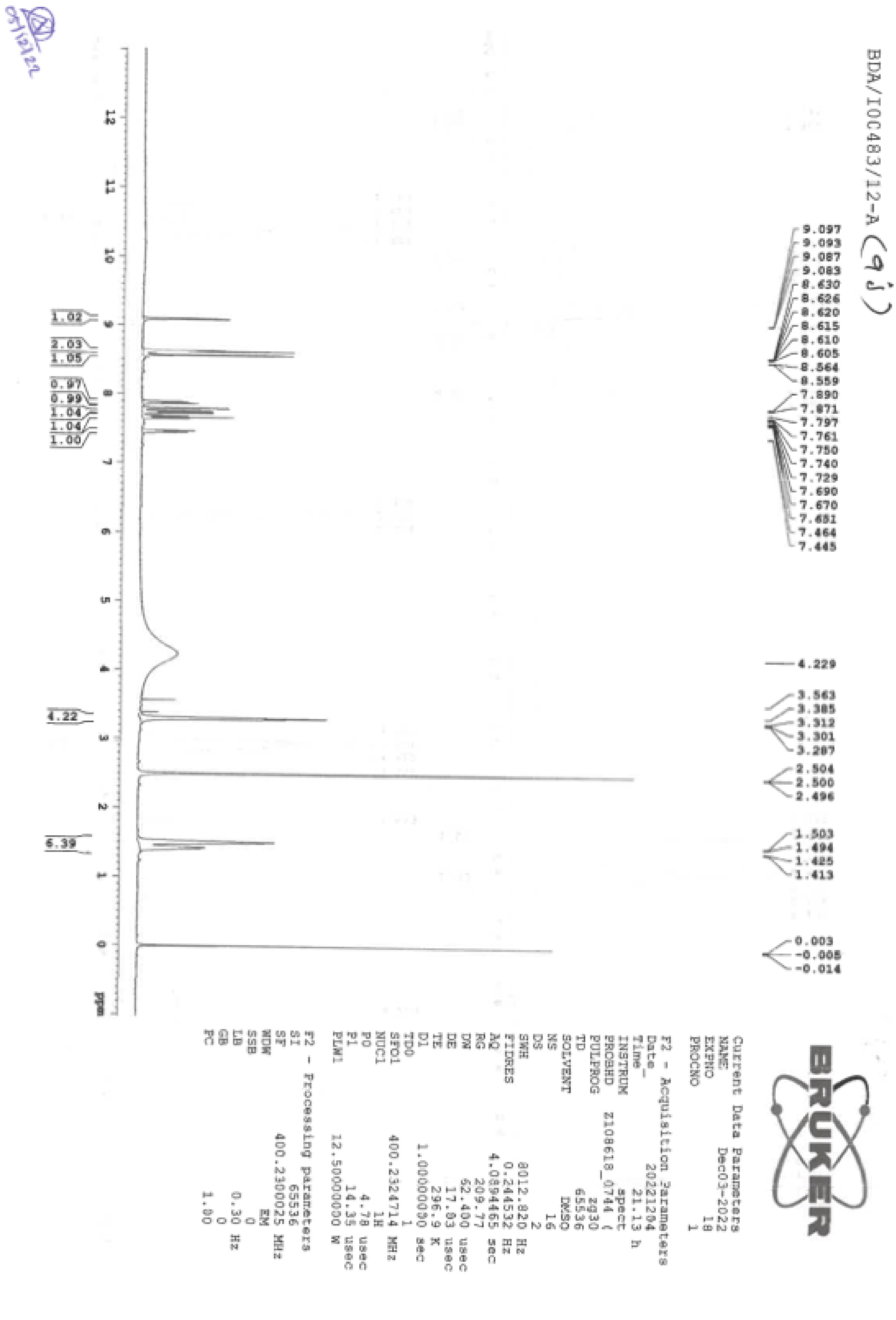

^{1}H-NMR spectrum of compound 9j

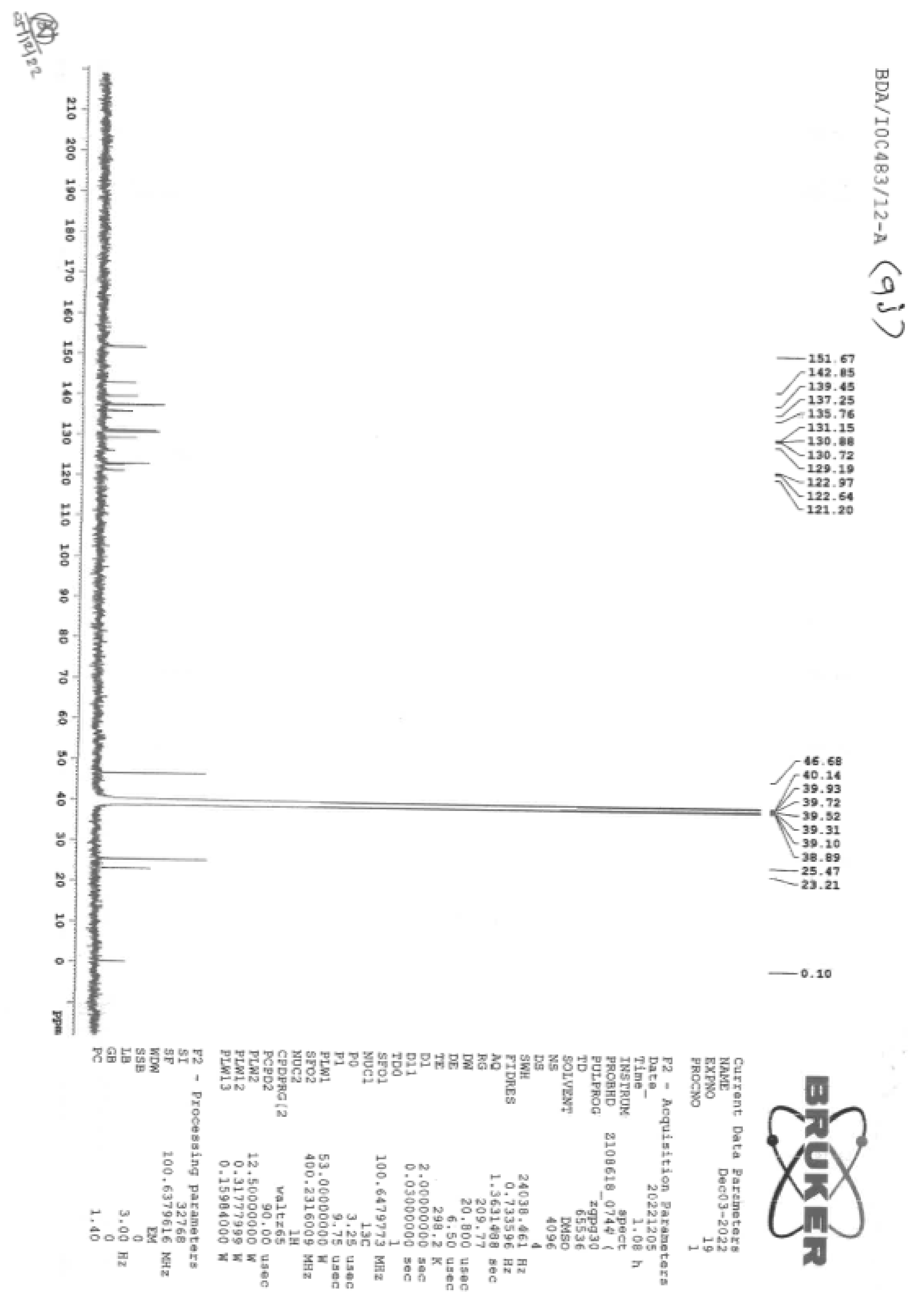

^{13}C NMR spectrum of compound 9j

MICRO LABS LIMITED
API, R & D CENTRE, BOMMASANDRA (ML-27)
ANALYTICAL RESEARCH DIVISION

Sample Name : BDA-4
Sample ID : BDA/I00483/12-A (9j)
Instrument ID : ARD_HLC_171
Test Parameter : RS
Column ID : ARD/LCC/22/019
Sample Set Name : 17122L013
Run Time : 75.0 Minutes
Column Details : Bakerbond Q2100 C18 (150mm*4.6mm,3.0μm)
Project Name : DEC_2022\ARD_HLC_171

Channel Description 2998 Ch1 225nm@4.8nm
Aqu Method Set : BDA 4 RS
Processing Method : BDA_4_RS_IMP
Vial No. : 9
Injection Volume : 5 uL
Analyst : pavithra

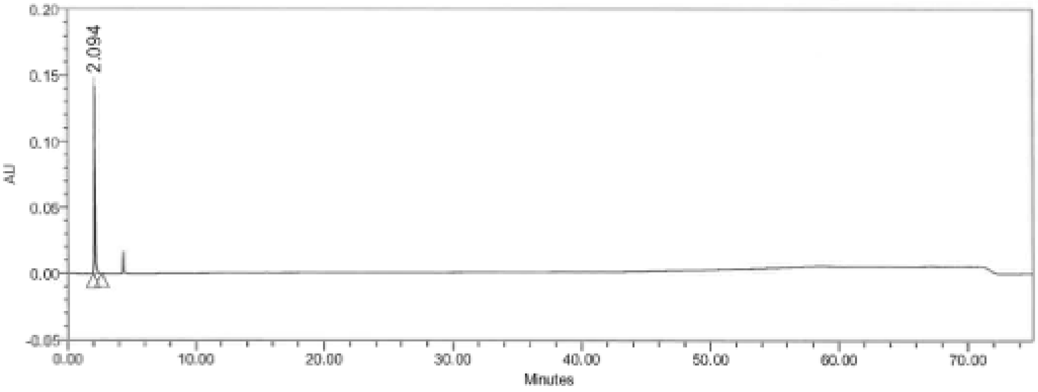

Sample Name BDA-4; Injection 1; Date Acquired 12-12-2022 23:05:57 IST; Date Processed 17-12-2022 14:28:39 IST; Result #

	Peak Name	RT	Height (μV)	Area	Int Type	RT Ratio	% Area	RRF	Corrected %Area
1	Peak1	2.09	142504	642311	BB	1.00	100.000	1.00	100.00
Sum				642311					

Result Sign Off

	SampleName	Result Id	Sign Off Full Name	Sign Off Date	Sign Off Reason
1	BDA-4	2730	Vidhi verma (Vidhi)	17-12-2022 14:29:30 IST	Sign Off Level 1, Reason: Submitted for Review
2	BDA-4	2730	Nakul Upadhyay (Nakul)	19-12-2022 15:50:27 IST	Sign Off Level 2, Reason: Reviewed and Approved

Processed By vidhi/Analyst

HPLC of compound 9j

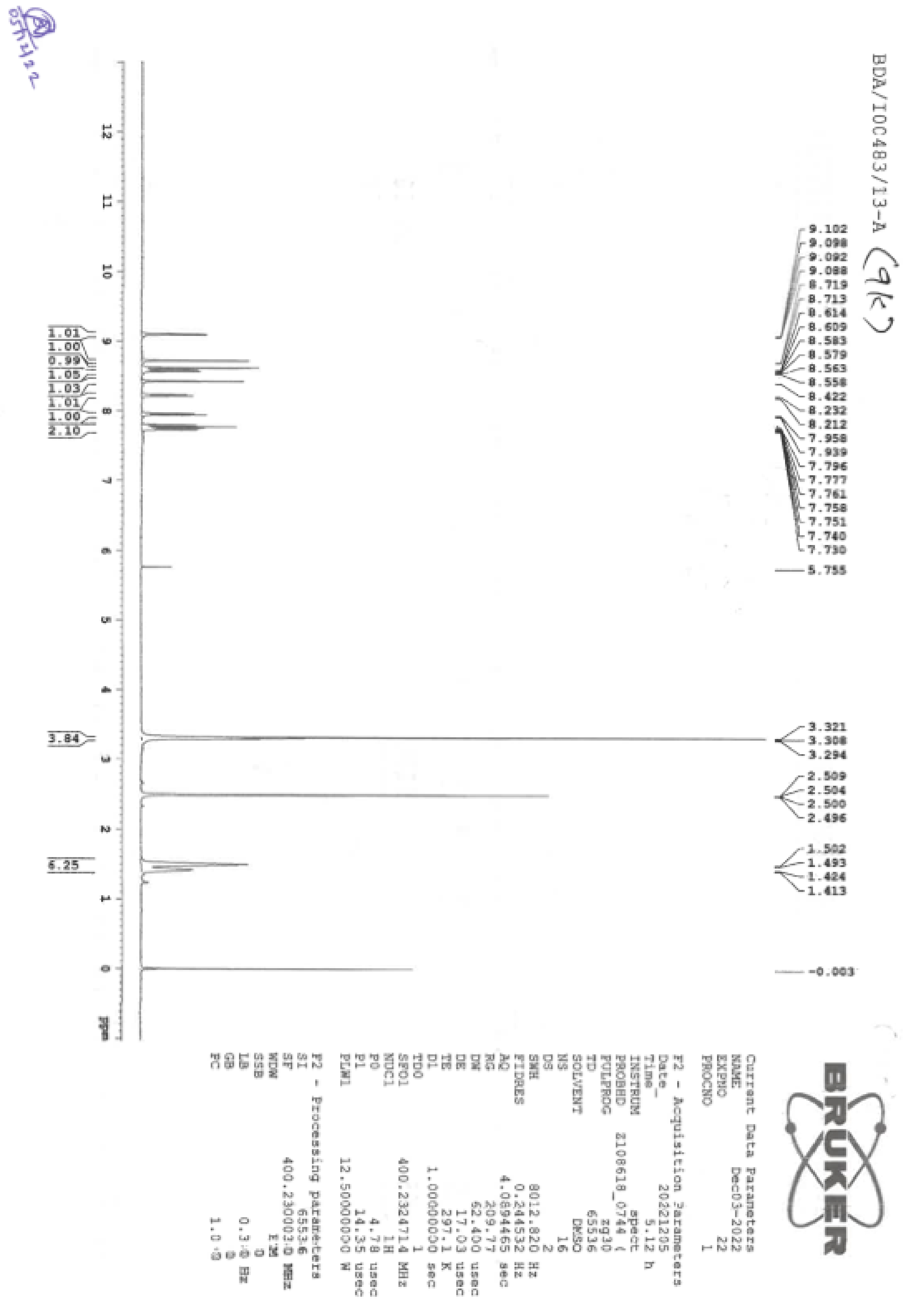

^{1}H-NMR spectrum of compound 9k

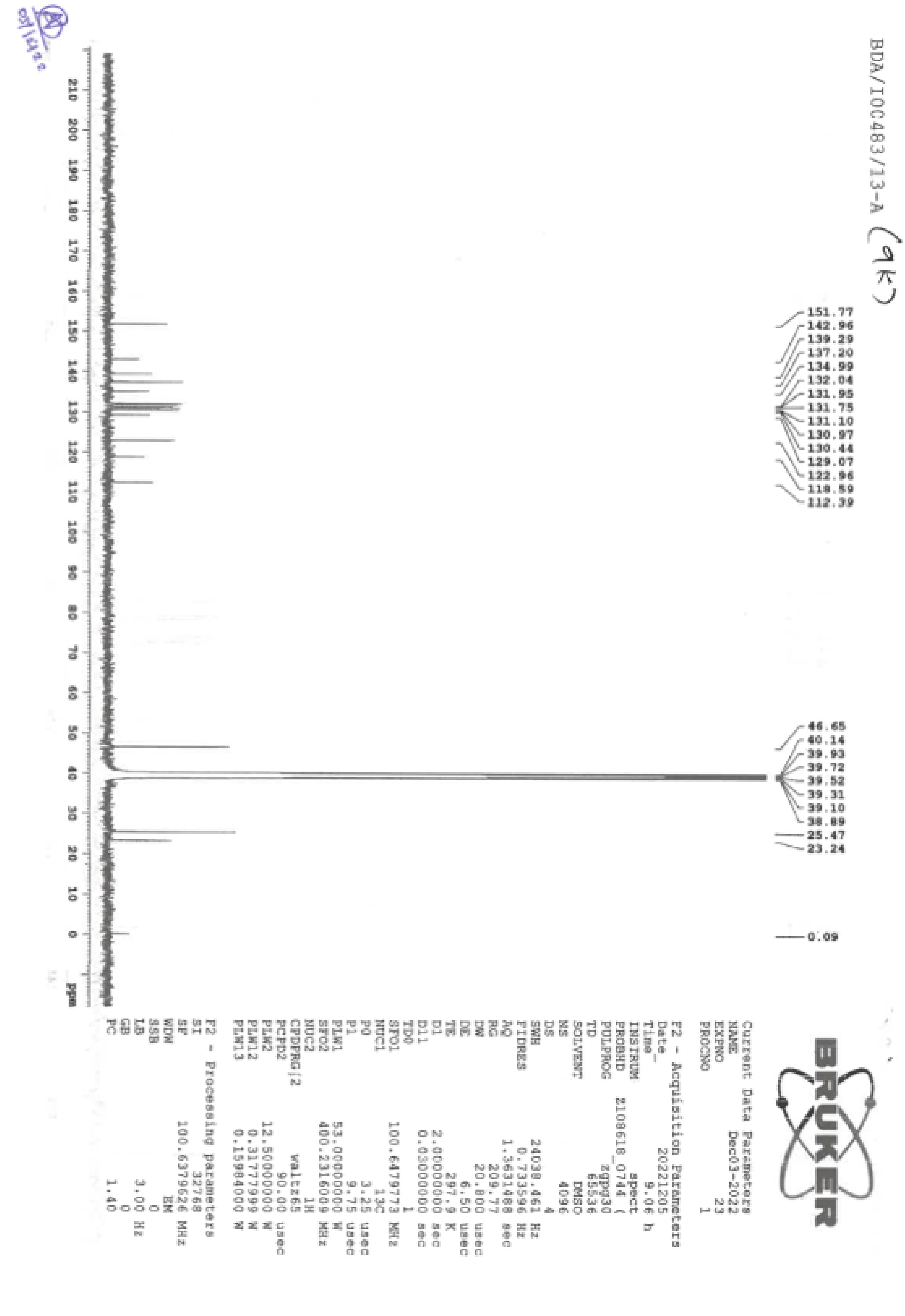

^{13}C NMR spectrum of compound 9k

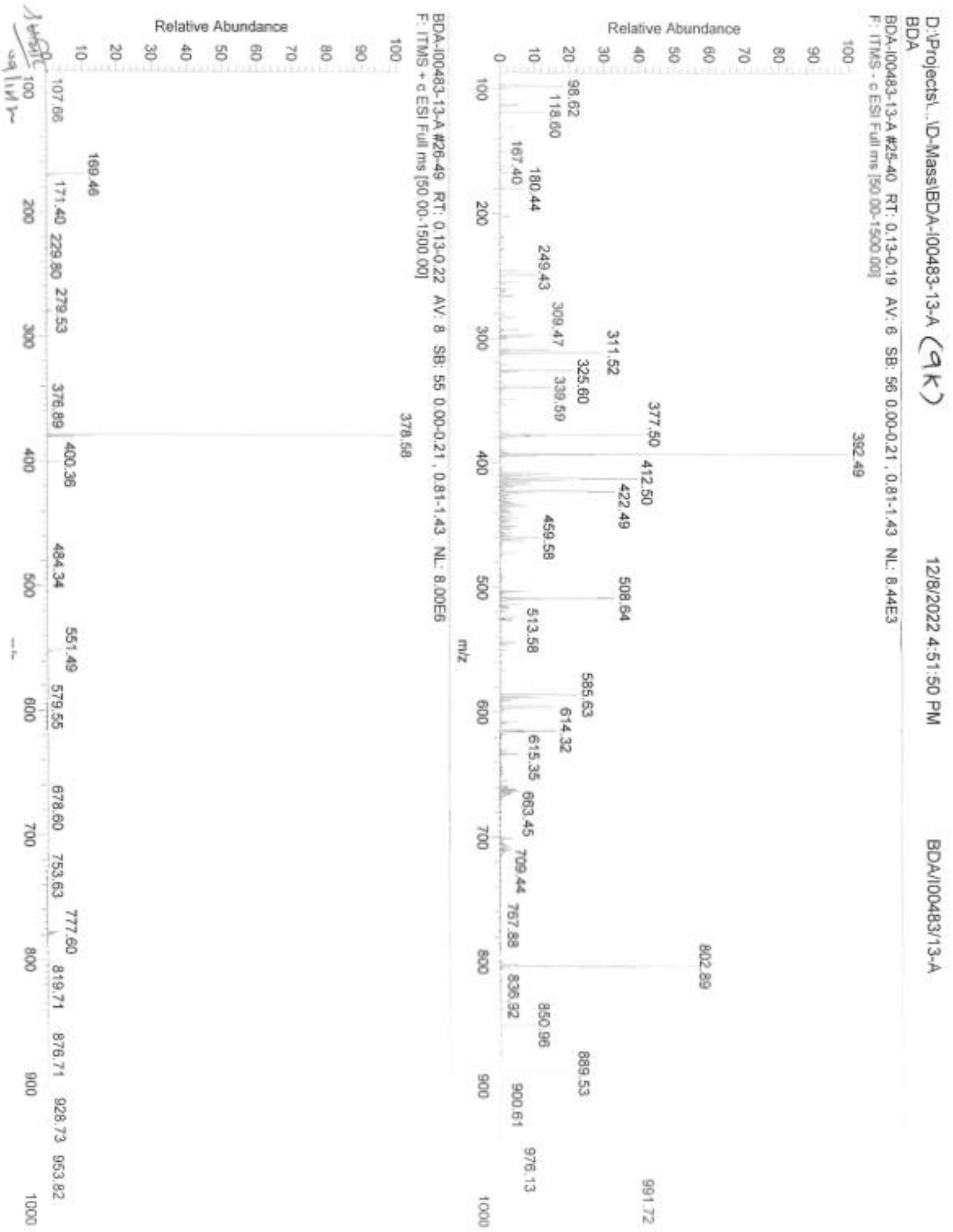

Mass spectrum of compound 9k

MICRO LABS LIMITED
API, R & D CENTRE, BOMMASANDRA (ML-27)
ANALYTICAL RESEARCH DIVISION

Sample Name	: BDA-4	Channel Description	2998 Ch1 225nm@4.8nm
Sample ID	: BDA/I00483/13-A (9k)	Aqu Method Set	: BDA 4 RS
Instrument ID	: ARD_HLC_171	Processing Method	: BDA_4_RS
Test Parameter	: RS	Vial No.	: 3
Column ID	: ARD/LCC/22/019	Injection Volume	: 5 uL
Sample Set Name	: 17122L013	Analyst	: pavithra
Run Time	: 75.0 Minutes		
Column Details	: Bakerbond Q2100 C18 (150mm*4.6mm,3.0μm)		
Project Name	: DEC_2022\ARD_HLC_171		

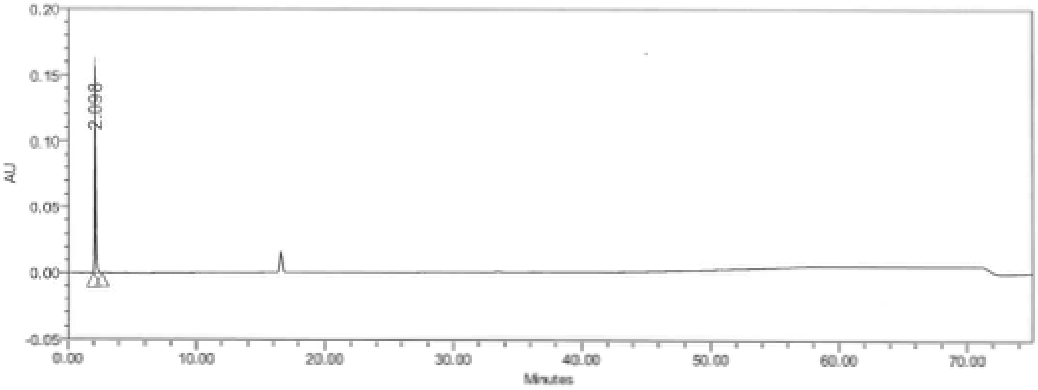

Sample Name BDA-4; Injection 1; Date Acquired 12-12-2022 15:30:59 IST; Date Processed 17-12-2022 14:24:10 IST; Result #

	Peak Name	RT	Height (μV)	Area	Int Type	RT Ratio	% Area	RRF	Corrected %Area
1	Peak1	2.10	156509	712052	BB	1.00	100.000	1.00	100.00
Sum				712052					

Result Sign Off

	SampleName	Result Id	Sign Off Full Name	Sign Off Date	Sign Off Reason
1	BDA-4	2721	Vidhi verma (Vidhi)	17-12-2022 14:29:30 IST	Sign Off Level 1, Reason: Submitted for Review
2	BDA-4	2721	Nakul Upadhyay (Nakul)	19-12-2022 15:50:27 IST	Sign Off Level 2, Reason: Reviewed and Approved

Processed By vidhi/Analyst

HPLC of compound 9k

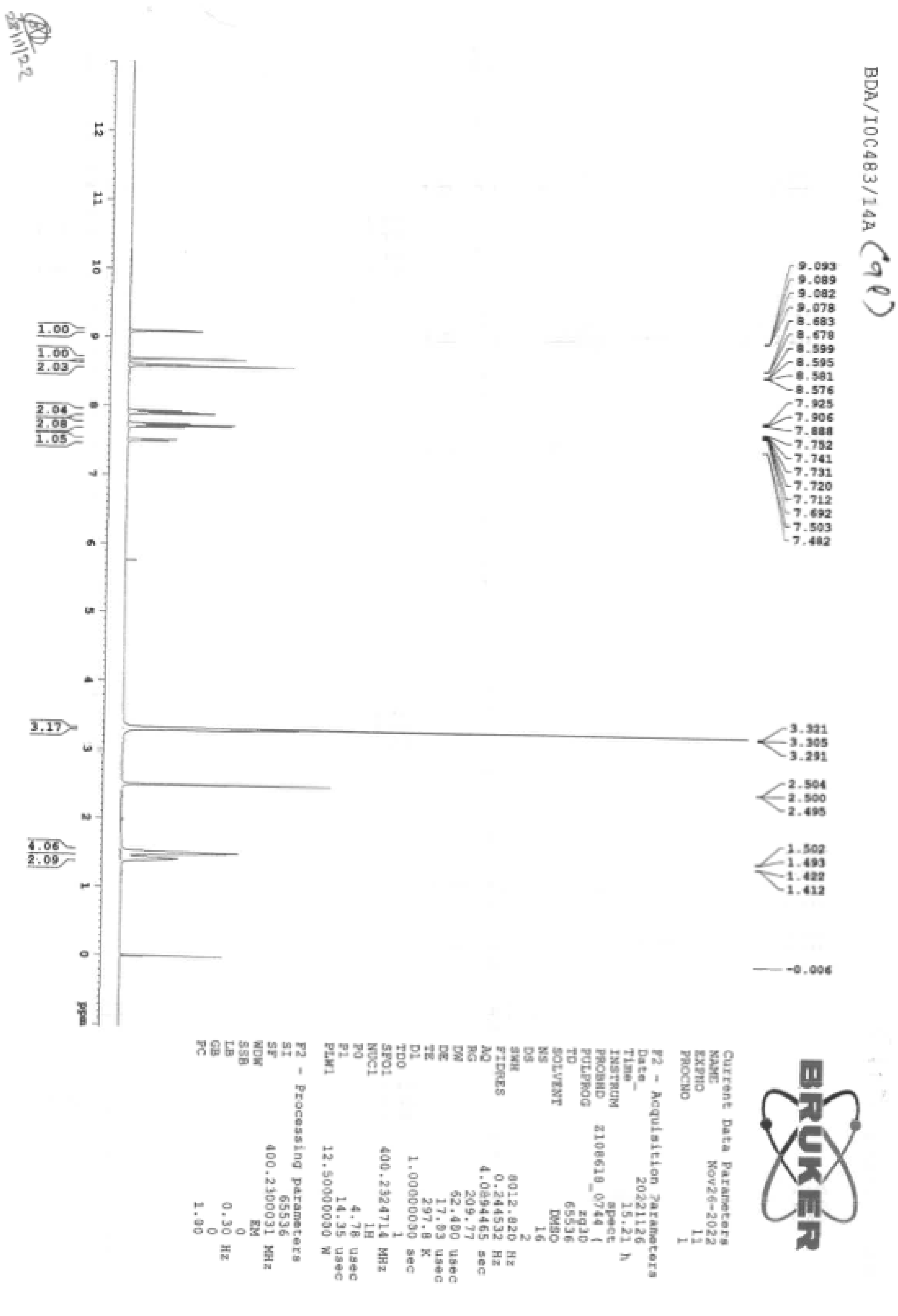

^{1}H-NMR spectrum of compound 9l

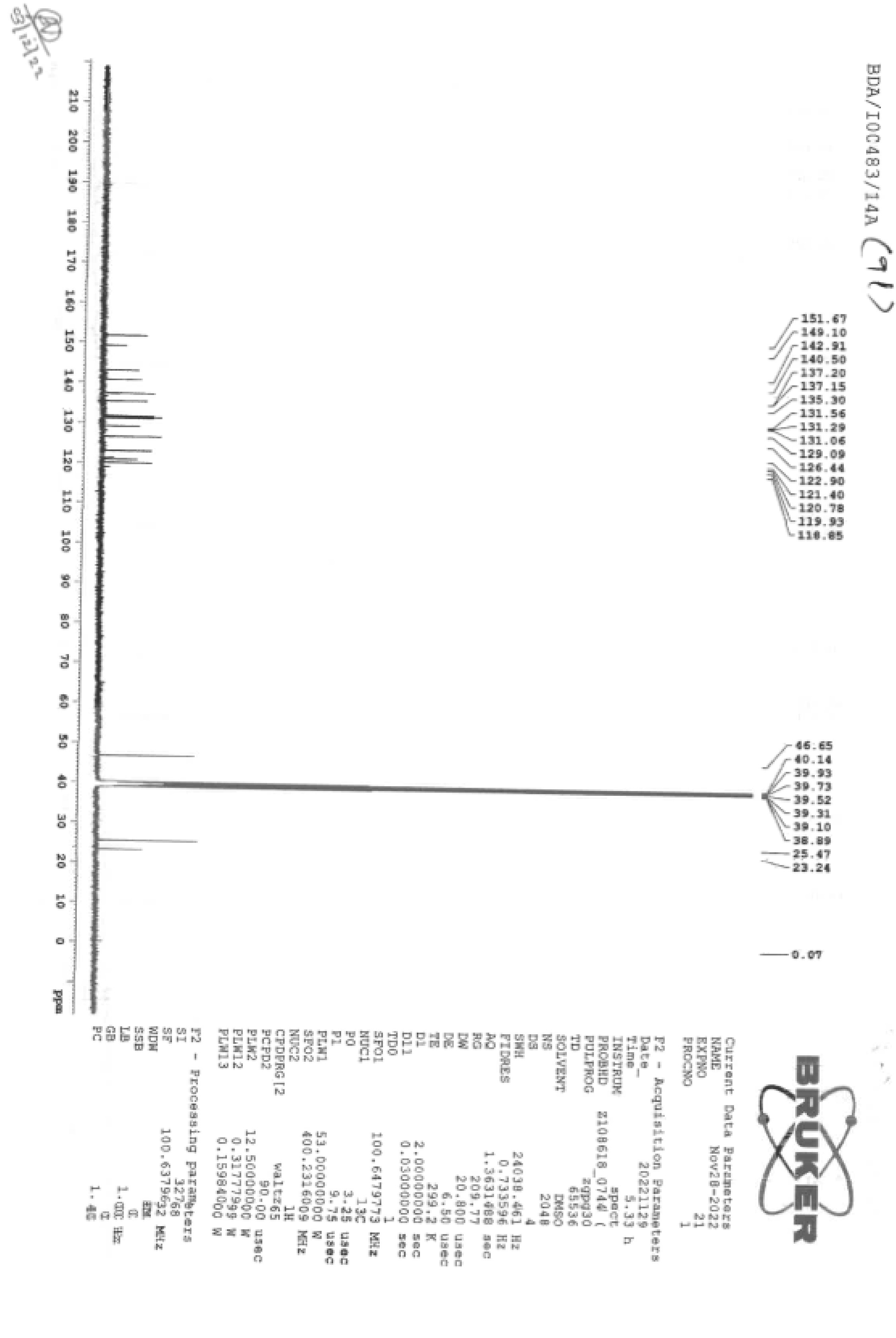

^{13}C NMR spectrum of compound 9l

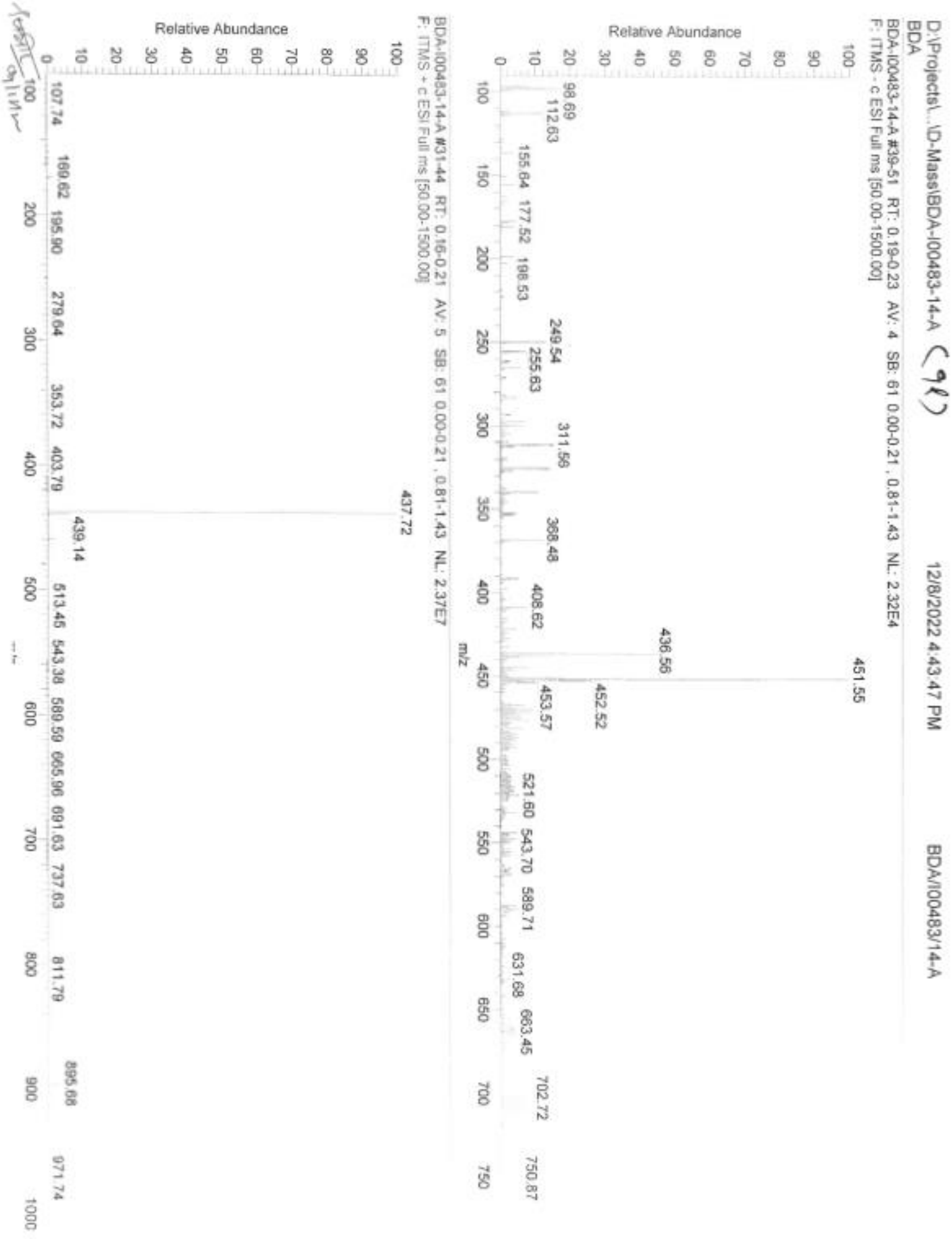

Mass spectrum of compound 9l

MICRO LABS LIMITED
API, R & D CENTRE, BOMMASANDRA (ML-27)
ANALYTICAL RESEARCH DIVISION

Sample Name : BDA-4
Sample ID : BDA/I00483/14-A (9l)
Instrument ID : ARD_HLC_171
Test Parameter : RS
Column ID : ARD/LCC/22/019
Sample Set Name : 17122L013
Run Time : 75.0 Minutes
Column Details : Bakerbond Q2100 C18 (150mm*4.6mm,3.0µm)
Project Name : DEC_2022\ARD_HLC_171

Channel Description 2998 Ch1 225nm@4.8nm
Aqu Method Set : BDA 4 RS
Processing Method : BDA_4_RS_IMP
Vial No. : 5
Injection Volume : 5 uL
Analyst : pavithra

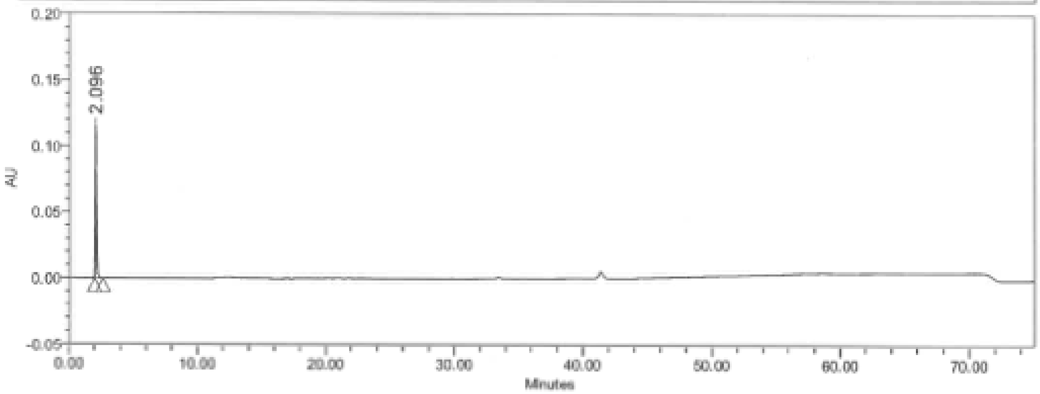

Sample Name BDA-4; Injection 1; Date Acquired 12-12-2022 18:02:40 IST; Date Processed 17-12-2022 14:26:54 IST; Result #

	Peak Name	RT	Height (µV)	Area	Int Type	RT Ratio	% Area	RRF	Corrected %Area
1	Peak1	2.10	114935	524350	BB	1.00	100.000	1.00	100.00
Sum				524350					

Result Sign Off

	SampleName	Result Id	Sign Off Full Name	Sign Off Date	Sign Off Reason
1	BDA-4	2726	Vidhi verma (Vidhi)	17-12-2022 14:29:30 IST	Sign Off Level 1, Reason: Submitted for Review
2	BDA-4	2726	Nakul Upadhyay (Nakul)	19-12-2022 15:50:27 IST	Sign Off Level 2, Reason: Reviewed and Approved

Processed By vidhi/Analyst

HPLC of compound 9l

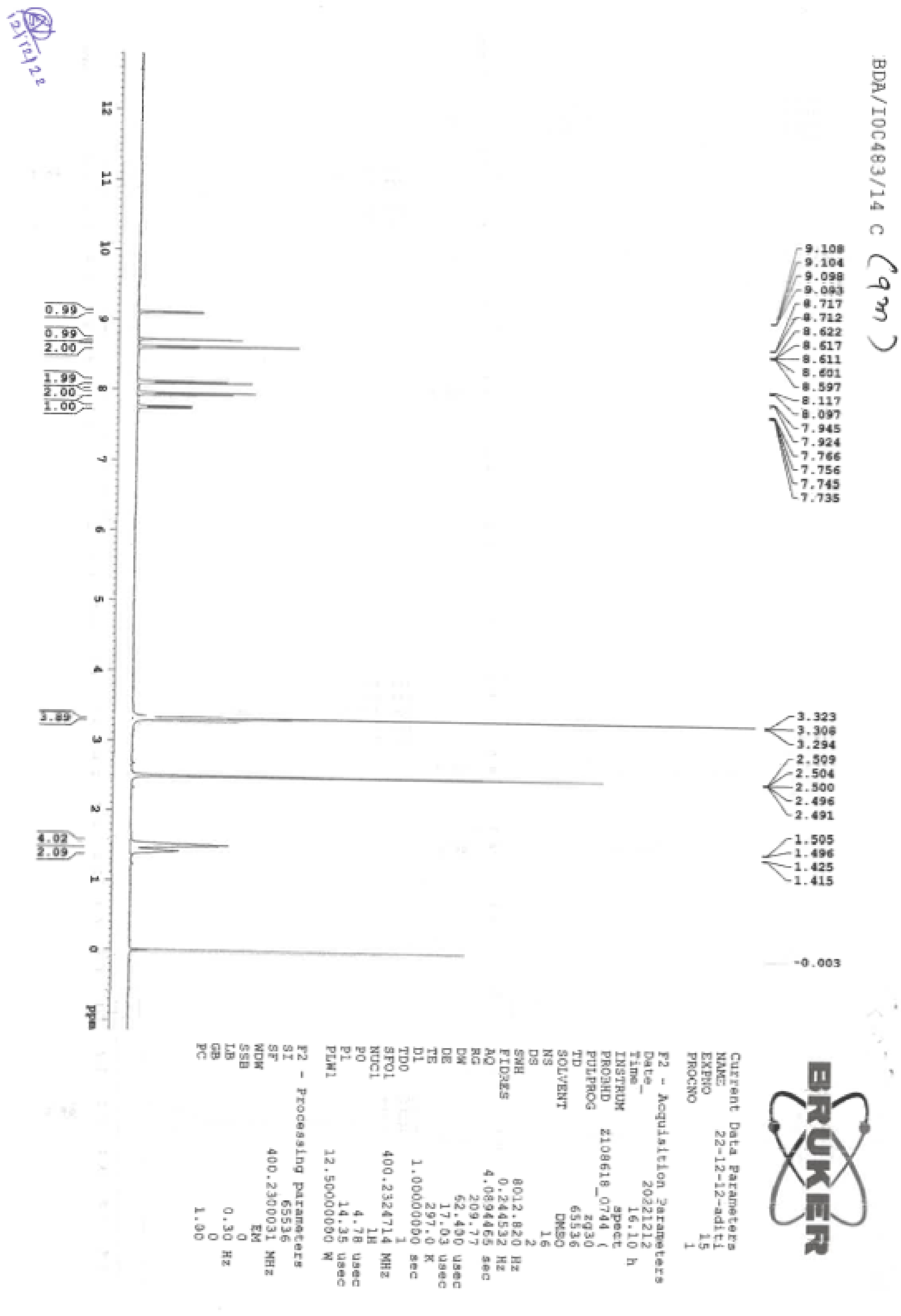

^{1}H-NMR spectrum of compound 9m

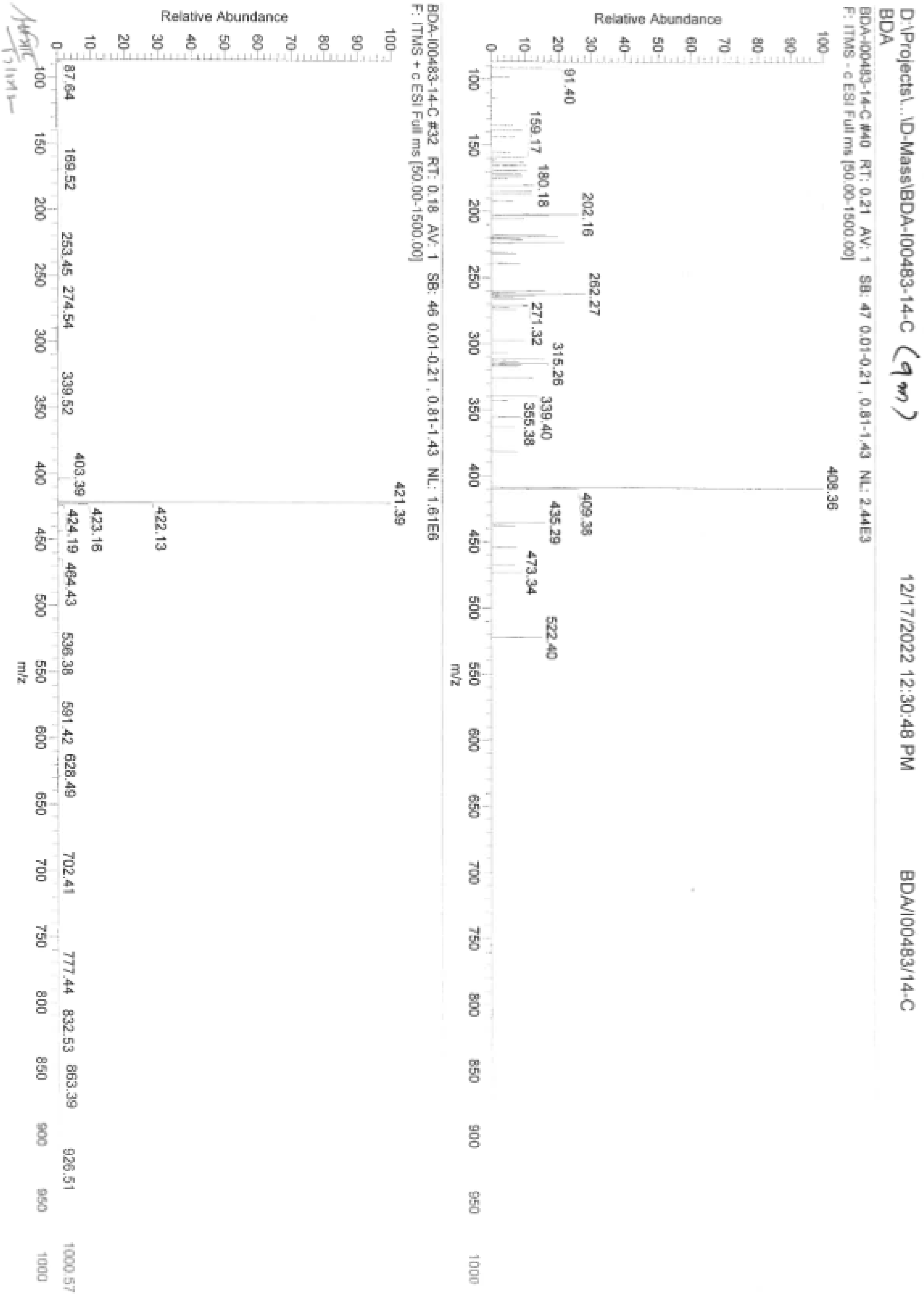

Mass spectrum of compound 9m

MICRO LABS LIMITED
API, R & D CENTRE, BOMMASANDRA (ML-27)
ANALYTICAL RESEARCH DIVISION

Sample Name	: BDA-4	Channel Description	Detector A 225nm
Sample ID	: BDA/I00483/014C (9m)	Aqu Method Set	: BDA 4 RS M 1
Instrument ID	: ARD_UPLC_085	Processing Method	: BDA_4_RS_M 1_SPL
Test Parameter	: RS (M-1)	Vial No.	: 3
Column ID	: ARD/LCC/22/017	Injection Volume	: 5 uL
Sample Set Name	: 08522L013	Analyst	: Sivanand.M
Run Time	: 75.0 Minutes		
Column Details	: Bakerbond Q2 100 C18 (150 mm * 4.6mm, 3.0µm)		
Project Name	: DEC_2022\ARD_UPLC_085		

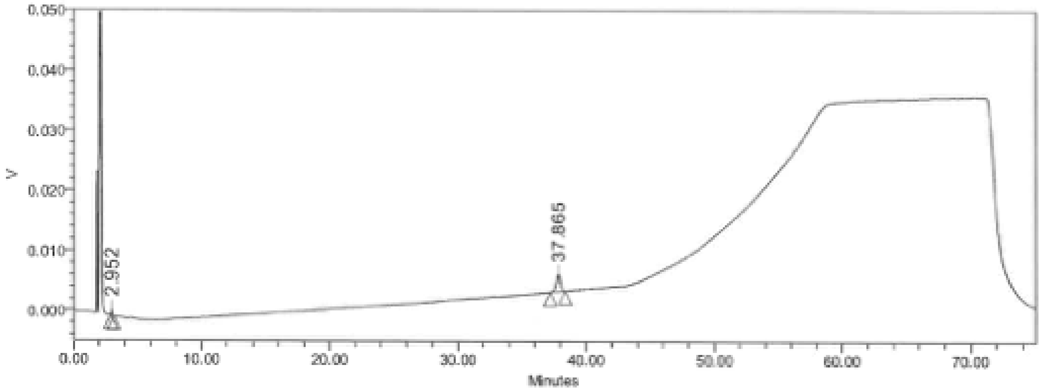

Sample Name BDA-4; Injection 1; Date Acquired 17-12-2022 19:07:53 IST; Date Processed 19-12-2022 09:06:03 IST; Result #

	Peak Name	RT	Height (µV)	Area	Int Type	% Area	RRF	Corrected %Area
1	Peak1	2.95	1010	4363	BB	5.804	1.00	5.80
2	Peak2	37.86	2799	70812	BB	94.196	1.00	94.20
Sum				75175				

Result Sign Off

	SampleName	Result Id	Sign Off Full Name	Sign Off Date	Sign Off Reason
1	BDA-4	2785	Mudigovda Sivanand (Sivanandm)	19-12-2022 08:45:19 IST	Sign Off Level 1, Reason: Submitted for Review
2	BDA-4	2785	Nakul Upadhyay (Nakul)	19-12-2022 11:10:30 IST	Sign Off Level 2, Reason: Reviewed and Approved

Processed By sivanandm/Analyst

HPLC of compound 9m

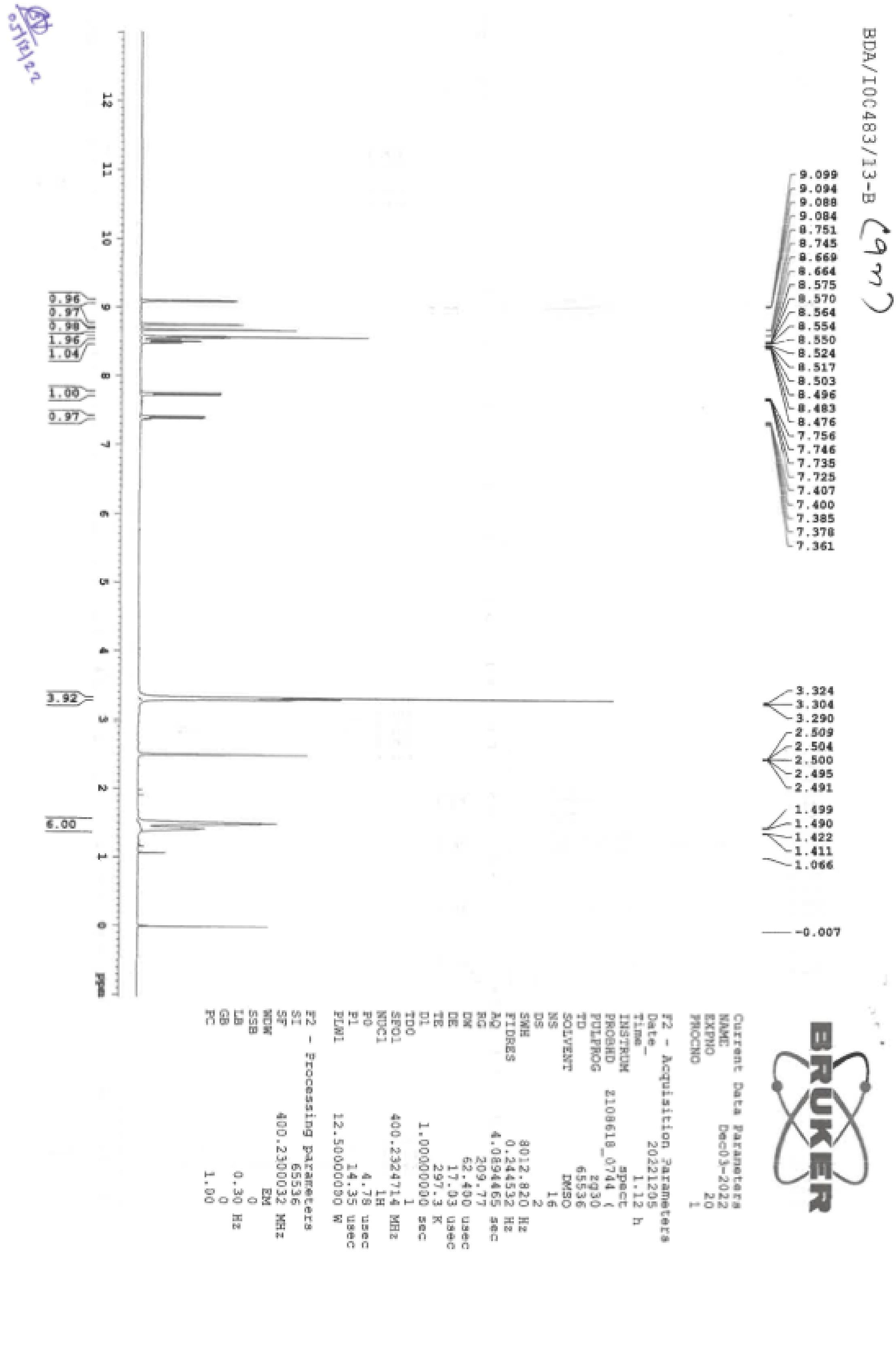

^{1}H-NMR spectrum of compound 9n

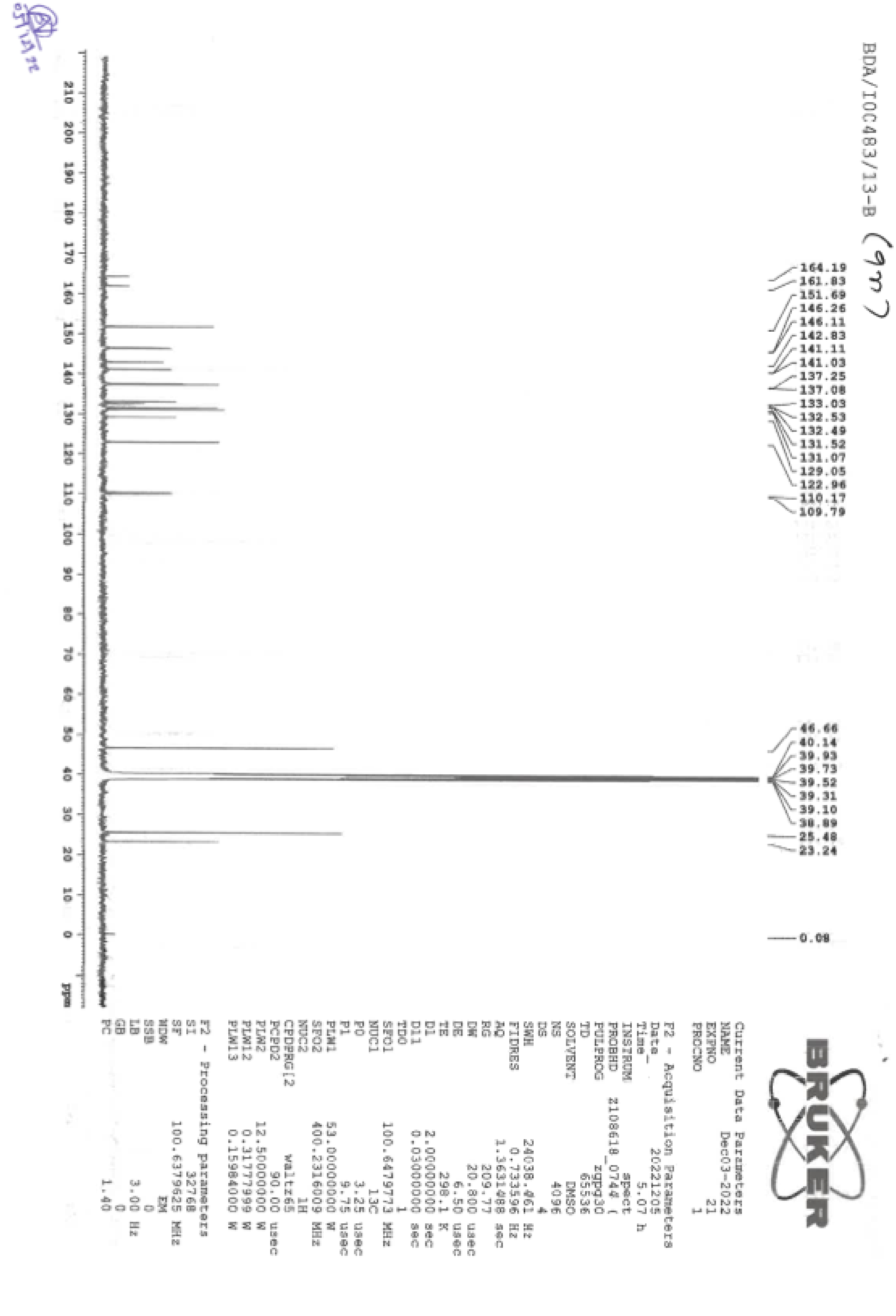

^{13}C NMR spectrum of compound 9n

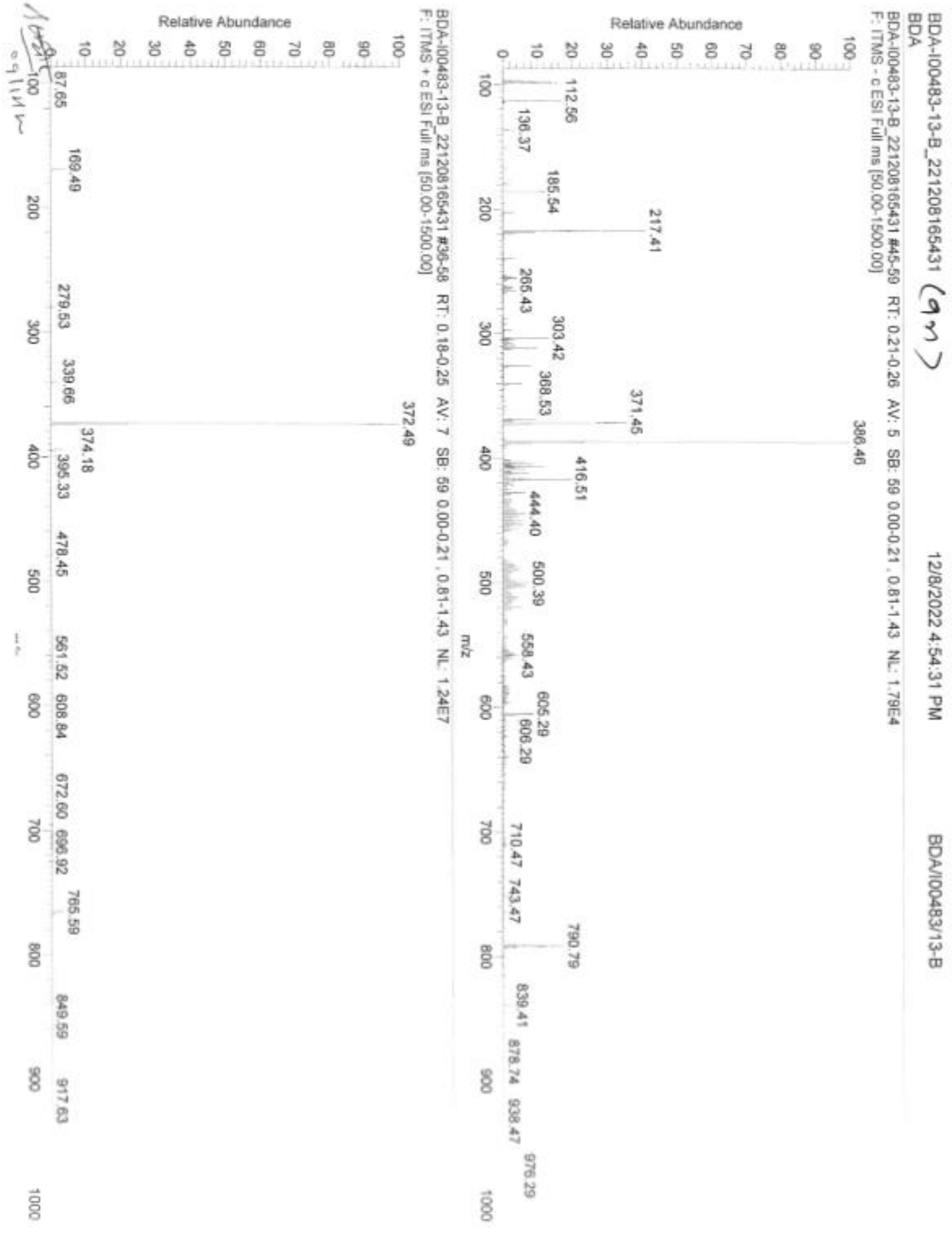

Mass spectrum of compound 9n

MICRO LABS LIMITED
API, R & D CENTRE, BOMMASANDRA (ML-27)
ANALYTICAL RESEARCH DIVISION

Sample Name	: BDA-4	Channel Description	2998 Ch1 225nm@4.8nm
Sample ID	: BDA/I00483/13-B (9n)	Aqu Method Set	: BDA_4_RS
Instrument ID	: ARD_HLC_171	Processing Method	: BDA_4_RS_IMP
Test Parameter	: RS	Vial No.	: 4
Column ID	: ARD/LCC/22/019	Injection Volume	: 5 uL
Sample Set Name	: 17122L013	Analyst	: pavithra
Run Time	: 75.0 Minutes		
Column Details	: Bakerbond Q2100 C18 (150mm*4.6mm,3.0μm)		
Project Name	: DEC_2022\ARD_HLC_171		

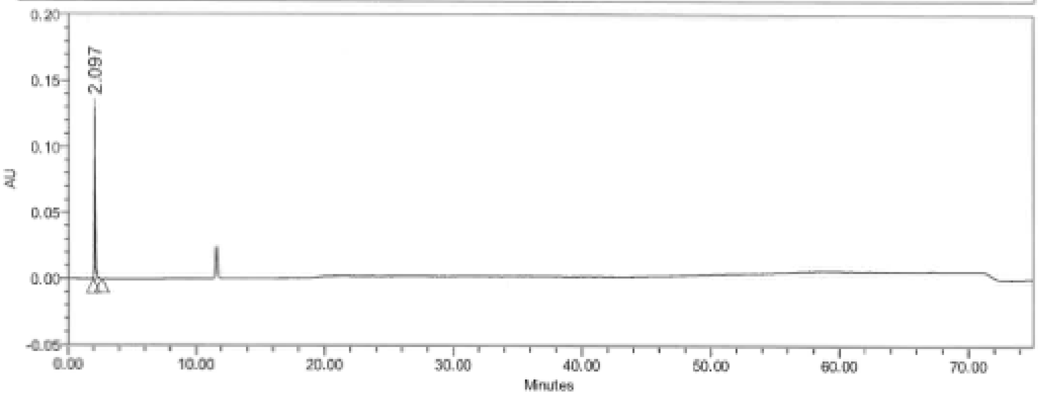

Sample Name BDA-4; Injection 1; Date Acquired 12-12-2022 16:46:48 IST; Date Processed 17-12-2022 14:26:00 IST; Result #

	Peak Name	RT	Height (μV)	Area	Int Type	RT Ratio	% Area	RRF	Corrected %Area
1	Peak1	2.10	129567	593638	BB	1.00	100.000	1.00	100.00
Sum				593638					

Result Sign Off

	SampleName	Result Id	Sign Off Full Name	Sign Off Date	Sign Off Reason
1	BDA-4	2725	Vidhi verma (Vidhi)	17-12-2022 14:29:30 IST	Sign Off Level 1, Reason: Submitted for Review
2	BDA-4	2725	Nakul Upadhyay (Nakul)	19-12-2022 15:50:27 IST	Sign Off Level 2, Reason: Reviewed and Approved

Processed By vidhi/Analyst

HPLC of compound 9n

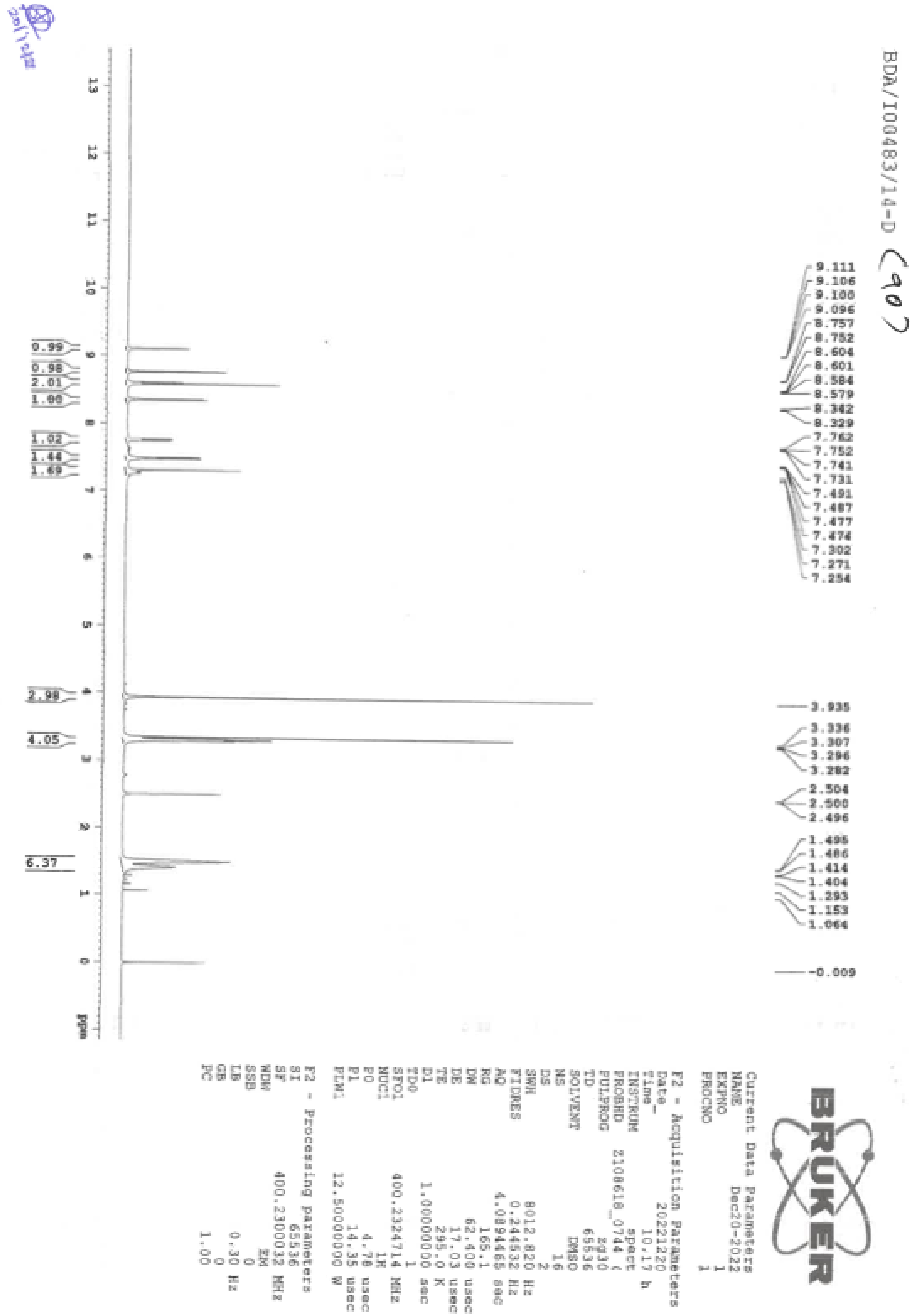

^{1}H-NMR spectrum of compound 9o

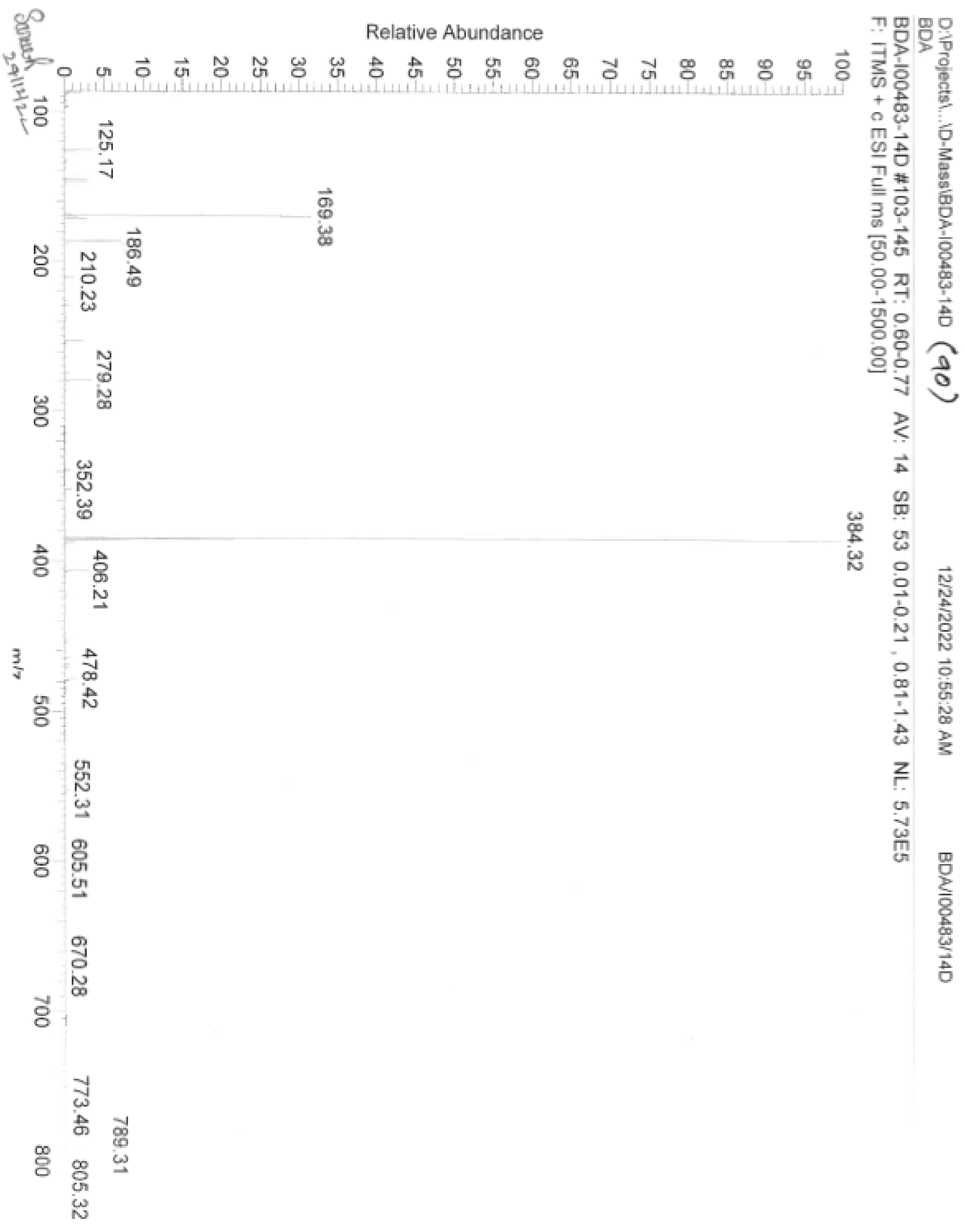

Mass spectrum of compound 9o

MICRO LABS LIMITED
API, R & D CENTRE, BOMMASANDRA (ML-27)
ANALYTICAL RESEARCH DIVISION

Sample Name : BDA-4
Sample ID : BDA/I00483/14D (9o)
Instrument ID : ARD_HLC_213
Test Parameter : RS (M1)
Column ID : ARD/LCC/21/032
Sample Set Name : 21322L012
Run Time : 75.0 Minutes
Column Details : Bakerbond Q2 100 C18 (150 mm * 4.6 mm , 3.0µm)
Project Name : DEC_2022\ARD_HLC_213

Channel Description UV Detector: Channel 1, 225 nm
Aqu Method Set : BDA 4 RS M1
Processing Method : BDA_4_RS_M1_SPL
Vial No. : 3
Injection Volume : 5 uL
Analyst : Sivanand.M

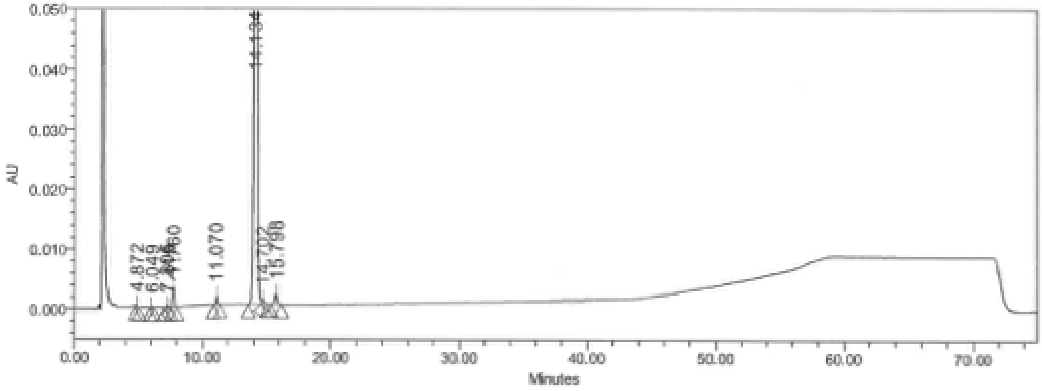

Sample Name BDA-4; Injection 1; Date Acquired 23-12-2022 14:40:40 IST; Date Processed 24-12-2022 10:27:48 IST; Result #

	Peak Name	RT	Height (µV)	Area	Int Type	RT Ratio	% Area	RRF	Corrected %Area
1	Peak1	4.87	290	2398	BB	0.34	0.084	1.00	0.08
2	Peak2	6.05	178	1280	BB	0.43	0.045	1.00	0.04
3	Peak3	7.21	376	3064	BV	0.51	0.107	1.00	0.11
4	Peak4	7.42	134	1008	VB	0.52	0.035	1.00	0.04
5	Peak5	7.76	3421	29621	BB	0.55	1.036	1.00	1.04
6	Peak6	11.07	1428	15433	BB	0.78	0.540	1.00	0.54
7	Peak7	14.13	206544	2765105	BV	1.00	96.720	1.00	96.72
8	Peak8	14.70	868	12897	VB	1.04	0.451	1.00	0.45
9	Peak9	15.80	1816	28058	BB	1.12	0.981	1.00	0.98

Processed By sivanandm/Analyst

HPLC of compound 9o

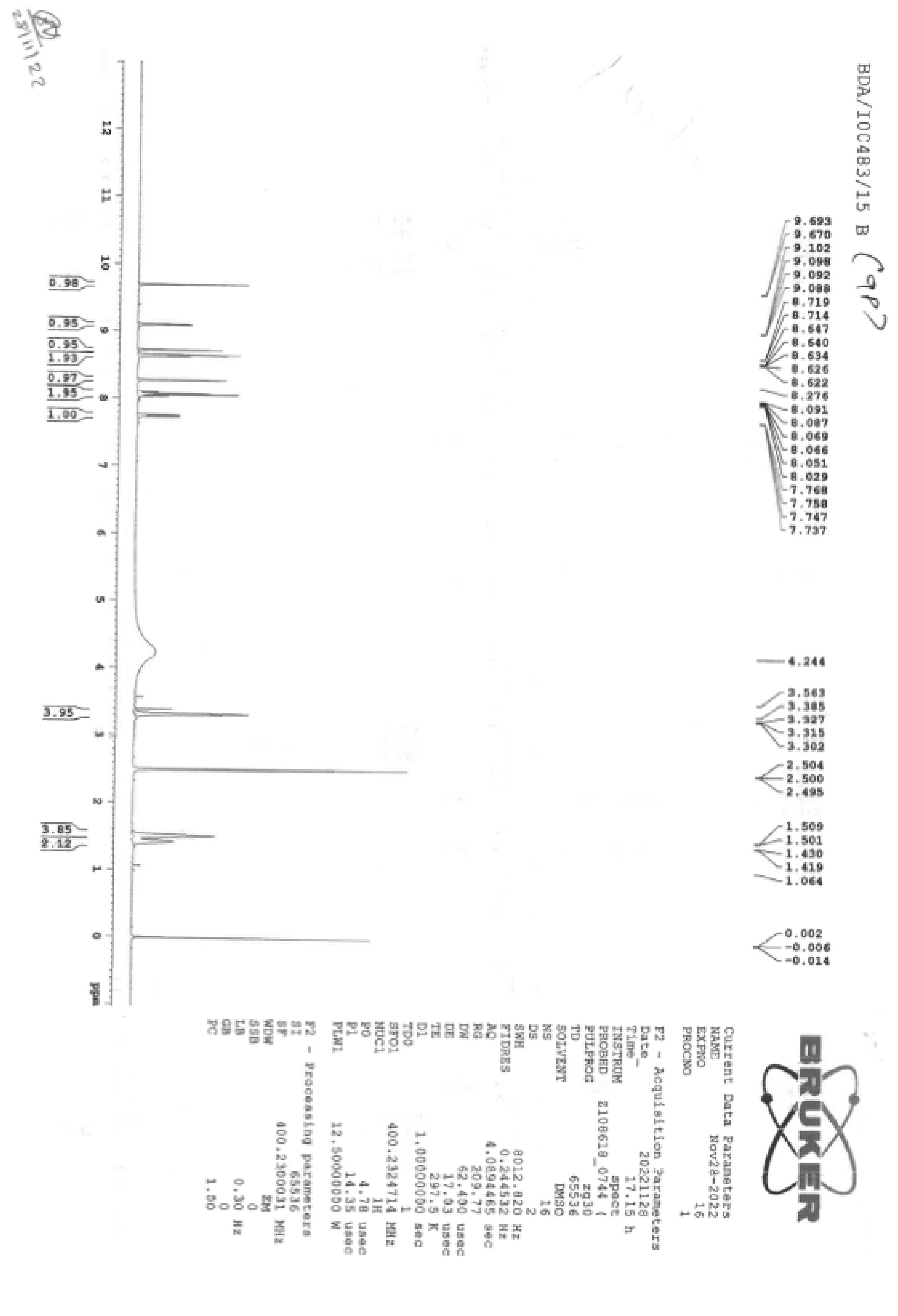

^{1}H-NMR spectrum of compound 9p

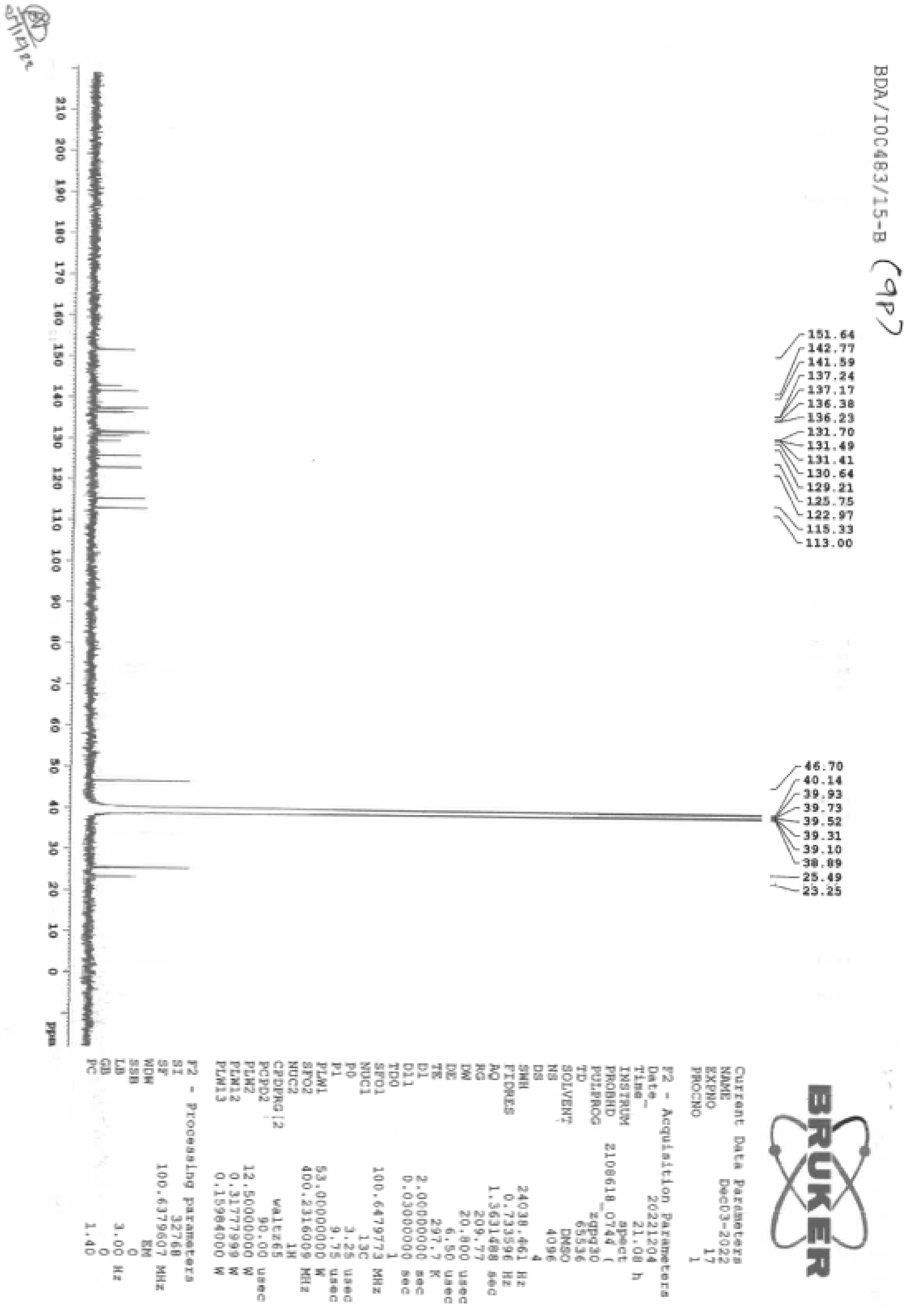

^{13}C NMR spectrum of compound 9p

MICRO LABS LIMITED
API, R & D CENTRE, BOMMASANDRA (ML-27)
ANALYTICAL RESEARCH DIVISION

Sample Name	: BDA-4	Channel Description	2998 Ch1 225nm@4.8nm
Sample ID	: BDA/100483/15-B (9P)	Aqu Method Set	: BDA 4 RS
Instrument ID	: ARD_HLC_171	Processing Method	: BDA_4_RS_IMP
Test Parameter	: RS	Vial No.	: 8
Column ID	: ARD/LCC/22/019	Injection Volume	: 5 uL
Sample Set Name	: 17122L013	Analyst	: pavithra
Run Time	: 75.0 Minutes		
Column Details	: Bakerbond Q2100 C18 (150mm*4.6mm,3.0µm)		
Project Name	: DEC_2022\ARD_HLC_171		

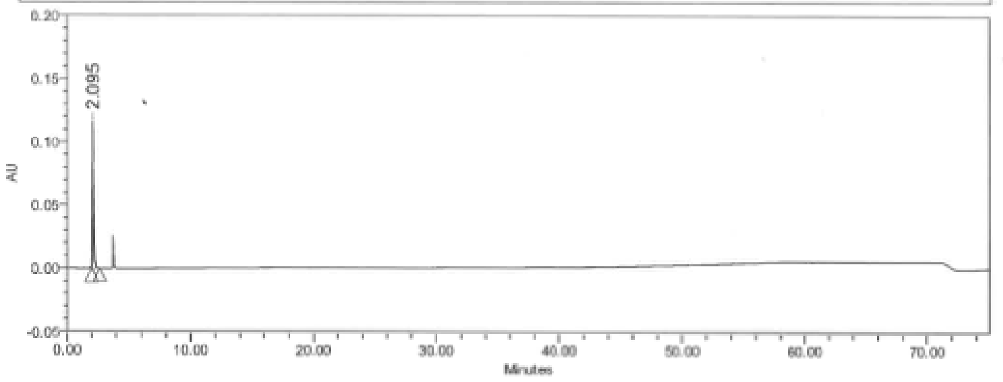

Sample Name BDA-4; Injection 1; Date Acquired 12-12-2022 21:50:08 IST; Date Processed 17-12-2022 14:27:44 IST; Result #

	Peak Name	RT	Height (µV)	Area	Int Type	RT Ratio	% Area	RRF	Corrected %Area
1	Peak1	2.10	115372	526734	BB	1.00	100.000	1.00	100.00
Sum				526734					

Result Sign Off

	SampleName	Result Id	Sign Off Full Name	Sign Off Date	Sign Off Reason
1	BDA-4	2729	Vidhi verma (Vidhi)	17-12-2022 14:29:30 IST	Sign Off Level 1, Reason: Submitted for Review
2	BDA-4	2729	Nakul Upadhyay (Nakul)	19-12-2022 15:50:27 IST	Sign Off Level 2, Reason: Reviewed and Approved

Processed By vidhi/Analyst

HPLC of compound 9p
